# Neoadjuvant oncolytic virus orienx010 and toripalimab in resectable acral melanoma: a phase Ib trial

**DOI:** 10.1038/s41392-024-02029-2

**Published:** 2024-11-22

**Authors:** Jiayong Liu, Xuan Wang, Zhongwu Li, Shunyu Gao, Lili Mao, Jie Dai, Caili Li, Chuanliang Cui, Zhihong Chi, Xinan Sheng, Yumei Lai, Zhichao Tan, Bin Lian, Bixia Tang, Xieqiao Yan, Siming Li, Li Zhou, Xiaoting Wei, Juan Li, Jun Guo, Lu Si

**Affiliations:** 1 Key Laboratory of Carcinogenesis and Translational Research (Ministry of Education), Department of Bone and Soft Tissue Sarcoma, Peking University Cancer Hospital and Research Institute, Beijing, China; 2 Key Laboratory of Carcinogenesis and Translational Research (Ministry of Education), Department of Melanoma and Sarcoma, Peking University Cancer Hospital and Research Institute, Beijing, China; 3 Key Laboratory of Carcinogenesis and Translational Research (Ministry of Education), Department of Pathology, Peking University Cancer Hospital and Research Institute, Beijing, China; 4 Key Laboratory of Carcinogenesis and Translational Research (Ministry of Education), Department of Radiology, Peking University Cancer Hospital and Research Institute, Beijing, China; 5 Key Laboratory of Carcinogenesis and Translational Research (Ministry of Education), Department of Genitourinary Oncology, Peking University Cancer Hospital and Research Institute, Beijing, China

**Keywords:** Skin cancer, Skin cancer

## Abstract

Neoadjuvant PD-1 inhibitor is promising in cutaneous melanoma but remains unknown in acral melanoma (AM). This phase Ib trial study (Clinicaltrials.gov NCT04197882) assessed the efficacy and safety of the combination of neoadjuvant oncolytic virus orienX010 (ori) and anti-PD-1 toripalimab (tori) for resectable AM. Thirty patients of stage III/IV received neoadjuvant therapy of ori and tori for 12 weeks before surgery, followed by adjuvant treatment with tori for 1 year. Primary endpoints were radiographic and pathological response rates, with secondary endpoints of 1- and 2-year recurrence-free survival (RFS) rates, event-free survival (EFS) rates, and safety. Twenty-seven completed surgery and tori adjuvant treatment and median follow-up was 35.7 months. Radiographic and pathological response rates were 36.7% and 77.8%, with complete response rates of 3.3% and 14.8%, 1- and 2-year RFS rates of 85.2% and 81.5%, and 1- and 2-year EFS rates of 83% and 73%, respectively. Adverse events occurred in all patients, mainly grade 1–2. There was no correlation between PET/CT evaluation and pathological response or progression-free survival/overall survival. Patients with pathological response showed tumor beds with high tertiary lymphoid structures (TLSs) and tumor-infiltrating lymphocytes (TILs). Cytokines and chemokines analysis showed the combination therapy significantly increases the secretion of proinflammatory cytokines and chemokines in both responders and non-responders. Therefore, neoadjuvant ori and tori demonstrated promising antitumor activity with high response rates and high 2-year RFS/EFS for AM with acceptable tolerability.

## Introduction

Acral melanoma (AM) is an aggressive subtype of melanoma.^1^ It has a low incidence in the Caucasian population, but a high incidence in the Chinese population, accounting for about 40% of melanoma cases.^2^ Despite notable advances in melanoma treatment, patients with AM have received limited benefit from popular immunotherapies, and lack established neoadjuvant therapeutic regimens.^3^ Thus, the prognosis for AM remains poor and shows a significantly severe immunosuppressive state.^4^ In addition, tumor mutational burden (TMB) was significantly different between acral and cutaneous melanoma, which makes AM less immunogenic and less responsive to immunotherapy.^5^ The treatment response of advanced AM to anti-PD-1 immunotherapy is significantly lower than that of cutaneous melanoma.^6–8^ A retrospective study showed that patients with AM treated with checkpoint inhibitors had a median overall survival (OS) of 17 months, significantly shorter than that of patients with cutaneous melanomas (median OS of 46 months *P* = 0.047).^9^ However, the response was even worse in Asian populations. In Keynote 151 which conducted in China, pembrolizumab monotherapy had an objective response rate (ORR) of 15.8%.^10^ A multi-center trial in Japan of patients treated with nivolumab reported an ORR of 19%, and a median OS of 15.6 months.^11^

In spite of advances in treatment modalities, patients with stage IIIB-IVM1a melanoma remain at high risk of recurrence after complete surgical resection with curative intent.^12^ It remains critical to pursue other treatments aimed at improving OS by preventing disease recurrence.^13^ Theoretically, neoadjuvant immunotherapy has the potential to provoke a more robust immune response, given the heightened presence of tumor antigens compared to adjuvant strategies.^14^ Recent studies focusing on neoadjuvant interventions have demonstrated encouraging improvements in patient outcomes relative to surgery followed by adjuvant therapy.^15^ Compared with neoadjuvant-targeted therapy,^16^ neoadjuvant immunotherapy had much better disease-free survival (DFS).^17^ Notably, these studies have also yielded valuable prognostic insights derived from the pathological scrutiny of tumors. With the emergence of new systemic treatment options, the field of neoadjuvant immunotherapy for stage III/IV melanoma is rapidly developing. The recently published NADINA trial marks a milestone in the adjuvant treatment of melanoma. Compared to adjuvant therapy, neoadjuvant therapy shows more favorable outcomes for patients with stage III melanoma. This may have a significant impact on the treatment protocols for melanoma in the future.^18^ However, while neoadjuvant immunotherapy has demonstrated remarkable efficacy in cutaneous melanoma, the quest for an effective neoadjuvant regimen for AM is still ongoing. Given the limited efficacy of anti-PD-1 monotherapy in the treatment of AM, combination therapy is a promising approach.

Oncolytic virus therapy is an effective immunotherapeutic option. Local oncolytic viral immunotherapy, exemplified by talimogene laherparepvec (T-VEC), has been approved for treating unresectable stage IIIB-IVM1a melanoma with nodal, subcutaneous, or cutaneous metastases.^19^ A multitude of preclinical and clinical studies have highlighted T-VEC’s capacity to remodel the tumor microenvironment, rendering the immune-deserted “cold” tumors into immunogenic “hot” tumors, thus enhancing susceptibility to immune checkpoint inhibitor (ICI) treatment.^20–22^ In a phase Ib trial, patients with unresectable stage IIIB-IV cutaneous melanoma were subjected to intralesional T-VEC followed by systemic pembrolizumab.^23^ Most patients experienced an increase in circulating T cells after T-VEC was administered. After treatment with pembrolizumab and T-VEC, the tumors of responders showed significant increases in PD-L1 protein levels, IFN-γ gene expression, and T cells. And the benefit of T-VEC also showed in neoadjuvant trail.^24^

OrienX010 (ori), a genetically modified Herpes Simplex Virus (HSV) type 1-derived oncolytic virus encoding human granulocyte-macrophage colony-stimulating factor (GM-CSF) which was collected from the oral cavity,^25^ has been strategically designed for direct injection into melanoma lesions. A phase Ib study of ori in unresectable stage IIIC–IV melanoma patients showed ORR as high as 28.6% in the high-dose cohort (10 mL of 8 × 10^7^ pfu/mL). Remarkably, the AM subset achieved survival benefits similar to those of cutaneous melanoma.^26^ In the present study, we conducted a phase Ib trial to investigate the efficacy and safety of ori in combination with tori as neoadjuvant treatment for AM with completely resectable stage III and IVM1a AM.

## Results

### Patient characteristics and disposition

Between July 2019 and December 2020, 43 candidates signed informed consent before enrollment, and 33 eligible patients were enrolled in the trial, with 30 patients received study treatment and 3 patients withdrawing. The main cause of screening failure was distant metastasis. Among the 30 patients who received study treatment, the median age was 57 (range 21–72), and 47% were male. Stage disposition was 40% stage IIIB, 47% IIIC and 13% IVM1a. Only 20.0% of patients had BRAF-mutated melanoma. The median sum of target lesion diameters was 28 mm (Table 1).

**Table 1:** Patient’s characteristics

Characteristics	
Gender, *N* (%)	
Female	16 (53%)
Male	14 (47%)
Age, median (range)	57 years (21–72)
ECOG, *N* (%)	
0	25 (83.3%)
1	5 (16.7%)
Stage, *N* (%)	
IIIB	12 (40%)
IIIC	14 (47%)
IIID	0
IVM1a	4 (13%)
Lesion site, *N* (%)	
Sub-ungual	3 (10%)
Sole/palm	27 (90%)
Tumor burden, median (range), (mm)	28 (10–80)
Mutation, *N* (%)	
BRAF mutation	3/15 (20.0%)
NRAS mutation	3/15 (20.0%)
CKIT mutation	2.15 (13.3%)
Wilde type and others	7/15 (66.7%)
LDH, *N* (%)	
High	10 (33.3%)
Normal	20 (66.7%)

*ECOG* Eastern Cooperative Oncology Group, *LDH* lactate dehydrogenase

**Fig. 1:**
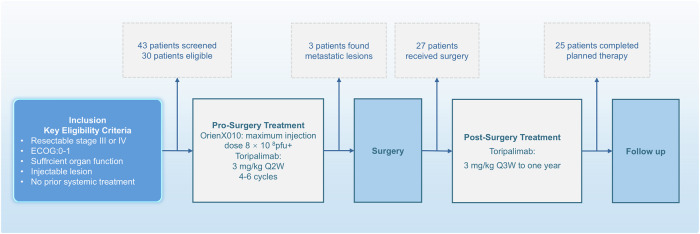
Study design and consort diagram with patient disposition

The average course of neoadjuvant ori and tori was 5.6 doses (4–6 doses), and the average ori dose was 8.9 mL (3–10 mL). Three patients (all primary feet) did not undergo surgery due to the development of distant metastatic disease during neoadjuvant therapy (two visceral organs and one lymph node). Of the remaining 27 patients who underwent surgery, all 27 patients subsequently received adjuvant therapy. Nevertheless, two patients chose to discontinue adjuvant therapy due to toxicity (Fig. 1).

### Clinical activities

#### Radiographic response

**Fig. 2:**
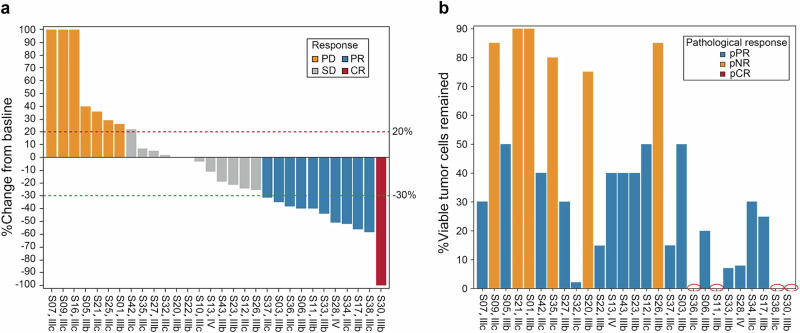
Response data. **a**: Radiographic response; **b**: Pathologic response: Red circle indicates no tumor residue, pCR

According to the standard of Response Evaluation Criteria in Solid Tumors (RECIST 1.1) and pathological response criteria, the radiographic response rate was 36.6% (1 CR and 10 PR); 40% had SD and 23.4% had PD (Fig. 2a, Table 2) in the 30 patients received study treatment. Pathological responses were frequently inconsistent with radiographic response (Supplementary Table S1). For example, among the 12 patients who achieved major pathological response (pCR + near pCR), only three had radiographic PR.

**Table 2:** Radiographic and pathological responses

Pathological response	Subjects (%), (*n* = 27)^a^	Radiographic responses	Subjects (%), (*n* = 30)^b^
Pathological response rate	21 (77.8)	Radiographic response)	11 (36.7)
pCR	4 (14.8)	CR	1 (3.3)
near-pCR	5 (18.5)		
pPR	12 (44.5)	PR	10 (33.3)

^a^*n* = 27 27 patients underwent surgery

^b^*n* = 30 All 30 patients underwent treatment

#### Pathological response

As defined in the methodology section regarding pathological responses, of the 30 patients treated, 27 (90%) underwent surgery; 4 (14.8%) achieved pCR, 5 (18.5%) near pCR, 12 (44.5%) pPR and 6 (22.2%) had no pathological response (Fig. 2b, Table 2). Major pathological responses were achieved in 33.3% of patients, and any pathological responses (pCR + near pCR + pPR) were achieved in 77.8% of patients. In five patients with surgery in both the lymph node and primary lesion, there was a mix of pathological responses. Of these five patients, four had pCR in the primary lesion, but only one showed near-pCR in lymph node lesions.

Responses were observed in both injected and non-injected lesions. Of the 48 measurable lesions, 40 had direct injection of ori, and 25 (62.5%) had radiographic regression. Radiographic and pathological responses were observed in eight non-injectable lesions (all regional). Five of eight patients (62.5%) had a radiographic response, and five of six patients (83.3%) had a pathological response.

#### RFS

**Fig. 3:**
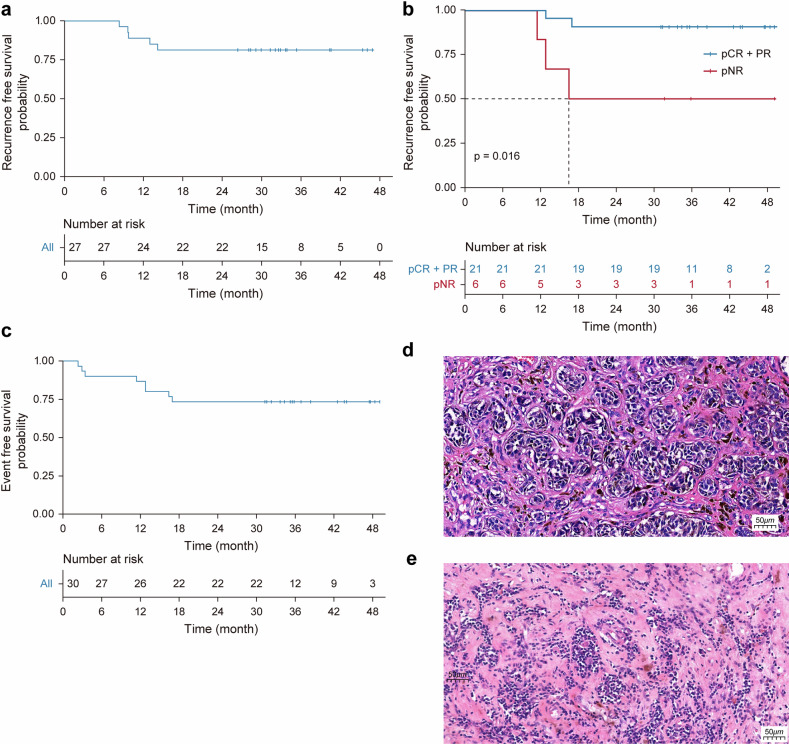
Recurrence-free survival curves. **a** Relapse-free survival (time from surgery to recurrence in patients that underwent surgery). **b** Probability of being relapse-free based on any pathologic response versus no pathologic response. **c** Event-free survival rates (time from treatment initiation to recurrence in all patients). **d** Case 11, pre-treatment histologic image: hematoxylin and eosin staining of tumor biopsy specimen prior to treatment reveals massive atypical melanoma cells within the tissue (magnification 200×). **e** Case 11, post-treatment histologic image: Hematoxylin and eosin staining of the surgical resection specimen demonstrates no evidence of tumor cells but a pronounced TIL infiltration post-neoadjuvant therapy (magnification 200×)

The cutoff- date was on 30th April 2023, with a median follow-up of 35.7 months (range 12.0–49.1 months). The 1-year and 2-year RFS rates were 85.2% and 81.5%, respectively (Fig. 3a). The median RFS was not reached, with a mean RFS of 40.3 months. At the last follow-up, five patients had recurrences, including three with lymph node metastasis, one with lung metastasis, and one with intestinal metastasis. Among the five recurrence patients, three had no pathological response (all lymph node metastasis), one had pPR, and one had major PR/near pCR.

In patients with pathological response, the median RFS was not reached (mean 43.5 months), which was much higher than 28.8 months (median 14.2 months) for patients with pathologic non-response (pNR) (*p* = 0.016; Fig. 3b). Additionally, there was no difference between the pCR + near pCR group and the pPR group (mean 40.5 months vs. 46.4 months).

#### Event-free survival

The 1-year and 2-year EFS rates were 83% and 73%, respectively (Fig. 3c). The median EFS was reached, with a mean EFS of 35.7 months. The subgroup analyses showed that the median EFS was significantly longer in patients with normal LDH levels compared with high LDH at baseline, not reached versus 12.75 months (confidence interval [CI]: 4.947–20.553, nominal *p* = 0.001). There was no significant difference among patients with different ages, gender, stage, gene mutations, and Eastern Cooperative Oncology Group (ECOG).

#### Overall survival

Up to the last follow-up, four patients had died due to tumor progression, three of whom had not undergone surgery after neoadjuvant therapy because of the occurrence of distant metastatic disease. The 12-month and 24-month OS were 96% and 90%, respectively (supplementary Fig. S1a). The mean OS was 45.3 months (median had not been reached), while an improved OS was seen for patients with pathologic response (median had not been reached; 1-year and 2-year OS of 100%) compared to those with pNR (median had not been reached; 1-year and 2-year OS of 83.3%; nominal *p* = 0.061), but the difference did not achieve statistical significance (Supplementary Fig. S1b). In addition, there was no significant difference between the pCR+ near pCR group and the pPR group. The subgroup analysis showed that patients with normal baseline LDH levels had significantly longer median OS (not reached versus 37.5 months; CI: 22.158–52.699, *p* = 0.005). There was no significant difference in age, sex, stage, gene mutations, and ECOG.

#### Toxicity

The combination treatment was well tolerated (Table 3). All patients experienced treatment-related adverse events (TRAEs), most of which were grade 1 or 2 (25/30, 83.3%). Four patients (13.3%) reported grade 3 Adverse Events (AEs) (2 soft tissue infections, 1 elevated aspartate aminotransferase [AST], and 1 peripheral neuropathy). Of all the AEs, 93.3% were attributed to the combination treatment, while 13.3% were clearly related to ori alone. Only two cases of grade 3 treatment‐related AEs (1 elevated AST and 1 peripheral neuropathy) were observed, and both were in the adjuvant setting.

**Table 3:** Treatment-related adverse events

Treatment related AEs occurring in >5% of subjects or ≥Grade 3 (*N* = 30)			
	OrienX010 + Toripalimab, *n* (%)		
Adverse events (AEs, CTCAE v. 5.0)			
	All grades	Grade 1–2	Grade 3
All AEs	28 (93.3)	28 (93.3)	2 (6.6)
Fever	22 (73.3)	22 (73.3)	0
Rash	11 (36.7)	11 (36.7)	0
Blood bilirubin increased	6 (20.0)	6 (20.0)	0
Chills	6 (20.0)	6 (20.0)	0
Vitiligo	6 (20.0)	6 (20.0)	0
Nausea	3 (10.0)	6 (20.0)	0
Anorexia	3 (10.0)	3 (10.0)	0
Unconjugated bilirubin increased	3 (10.0)	3 (10.0)	0
Alanine aminotransferase increased	5 (16.7)	3 (10.0)	0
Conjugated bilirubin increased	2 (6.7)	2 (6.7)	0
GGT increased	2 (6.7)	2 (6.7)	0
Peripheral neuropathy	1 (3.3)	0	1 (3.3)

	OrienX010, (*n*%)		
Adverse events (AEs, CTCAE v. 5.0)			
	All grades	Grade 1–2	Grade 3
All AEs	4 (13.3)	4 (13.3)	0
Fever	3 (10.0)	3 (10.0)	0
Injection site reaction	2 (6.7)	2 (6.7)	0
Myalgia	2 (6.7)	2 (6.7)	0

#### PET-CT

Twenty-seven patients underwent 18F-FDG PET/CT at two scan intervals: prior to the onset of treatment (SCAN-1), and 8–12 weeks before surgery (SCAN-2). The median maximum standardized uptake value (SUVmax) at SCAN-1 was 6.7. We used 14.7% (adopted from Cho et al.) as the cutoff value for changes in SUVmax between SCAN-2 and SCAN-1. SUVmax decreased between SCAN-1 and SCAN-2 in 14/27 (46.7%) patients. The evaluation of preoperative 18F-FDG PET/CT showed no correlation between PET parameters and postoperative pathology response (*p* = 0.769). Patients with decreased SUVmax (>14.7%) after neoadjuvant therapy did not show better PFS (*p* = 0.405) or OS (*p* = 0.245).

#### Additional histopathologic assessments

A total of 27 patients underwent complete tumor resection after treatment in this clinical trial. Treated tumor specimens were assessed for histopathologic characteristics indicative of therapeutic response, including tumor beds identified by pathologic analysis with hyaline fibrosis, TLSs, Ki-67%, and dense TILs. The area of regression was distinguished by the presence of robust immune infiltrates displaying signs of activation, such as TLSs, dense accumulations of TILs, infiltration by plasma cells, and the formation of granulomas, in addition to other attributes such as hyaline fibrosis after treatment. Patients with a pathological response had higher TIL infiltration (95.2% vs 50.0%, *p* = 0.006) and more TLS (70.6% vs 16.7%, *p* = 0.022) compared to those with pNR (Fig. 3d, e). No hyaline fibrosis or Ki67% decrease was observed in pNR patients’ tumor beds after neoadjuvant therapy, but there was no statistically significant difference between the two groups (Supplementary Table S2). Patients with high TIL infiltration showed longer RFS after surgery (mean 42.1 months vs 29.0 months, *p* = 0.084). However, hyaline fibrosis and TLSs did not show any improvement during RFS (Supplementary Table S2).

#### Cytokines and chemokines analysis

We leveraged the Olink technology to quantify circulating inflammatory proteins (liquid biopsy). 32 serum samples were collected from 16 patients (8 pCR + near pCR and 8 pNR) before (baseline) and after treatment (6 weeks:C3) for analysis. Analysis showed the combination of oncolytic virus and anti-PD-1 monoclonal antibody therapy significantly increased the secretion of cytokines and chemokines (IFN-gamma, GZMA, CXCL10, and IL12) in both groups of patients (Supplementary Fig. S2).

## Discussion

**Fig. 4:**
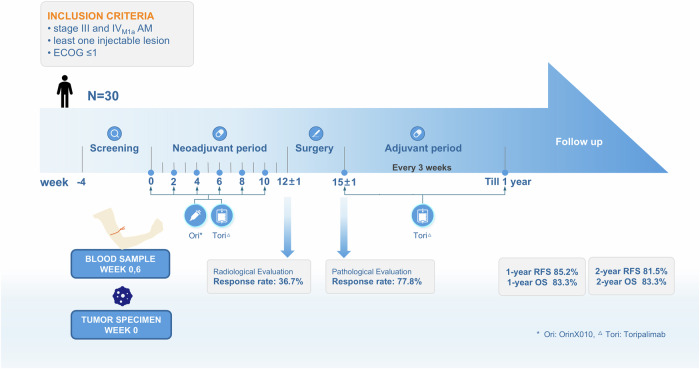
Comprehensive study flow and outcome summary

In this phase Ib, open label trial, which included patients with resectable stage III and IVM1a AM, ori combined with tori as neoadjuvant therapy followed by adjuvant tori showed 77.8% pathological response and 33.3% major pathological response. The 1-year and 2-year RFS rates from operation to recurrence were 85.2% and 81.5%, respectively (Fig. 4), which were much higher than the previously reported RFS rates for adjuvant therapy alone.^27^ The median RFS was not reached, while the mean RFS reached 40.3 months.

Randomized clinical trials have been limited in adjuvant or neoadjuvant settings for AM. In the S1801 phase 2 trial, only nine AM patients were enrolled, and no separate data were provided for them.^15^ There is only one neoadjuvant trial on AM with anti-PD-1 monotherapy (NCT04248387), but no data has been available since Jan 2020. Most data on adjuvant therapy for AM are from retrospective studies, and all show limited efficacy of adjuvant therapy in the AM subtype, RFS, from 14.8 months to 26 months.^28^ Currently, the largest retrospective AM anti-PD-1 adjuvant therapy study showed a significant decrease in RFS for AM compared to stage III-IV cutaneous melanoma (14.8 months vs 37.4 months, *p* = 0.002).^29^ A randomized phase II study explored high-dose interferon α-2b as an adjuvant therapy for AM. The median RFS of the 1-year regimen was 22.5 months, and the 2-year RFS rate was 44.4%,^3^ which was almost half of that in our trial. However, it remains uncertain whether adjuvant interferon and anti-PD-1 therapy have equivalent efficacy in AM. A retrospective cohort analysis of Chinese patients who received adjuvant PD-1 inhibitors compared with standard high-dose interferon α-2b (HDI) did not find a difference in RFS among patients with AM (median, 7.0 months vs. 15.3 months), but the safety profile of PD-1 inhibitors is better than that of HDI.^30^ Based on the retrospective study, neither treatment appears to be optimal.

The groundbreaking NADINA study published in 2024 is a milestone in the development of neoadjuvant immunotherapy, being the first to validate this strategy in melanoma through a multicenter, international, randomized phase III clinical trial. The NADINA study demonstrated that neoadjuvant immunotherapy of ipilimumab and nivolumab significantly improved the EFS of patients compared with the traditional adjuvant nivolumab treatment (the 12-month EFS rates were 83.7% and 57.2%, respectively),^18^ may redefining the treatment paradigm for melanoma. The individualized treatment that considers different pathological responses simultaneously along with genetic variations is one of the highlights of the NADINA study, which introduced a regimen for patients with a pathological partial response (pPR) or non-response: when the BRAF mutation is present, dabrafenib in combination with trametinib was applied as adjuvant therapy, otherwise, nivolumab was administered. NADINA study exhibited a marked superiority of the efficacy of neoadjuvant therapy to adjuvant therapy, reconfirming neoadjuvant therapy as a burgeoning trend in the future.

AM is less responsive to immunotherapy than non-acral cutaneous melanoma. The data from five clinical trials of anti-PD-1 monotherapy in Asia showed the overall response rate was only 18.0% and median PFS was 3–5 months.^8^ Compared with cutaneous melanoma, AM samples display a significantly severe immunosuppressive state, including depletion of cytotoxic CD8+ T cells, enrichment of Treg cells, and exhausted CD8+ T cells.^4^ Anti-PD-1 monotherapy demonstrates limited efficacy in AM, and combination therapy of ICIs has also shown similarly modest results. A study employed the combination of CTLA-4 inhibitor (KD6001) and tori for advanced melanoma patients, including 9 (31.0%) of AM. Among the 13 evaluable patients without brain metastases, the unconfirmed ORR stood at 38.5%, whereas the confirmed ORR was 23.1%.^31^ Another study also deployed CTLA-4 inhibitor (IBI310) in combination with PD-1 inhibitor (sintilimab) for the treatment of advanced melanoma/urothelial carcinoma, enrolling 8 (23.5%, Phase 1b) AM patients, and reported an ORR of 17.6%.^32^ Given the low efficacy of anti-PD-1 monotherapy or anti-PD-1 plus CTLA-4 in AM, we selected a treatment regimen combination of anti-PD-1 with oncolytic virus for this neoadjuvant study, which showed a 77.8% pathological response rate. Even in comparison with the NADINA study, and despite being a small Phase I trial, the efficacy of this combination in the neoadjuvant treatment of AM is remarkable. We are encouraged by the potential of anti-PD-1 plus oncolytic virus therapy in advanced AM.

An oncolytic virus is able to modify the tumor microenvironment to make it more susceptible to concurrent ICI.^23^ There was evidence that T-VEC intratumoral administration produced a systemic increase in circulating CD4+ and CD8+ T cells and increased CD8+ T cell infiltration into tumors. Injection of the oncolytic virus T-VEC can change the tumor microenvironment by attracting T cells that may induce a systemic response to distant metastases after subsequent blockade of PD-1 with pembrolizumab.^23^ Cytokines and chemokines analysis revealed that the combination therapy of ori+tori significantly increased the secretion of cytokines critical for tumor elimination through various mechanisms. The elevated cytokines include those involved in direct tumor cell killing (TNF, TNFR, IFN-γ, Fas ligand); granzymes secreted immune cells (GZMA, GZMH, and GZMB), chemokines that promote cell recruitment and activation (CXCL9, CXCL10, CXCL11, CXCL13, CCL19, and CCL23); and interleukins regulating immune cell function (IL-12, IL-18, and IL-10). This extensive elevation in cytokines and chemokines suggests that virotherapy enhances immune cell activity, thereby improving sensitivity to immunotherapy.^33^ To date, oncolytic virus in combination with ICI has only been evaluated in clinical trials with patients with unresectable stage IIIB IV M1c melanoma.^23,34,35^ The combination of oncolytic virus with ICI in the phase III trial of T-VEC combined with pembrolizumab for advanced melanoma showed that this combination did not significantly improve PFS or OS compared with placebo-pembrolizumab,^36^ which may be due to different immune-microenvironments in cutaneous melanoma and AM. The immune reversal effect of oncolytic viruses in AM may not alter the prognosis of highly immune-sensitive cutaneous melanoma. So, in combination with anti-PD-1 therapy, intratumoral injection of ori engineered to enhance immune recognition of cancer resulted in a high response rate in patients with AM, especially after neoadjuvant treatment, which has the ability to elicit a broader tumor-specific T cell response.^12^ Although T-VEC has demonstrated its efficacy in the neoadjuvant treatment of melanoma with a 2-year RFS rate of 29.5%,^24^ our study confirmed the enhanced potential with a 2-year RFS rate of 81.5% for AM. The preliminary results revealed that compared to oncolytic virus neoadjuvant monotherapy, neoadjuvant therapy of ori+tori offered significant advantages in AM.

Pathologic response is a critical indicator for assessing the efficacy of neoadjuvant therapy in melanoma patients and is closely related to outcomes. Neoadjuvant treatment with relatlimab and nivolumab has demonstrated a similar trend of 2-year RFS rates (92% vs. 55% for those with or without pathologic response).^37^ Among patients treated with T-VEC plus surgery, 17.1% achieved a pCR, corresponding to a 2-year RFS rate of 75.5%, compared to 63.6% for those without pCR.^24^ Based on a comparison between the pathological response to ICIs and the pCR to T-VEC, the RFS rate remains numerically superior for ICIs. The reasons may be that T-VEC principally targets the localized tumor by intralesional administration,^24^ while ICIs lead to more widespread immune activation by inhibiting immune checkpoints systemically. Therefore, we utilized an oncolytic virus in combination with ICI, and achieved a superior efficacy to T-VEC monotherapy, along with a high correlation between pathological response and RFS.

Although neoadjuvant therapy has achieved creditable results, the question of whether to apply adjuvant therapy for patients who have achieved a major pathology response in the context of neoadjuvant therapy raises debate. PRADO trial demonstrated that patients with a major pathological response to neoadjuvant ipilimumab and nivolumab could forgo subsequent surgery and adjuvant therapy, as the plan would mitigate the AEs of adjuvant therapy and improve the quality of life for patients.^38^ The high pCR rate, the absence of recurrence, and good safety profiles confirmed that neoadjuvant therapy may be sufficient in certain cases.^37^ Furthermore, the PRADO trial showed that therapeutic lymph node dissection could be a treatment option for patients with pathologic non-response (pNR), followed by adjuvant therapy.^38^ In the NADINA trial, 59.0% of patients in the neoadjuvant treatment group achieved a major pathological response, thereby eliminating the adjuvant therapy.^18^ Nevertheless, the generalizability of findings from melanoma trials must be cautiously considered in the context of AM, since the immunosuppression AM exhibiting would lead to different responses to immunotherapies. From our trial, since most grade 3 AEs were associated with the adjuvant period, and patient outcomes were satisfactory after neoadjuvant treatment, we also tend to hold an opinion that sequent adjuvant therapy may not be necessary for patients with AM when major pathological response presented, due to the limited benefit of adjuvant therapy and the incurring increased risk of AEs. In future neoadjuvant trials for AM, the subsequent treatment and surgery with different pathologic response patterns still need to be explored.

In our trial, the majority of TEAEs were grade 1/2. Only 13.3% of patients had grade 3 AEs. The incidence of grade 3 or 4 AEs was similar to that of previous studies combining anti-PD-1 inhibitors and oncolytic virus.^24,39,40^ In comparison to NADINA, AEs of ori + tori were conspicuously lower in our trial. The grade 3 or higher in NADINA reached 47.2% in the neoadjuvant group.^18^ Among the grade 3 AEs, two patients experienced soft tissue infections after lymph node dissection. Wound infection complications were observed in 7.4% of cases, and this proportion did not increase after neoadjuvant treatment.^41^

Noteworthy, our trial yielded a pathological response rate of 77.8%, which was significantly higher than the radiographic overall response rate of 36.6%. Despite the increasing role of 18 F-FDG PET/CT in the post-treatment monitoring and staging of high-risk melanoma patients,[39] its applicability in our neoadjuvant trial appears to be limited. The evaluation showed no correlation between PET parameters and pathological response, which is consistent with existing studies.^34^ This underscores the necessity for new technologies to evaluate the efficacy of neoadjuvant therapy for advanced melanoma, particularly in Ori and tori combination therapy.

We acknowledge that the study is limited by its small sample size and that these results are preliminary based on findings at a single center. Our median follow-up went up to 24 months, and we acknowledge that additional follow-up is needed to fully assess the clinical impact and the durability of responses. However, these preliminary data are encouraging and require further confirmation in a controlled trial. The NADINA trial has illustrated the potentiated outcomes conferred by the combined application of a PD-1 inhibitor and a CTLA-4 inhibitor in the neoadjuvant setting.^18^ In addition, the PIVOTAL study substantiated that daromun, as a neoadjuvant agent in the localized treatment for patients afflicted with locally resectable stage III melanoma, intensified the immunological response within the tumor microenvironment.^42^ Synergistic neoadjuvant therapeutics of oncolytic viruses with PD-1 inhibitors have garnered evidence supporting their efficacy and safety.^26^ The confluence of PD-1 inhibitors, CTLA-4 inhibitors, and oncolytic virotherapy may herald a more promising therapeutic horizon for individuals with AM. Moreover, it is necessary to compare this approach head-to-head against combined neoadjuvant ICI for AM. In a future study, conduction of a 3-arm, randomized clinical trial (neoadjuvant therapy of PD-1 inhibitor + CTLA-4 inhibitor + oncolytic virus vs. neoadjuvant therapy of PD-1 inhibitor + CTLA-4 inhibitor vs. neoadjuvant therapy of oncolytic virus + PD-1 inhibitor) is necessary, with a crossover design to determine the role of each agent in this combination.

In summary, neoadjuvant oncolytic virus ori and anti-PD-1 tori is a highly active regimen that achieves a 77.8% pathological response rate with a favorable safety profile in patients with high-risk, resectable high-risk AM. A phase 3 randomized controlled study is still needed considering this is a single arm study. These data complement future studies in patients with unresectable metastatic AM, and further support the promise of this new combination immunotherapy regimen in this disease.

## Materials and methods

### Study design

This single-arm, open-label, single-center, phase Ib clinical trial was conducted at the Melanoma and Sarcoma Department of Peking University Cancer Hospital and Institute in Beijing, China (Clinical trial number: NCT04197882). The trial was approved by the Peking University Cancer Hospital and Research Institute’s ethics committee (2019YJZ35). The aim was to assess the efficacy and safety of combination therapy with ori and tori as neoadjuvant treatment for AM patients. Primary endpoints were radiographic and pathological response rates according to RECIST, version 1.1. Secondary endpoints included rates of 1-year and 2-year RFS (duration from the surgical procedure to recurrence specifically among patients who underwent surgery), rates of EFS (time from treatment initiation to recurrence in all patients), OS (measured from randomization to the date of death from any cause), and safety.

### Patients

Eligible patients were aged 18–75 years with histologically and/or cytologically confirmed, resectable, American Joint Committee on Cancer (AJCC) 8th Edition stage III and IVM1a AM. Additionally, patients were required to have at least one measurable lesion according to RECIST 1.1 and be candidates for intralesional therapy involving injectable nodal, subcutaneous, or cutaneous melanoma lesions (≥10 mm in longest diameter); Eastern Cooperative Oncology Group performance status ≤1, and adequate organ function were also required. All enrolled patients provided written informed consent.

### Study drug

Ori is designed to be injected directly into melanoma tumors.^25^ Notably, ori was modified on three HSV-1 virus-infected cell protein (ICP) genes (ICP6, ICP47, ICP34.5) and was engineered to express the human GM-CSF gene. ICP34.5 typically inhibits a cell-mediated antiviral response and promotes productive infection of healthy cells and neurons.^43–45^ By removing ICP34.5, ori replication is enhanced in cancer cells while preserving healthy cells.^43^ ICP47 curbs the immune destruction of HSV-1-infected cells,^44,46^ and its loss promotes antigen presentation and T cell activation, enhancing the immune system’s ability to identify tumor cells, and thus enhancing ori’s efficacy.^47^ The insertion of an inactivated ICP6 gene into the ori genome reduced the likelihood of post-injection neurotoxicity by inhibiting the growth of oncolytic HSV-1 virus in neurons.^43^ In addition, the GM-CSF gene was integrated into the original ICP34.5 site, promoting dendritic cell accumulation at the inflammatory site and enhancing antigen-presenting cell function.^44,48^

### Drug administration

Neoadjuvant treatment period (Fig. 1): Ori combined with tori injection. Tori injection: 3 mg/kg, IV infusion, once every 2 weeks for six doses; Ori: maximum dose of 8 × 10^8^ pfu up to 10 mL, intratumoral injection, once every 2 weeks for six doses.

Surgical treatment period: 2 weeks after the last dose of neoadjuvant treatment (±7 days).

Adjuvant treatment period: tori injection was given to patients 3 weeks (±7 days) after surgery. Tori injection: 3 mg/kg given intravenously every 3 weeks for up to 1 year (the 1-year duration was counted from the 1st dose of neoadjuvant treatment). If neoplasm recurrence, metastasis, or intolerable adverse events occurred or patients withdrew informed consent during the treatment, or there were other situations meeting the criteria for end of treatment, the study drug treatment would be terminated.

### Assessment

Prior to each treatment cycle, physical examination and laboratory analyses were performed for each patient. Additionally, photographs of cutaneous lesions were taken. At baseline, tumor specimens were collected before the first administration of ori + tori and during surgery to assess the pathological response (including Ki-67 expression, hyaline fibrosis, tertiary lymphoid structures [TLSs], and tumor-infiltrating lymphocytes [TILs]).

Tumor imaging (CT/MRI and PET/CT) was performed during the screening period and in the 12th week of neoadjuvant treatment to evaluate the anti-tumor effect. Tumor imaging follow-up was performed in subjects every 3 months in the postoperative adjuvant treatment period and every 4 months in the 2nd year, then every 6 months from the 3rd year to the 5th year.

Pathological response criteria followed the International Neoadjuvant Melanoma Consortium scoring system: pPR (10% ≤ active tumor cells ≤ 50%); near pathological complete response (Near pCR; 0% < active tumor cells < 10%); pathological complete response (pCR, no active tumor cells). Pathological response included pPR, near pCR, and pCR. pNR (>50% active tumor cells).

Cytokines and chemokines in 32 serum samples from 16 patients, collected at baseline and after 6 weeks of neoadjuvant treatment (C3), were measured using the proximity extension assays developed by Olink Proteomics (Sweden) and analyzed by Panovue (Beijing, China). Standard protocols for quality control and data normalization by referencing internal and external controls were carried out in the Olink normalized protein expression (NPX) Manager software (V.3.3.2.434).

### Safety evaluation

To assess potential adverse effects, a comprehensive evaluation was performed in patients who received at least one cycle of ori + tori. This assessment adhered to the NCI-CTCAE v. 5.0 guidelines. This monitoring began from the informed consent obtained 100 days after the end of the last treatment. Treatment-related and unrelated AEs were monitored.

### Statistical analysis

We hypothesized that neoadjuvant combination therapy with ori and tori in resectable stage IIIB/C/D-IVM1a AM patients would significantly increase the pathological response rate. If the pathological response rate was significantly higher than the 20% response rate for historical neoadjuvant ICI monotherapy, the combination treatment would be considered successful. In this study, we expected a pathological response rate of 45% when ori was combined with tori. To rigorously evaluate this expectation, we applied a precise binomial test with a nominal two-sided significance level of 10%. This statistical approach was expected to yield an impressive statistical power of 86.5%, enabling us to confidently determine the discernible difference between the null hypothesis proportion (*π*0) of 0.2 and the alternative proportion (*π*1) of 0.45, especially considering the sample size of 30 participants in the study.

For continuous variables, descriptive statistics included count, mean, standard deviation, median, maximum and minimum. For categorical variables, descriptive statistics included frequency, absolute rate or relative rate. Statistical analyses were performed using SAS® v9.3 or above.

## Supplementary information

Supplementary_Materials

Clinical Study Protocol

## Data Availability

The original contributions presented in this study are included in the article/supplementary files. Further inquiries can be directed to the corresponding author.
