# Supplementary material for: Neoadjuvant oncolytic virus orienx010 and toripalimab in resectable acral melanoma: a phase Ib trial
Source: Signal Transduct Target Ther. 2024 Nov 22;9:318. doi: 10.1038/s41392-024-02029-2 (PMC11582582; doi:10.1038/s41392-024-02029-2)
Supplement: Supplementary file 1 — Supplementary_Materials [file 41392_2024_2029_MOESM1_ESM.docx]

Supplementary Materials for

Neoadjuvant oncolytic virus orienx010 and toripalimab in resectable acral melanoma: a phase Ib trial

Running title: Orienx010 and toripalimab in acra melanoma

Jiayong Liu^1#^, Xuan Wang^2#^, Zhongwu Li^3#^, Shunyu Gao^4#^, Lili Mao^2^, Jie Dai^2^, Caili Li^2^, Chuanliang Cui^5^, Zhihong Chi^2^, Xinan Sheng^5^, Yumei Lai^3^, Zhichao Tan^1^, Bin Lian^2^, Bixia Tang^2^, Xieqiao Yan^5^, Siming Li^2^, Li Zhou^5^, Xiaoting Wei^2^, Juan Li^5^, Jun Guo^2^, Lu Si^2*,🖂^

Correspondence to: silu15_silu@126.com (Lu Si)

**This PDF file includes:**

Materials and Methods

Supplementary Figures S1 to S2

Supplementary Tables S1 to S2

**Materials and methods**

**Olink Proteomics**

Cytokines and chemokines in 32 serum samples from 16 patients, collected at baseline and after 6 weeks of neoadjuvant treatment (C3), were measured using the proximity extension assays developed by Olink Proteomics (Sweden).

Incubation

Prepare an Incubation mix according to the table below.

| **Incubation Mix** | **Volume Required per 96-well Plate (μL)** |
| --- | --- |
| Incubation Solution | 280 |
| Incubation Stabilizer | 40 |
| A-probes | 40 |
| B-probes | 40 |

Vortex and spin down the Incubation mix. Transfer 47 μL of the Incubation mix to each well of an 8-well strip.Transfer 3 μL of Incubation mix to each well of a 96-well plate by reverse pipetting and name it *Incubation Plate*. Add 1 μL of each sample to the *Incubation Plate,* using a multi-channel pipette, to the bottom of the well. In column 12, add 1 μL of Negative Control to three wells (red), and 1 μL of Interplate Control to three wells (green), according to the plate layout below. It is recommended to also run a pooled plasma sample as Sample Control (yellow) in two wells.


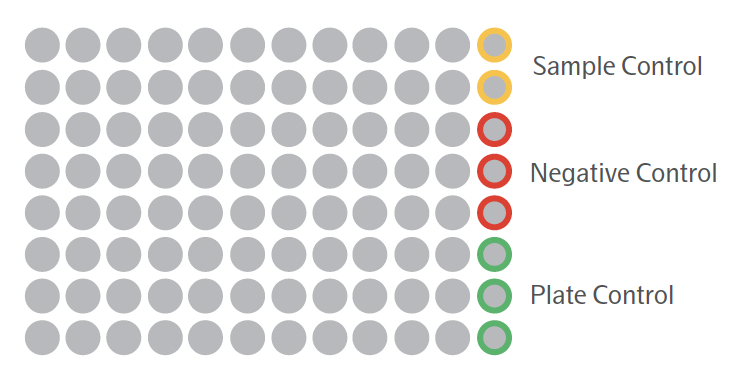


Seal the plate with an adhesive plastic film, spin at 400 – 1000 x g, 1 min at room temperature. Incubate overnight at +4 °C.

Extension

Prepare an Extension mix according to the table below.

| **Extension Mix** | **Volume Required per 96-well Plate (μL)** |
| --- | --- |
| High Purity Water | 9385 |
| PEA Solution | 1100 |
| PEA Enzyme | 55 |
| PCR Polymerase | 22 |

Bring the Incubation Plate to room temperature, spin at 400 – 1000 x g for 1 min. Preheat the PCR machine. Vortex the Extension mix and pour into a multichannel pipette reservoir. Time sensitive step: Start a timer for 5 min and transfer 96 μL of Extension mix to the upper parts of the well walls of the Incubation Plate, by using reverse pipetting.

Olink® Target 96 If running an Olink panel that requires pre-diluted samples, read section 7 Dilution step,

in the Olink® Target 96 User Manual prior to starting.Seal the plate with an adhesive plastic film, use MixMate® to vortex thoroughly at 2000 rpm for 30 sec, ensuring that all wells are mixed, and spin down at 400 – 1000 x g for 1 min Place the Incubation Plate in the thermal cycler, and start the PEA program. (50 °C 20 min, 95 °C 5 min (95 °C 30s, 54 °C 1 min, 60 °C 1 min) x 17, 10 °C hold)

Detection

Prepare and prime an Olink® 96.96 IFC for Protein Expression. Briefly, inject one control line fluid syringe into each accumulator on the chip, and then prime the IFC in the Olink Signature Q100 instrument. Thaw the *Primer Plate*, vortex and spin briefly. Prepare a Detection mix according to the table below.

| **Detection Mix** | **Volume Required per 96-well Plate (μL)** |
| --- | --- |
| Detection Solution | 550 |
| High Purity Water | 230 |
| Detection Enzyme | 7.8 |
| PCR Polymerase | 3.1 |

Vortex the Detection mix, spin briefly and add 95 μL to each well of an 8-well strip.

Transfer 7.2 μL of the Detection mix to each well of a new 96-well plate by reverse pipetting and name it *Sample Plate*. Remove the *Incubation Plate* from the thermal cycler, spin down the content and transfer 2.8 μL from each well to the corresponding well on the *Sample Plate*, using forward pipetting. Seal the plate with an adhesive plastic film, vortex and spin at 400 – 1000 x g, 1 min at room temperature. Transfer 5 μL from each well of the *Primer Plate* and 5 μL of the *Sample Plate* into the primed 96.96 IFC left and right inlets, respectively. Use reverse pipetting and change tips after each primer or sample. Do not leave any inlets empty. Remove bubbles and load the chip in the Olink Signature Q100 and follow the instructions on the instrument screen. Run the plate on the Olink Signature Q100 and make sure that the correct interface plate is used.

Data Collection Process

Run the SIGNATURE 100 QPCR instrument to collect data, and process the data through Olink NPX Signature software for QC analysis and NPX value calculation. The NPX software provides standardized protein expression values (Normalized Protein Expression) for further data analysis.

**Immunohistochemistry**

Four-micrometer sections were prepared from representative paraffin blocks containing invasive melanoma. Immunohistochemistry for Ki67 (clone MIB-1, DAKO M7240, 1:70 dilution) was performed followed by heat-mediated antigen retrieval in 0.01 M citrate buffer (pH 6.0) and detection using the DAKO Cytomation Envision/HRP kit (K5007). Only clear nuclear staining of invasive tumor cells was considered for scoring, with results expressed as the percentage of tumor cells positive for the marker.

**Supplementary Figures S1 to S2**


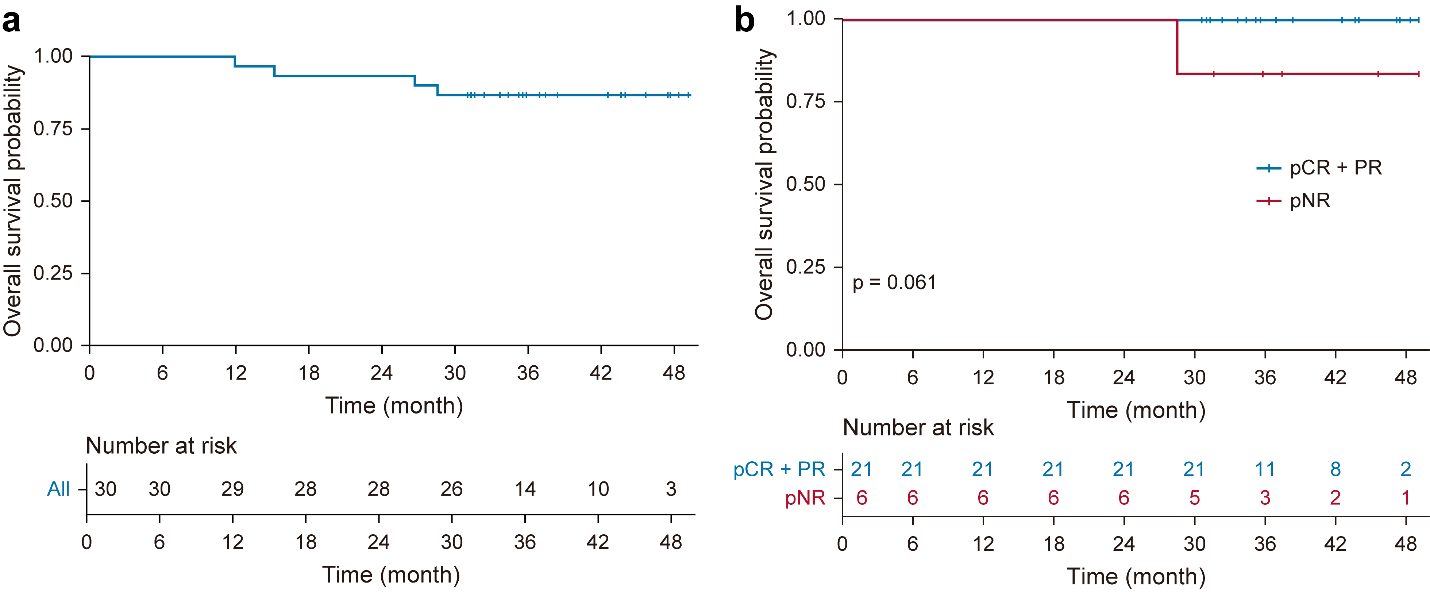


Figure. S1.

Overall survival curves. **a**: Overall survival curves; **b**: Probability of overall survival based on any pathologic response versus no pathologic response.


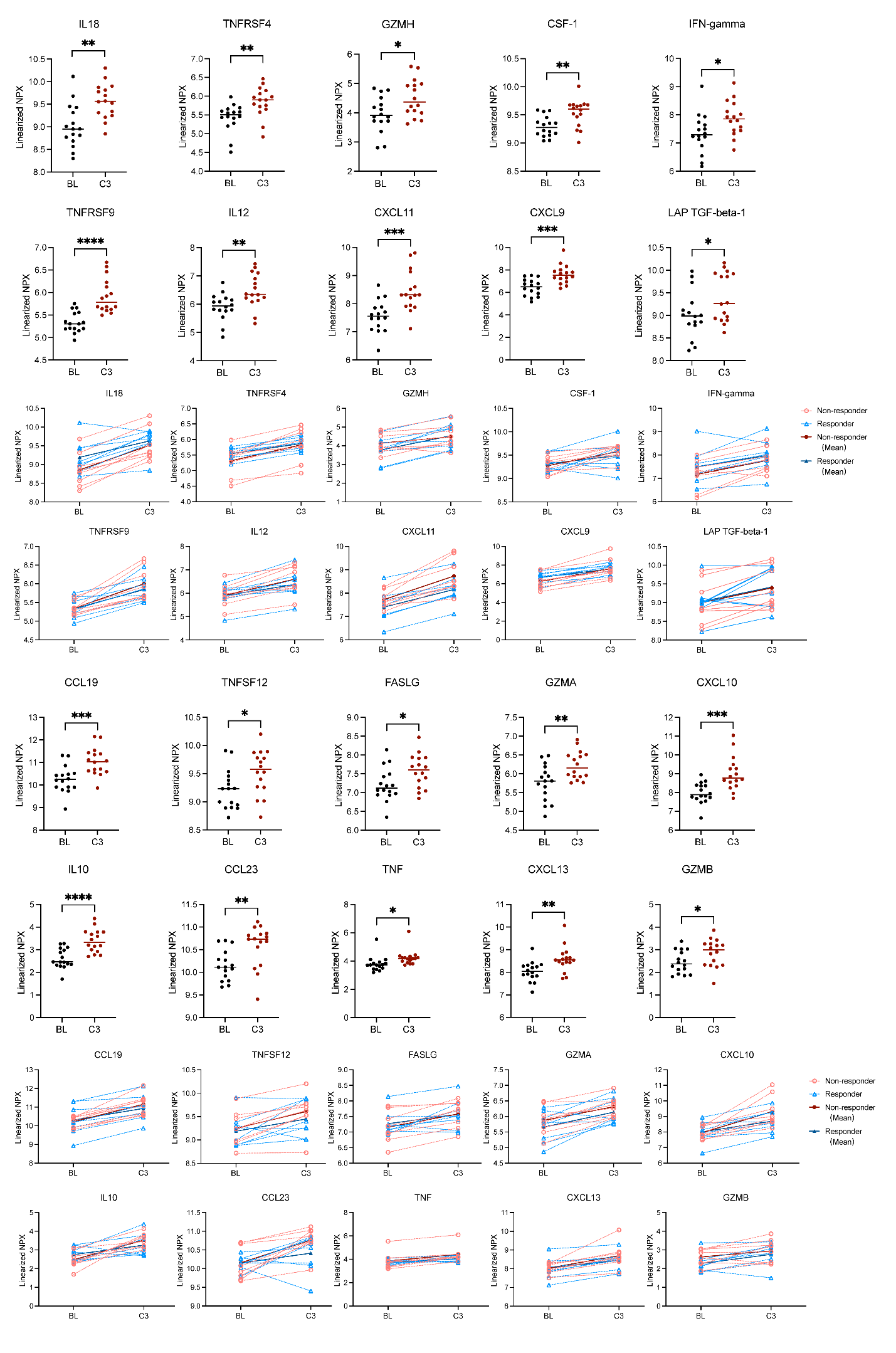


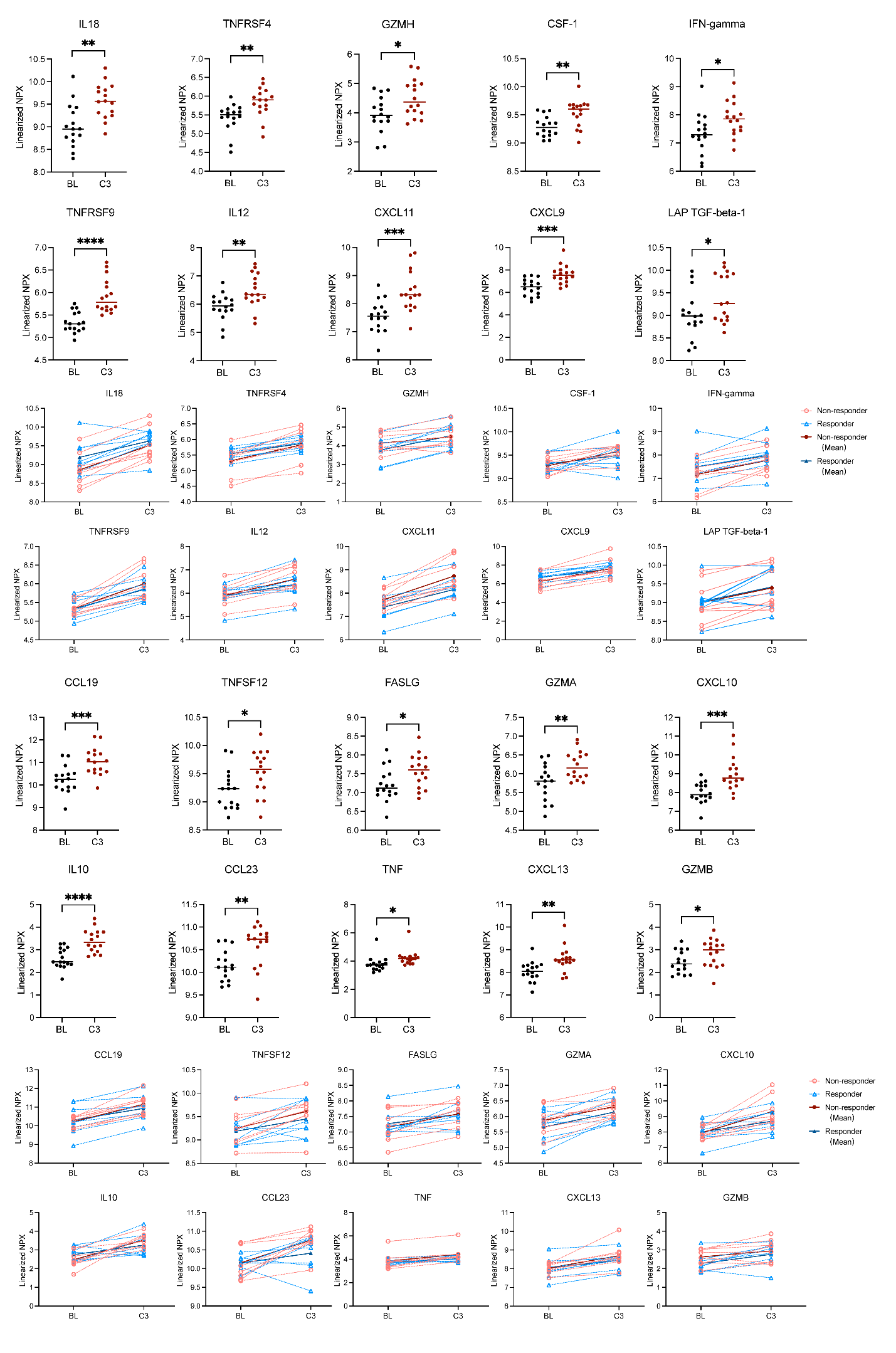


Figure. S2.

Cytokines and chemokines analysis of 16 patients at treatment baseline (BL) and after neoadjuvant treatment (6 weeks:C3). Showed the combination of oncolytic virus and anti-PD-1 monoclonal antibody therapy can significantly increase the expression of cytokines in both groups (pCR+near pCR and pNR) of patients

**Supplementary Tables S1 to S2**

Table S1.

Radiographic response vs. pathologic response

| Patients | Radiographic response | Pathologic response |
| --- | --- | --- |
| S01 | PD | pNR |
| S03 | PR | pPR |
| S05 | PD | pPR |
| S06 | PR | pPR |
| S07 | PD | pPR |
| S09 | PD | pPR |
| S10 | PD | NA |
| S11 | PR | pCR |
| S12 | SD | pPR |
| S13 | SD | pPR |
| S16 | PR | NA |
| S17 | PR | pPR |
| S20 | PD | pNR |
| S21 | SD | pNR |
| S22 | SD | pPR |
| S23 | SD | pPR |
| S25 | PD | NA |
| S26 | SD | pNR |
| S27 | SD | pPR |
| S28 | PR | Major PR/Near pCR |
| S30 | CR | pCR |
| S32 | SD | Major PR/Near pCR |
| S34 | PR | Major PR/Near pCR |
| S35 | SD | pNR |
| S36 | PR | pCR |
| S37 | PR | Major PR/Near pCR |
| S38 | PR | pCR |
| S42 | SD | pPR |
| S43 | SD | pPR |

NA: patients did not proceed to surgery due to the development of distant metastatic disease.

Table S2.

Tumor bed identified by pathologic response.

| Tumor bed |  | pNR | pCR+pPR | P |
| --- | --- | --- | --- | --- |
| TIL | High | 3/6 | 20/21 | p=0.006 |
| Hyaline fibrosis^a^ | Yes | 0/6 | 8/19 | p=0.054 |
| TLS | ≥3/LPF | 1/6 | 12/17 | p=0.022 |
| Ki67%^b^ | Decrease | 0/3 | 3/10 | p=0.279 |

a: hayline fibrosis visible; b: Ki67% decrease after treatment
