# Supplementary material for: Neoadjuvant oncolytic virus orienx010 and toripalimab in resectable acral melanoma: a phase Ib trial
Source: Signal Transduct Target Ther. 2024 Nov 22;9:318. doi: 10.1038/s41392-024-02029-2 (PMC11582582; doi:10.1038/s41392-024-02029-2)
Supplement: Supplementary file 2 — Clinical Study Protocol [file 41392_2024_2029_MOESM2_ESM.pdf]

Clinical Study Protocol

**Protocol Title:**	**An Open-label Phase 1b Clinical Trial of Recombinant Human GM-CSF Herpes Simplex Virus Injection (OrienX010) in Combination with Recombinant Humanized Anti-PD-1 Monoclonal Antibody Injection (Toripalimab Injection) as Neoadjuvant Therapy of Completely Resectable Stage III and IV (M1a) Melanoma**
**Protocol No.:**	**OrienX010-II-12**
**Protocol Date:**	**August 11, 2020, V4.0**
**Study Drug:**	**Recombinant Human GM-CSF Herpes Simplex Virus Injection (OrienX010)**<br>**Recombinant Humanized Anti-PD-1 Monoclonal Antibody Injection (Toripalimab Injection)**
**Clinical Approval Letter No.:**	**2009 L11659**
**Funder:**	**OrienGene Biotechnology Ltd.**
**Sponsor:**	**Beijing Caner Hospital**
**Principal Investigator:**	**Jun Guo**
**Clinical Study Site:**	**Beijing Caner Hospital**
**GCP Statement**	**This study is conducted in compliance with *Good Clinical Practice* (GCP) and all records have been attached according to GCP requirements.**

Confidentiality Statement

This is a confidential document of OrienGene Biotechnology Ltd. (funder). Any organization or individual that receives this document shall not publish or disclose the information provided herein without prior written approval.

I Signature Page: Funder

I have read the protocol titled “An Open-label Phase 1b Clinical Trial of Recombinant Human GM-CSF Herpes Simplex Virus Injection (OrienX010) in Combination with Recombinant Humanized Anti-PD-1 Monoclonal Antibody Injection (Toripalimab Injection) as Neoadjuvant Therapy of Completely Resectable Stage III and IV (M1a) Melanoma”, protocol number: OrienX010-II-12. I agree to conduct this study in accordance with the study protocol, Good Clinical Practice (GCP), Declaration of Helsinki and local regulations, if applicable.

Funder-OrienGene Biotechnology Ltd.

Project Head: Zinan Xiao

Zinan Xiao	Medical Director
Name (Print)	Position
Signature	Date

II Signature Page: Investigator

I have read the protocol titled "An Open-label Phase 1b Clinical Trial of Recombinant Human GM-CSF Herpes Simplex Virus Injection (OrienX010) in Combination with Recombinant Humanized Anti-PD-1 Monoclonal Antibody Injection (Toripalimab Injection) as Neoadjuvant Therapy of Completely Resectable Stage III and IV (M1a) Melanoma", protocol number: OrienX010-II-12. I agree to conduct this study in accordance with the study protocol, Good Clinical Practice (GCP), Declaration of Helsinki and local regulations, if applicable and to provide guidance and assistance to other personnel of the site participating in this study.

Site Name: Beijing Caner Hospital

Principal Investigator:

Jun Guo	Principal Investigator
Name (Print)	Position
Signature	Date

III Protocol Synopsis

**Funder:** OrienGene Biotechnology Ltd.	**Protocol No.:** OrienX010-II-12
**Study drug:** Recombinant Human GM-CSF Herpes Simplex Virus Injection (OrienX010)<br>Recombinant Humanized Anti-PD-1 Monoclonal Antibody Injection (Toripalimab Injection)	**Study phase: phase 1**
**Study title:** An Open-label Phase 1b Clinical Trial of Recombinant Human GM-CSF Herpes Simplex Virus Injection (OrienX010) in Combination with Recombinant Humanized Anti-PD-1 Monoclonal Antibody Injection (Toripalimab Injection) as Neoadjuvant Therapy of Completely Resectable Stage III and IV (M1a) Melanoma	
**Study site:** Beijing Cancer Hospital	
**Planned duration of study:** expected from February 2019 to February 2021	
**Study population:** Patients with completely resectable stage III and IV (M1a) melanoma.	
**Planned number of subjects:** The study plans to enroll about 30 patients.	
**<u>Study objective:</u>** To evaluate the efficacy and safety of recombinant human GM-CSF herpes simplex virus injection (OrienX010) in combination with recombinant humanized anti-PD-1 monoclonal antibody (toripalimab injection) as neoadjuvant therapy of completely resectable stage III and IV (M1a) melanoma.<br>**<u>Primary study objective:</u>**<br>To evaluate pathological response rates (pPR, Major PR/Near pCR and pCR rates) and clinical response rate (based on RECIST1.1) with recombinant human GM-CSF herpes simplex virus injection (OrienX010) in combination with recombinant humanized anti-PD-1 monoclonal antibody (toripalimab injection) as neoadjuvant therapy of completely resectable stage III and IV (M1a) melanoma.<br>Pathological response rates will be determined by pathologist according to International Neoadjuvant Melanoma Consortium (INMC) scoring system.<br>**<u>Secondary study objectives:</u>**<br>1) To evaluate clinical response rate as per iRECSIT and iT-RECIST.<br>2) To evaluate 1-year recurrence-free survival (RFS) and 2-year RFS with OrienX010 combined with toripalimab injection as neoadjuvant therapy in patients with completely resectable stage III and IV (M1a) melanoma.<br>3) To evaluate 1-year event-free survival rates (EFS) and 2-year EFS with OrienX010 combined with toripalimab injection as neoadjuvant therapy in patients with completely resectable stage III and IV (M1a) melanoma.	

**Funder:** OrienGene Biotechnology Ltd.	**Protocol No.:** OrienX010-II-12
**Study drug:** Recombinant Human GM-CSF Herpes Simplex Virus Injection (OrienX010)<br>Recombinant Humanized Anti-PD-1 Monoclonal Antibody Injection (Toripalimab Injection)	**Study phase: phase 1**
**Study title:** An Open-label Phase 1b Clinical Trial of Recombinant Human GM-CSF Herpes Simplex Virus Injection (OrienX010) in Combination with Recombinant Humanized Anti-PD-1 Monoclonal Antibody Injection (Toripalimab Injection) as Neoadjuvant Therapy of Completely Resectable Stage III and IV (M1a) Melanoma	
4) To evaluate overall survival (OS) with OrienX010 combined with toripalimab injection as neoadjuvant therapy in patients with completely resectable stage III and IV (M1a) melanoma.<br>5) To evaluate the safety of OrienX010 combined with toripalimab injection as neoadjuvant therapy in patients with completely resectable stage III and IV (M1a) melanoma.<br>6) To observe surgery related events.	
**Study design and methods:**<br>This is an open-label phase 1b clinical trial of recombinant human GM-CSF herpes simplex virus injection (OrienX010) in combination with recombinant humanized Anti-PD-1 monoclonal antibody injection (toripalimab injection) as neoadjuvant therapy of completely resectable stage III and IV (M1a) melanoma.<br>The study plans to enroll about 30 patients with completely resectable stage III and IV (M1a) melanoma meeting protocol requirements in Beijing Cancer Hospital.<br>Patients will enter a 28-day (maximum) screening period after signing written informed consent form (ICF). In the screening period, investigators will evaluate patients' eligibility according to the protocol, including complete medical history, body height, body weight, vital signs, physical examination, ECOG performance status scores, electrocardiogram, laboratory related evaluation, computerised tomography (CT) or magnetic resonance imaging (MRI), and other tumor imaging evaluation.<br>The study includes 3 treatment periods, that is, neoadjuvant therapy, surgical therapy and adjuvant therapy:<br>Neoadjuvant treatment period: OrienX010 combined with toripalimab injection.<br>● Toripalimab injection: 3 mg/kg, IV infusion: Once every 2 weeks for 6 doses (every 2 weeks per cycle; 6 cycles);<br>● OrienX010: maximum injection dose 8×10^{8}pfu, intratumoral injection: Once every 2 weeks for 6 doses (every 2 weeks per cycle; 6 cycles);<br>Surgical treatment period: 2 weeks after the last dose of neoadjuvant treatment (± 7 days), the investigator will design the surgical protocol of the melanoma radical surgery according to the patient's individual disease, and perform postoperative care according to the patient's condition. Subjects' specimens will be collected during operation and the specimens will be processed according to relevant requirements. Pathological image reading personnel of the study site will carry out routine pathological examination and report, and evaluate pathological response.	

**Funder:** OrienGene Biotechnology Ltd.	**Protocol No.:** OrienX010-II-12
**Study drug:** Recombinant Human GM-CSF Herpes Simplex Virus Injection (OrienX010)<br>Recombinant Humanized Anti-PD-1 Monoclonal Antibody Injection (Toripalimab Injection)	**Study phase: phase 1**
**Study title:** An Open-label Phase 1b Clinical Trial of Recombinant Human GM-CSF Herpes Simplex Virus Injection (OrienX010) in Combination with Recombinant Humanized Anti-PD-1 Monoclonal Antibody Injection (Toripalimab Injection) as Neoadjuvant Therapy of Completely Resectable Stage III and IV (M1a) Melanoma	

Adjuvant treatment period: Toripalimab injection will be given to patients 3 weeks (± 7 days) after surgery.

- Toripalimab injection: 3 mg/kg intravenously given every 3 weeks (every 3 weeks per cycle) for up to 1 year (the one-year duration will be counted from 1st dose in neoadjuvant treatment). If neoplasm recurrence, metastasis or intolerable AE occurs or subjects withdraw informed consent during the treatment or there are other situations meeting the criteria for end of treatment (EOT), subjects need to terminate study drug treatment.

Safety follow-up will be continued until 90 days (± 7 days) after the last dose. See study diagram for specific visit plan.

<u>**Response evaluation criteria in tumors**</u>

The primary efficacy measures will be evaluated and documented based on pathological response rates and efficacy based on RECIST1.1.

Pathological response criteria: International Neoadjuvant Melanoma Consortium (INMC) scoring system.

Pathological partial response (pPR, active tumor cells ≤ 50％)

Near pathological complete response (Major PR/Near pCR, 0% < active tumor cells < 10%)

Pathological complete response (pCR, no active tumor cells)

RECIST1.1: Progressive disease (PD): At least a 20% increase in the sum of diameters of target lesions or the appearance of one or more new lesions; stable disease (SD): Neither sufficient shrinkage to qualify for PR nor sufficient increase to qualify for PD; partial response (PR): At least a 30% decrease in the sum of diameters of target lesions for at least 4 weeks; complete response (CR): Disappearance of all target lesions, no appearance of new lesions, and normal tumor markers for at least 4 weeks.

Tumor imaging examination will be performed in the screening period and at week 12 of neoadjuvant treatment period to evaluate anti-tumor efficacy of neoadjuvant therapy. Tumor imaging follow-up will be performed in subjects every 12 weeks in postoperative adjuvant treatment period. Tumor imaging examination methods include PET-CT, enhanced CT, MRI, ECT, and superficial lymph node B-mode ultrasound; if superficial lymph node B-mode ultrasound shows suspected lymph node metastases and it is unable to clarify clinically, aspiration biopsy can be performed. If necessary, lymphadenectomy or dissection can be performed to clarify whether there are metastases and the number of metastases. If subjects are allergic to contrast media, plain CT scan can be performed.

**Funder:** OrienGene Biotechnology Ltd.	**Protocol No.:** OrienX010-II-12
**Study drug:** Recombinant Human GM-CSF Herpes Simplex Virus Injection (OrienX010)<br>Recombinant Humanized Anti-PD-1 Monoclonal Antibody Injection (Toripalimab Injection)	**Study phase: phase 1**
**Study title:** An Open-label Phase 1b Clinical Trial of Recombinant Human GM-CSF Herpes Simplex Virus Injection (OrienX010) in Combination with Recombinant Humanized Anti-PD-1 Monoclonal Antibody Injection (Toripalimab Injection) as Neoadjuvant Therapy of Completely Resectable Stage III and IV (M1a) Melanoma	

Whole-body PET-CT and other necessary examinations according to the investigator's judgment will be performed in the screening period to prove subjects have no regional or distant metastases;

In the first year of postoperative toripalimab therapy, chest, abdomen and pelvic enhanced CT, superficial lymph node B-mode ultrasound will be performed once every 3 months. At the end of adjuvant treatment period, enhanced head CT and bone scan (additional examinations may be performed if clinically indicated) will be performed once; if subjects do not have neoplasm recurrence or metastasis at EOT, they need to continue tumor imaging follow-up, enhanced chest, abdomen and pelvic CT scan and superficial lymph node B-mode ultrasound once every 4 months in the second year upon postoperative toripalimab therapy, once every 6 months from the third year to the fifth year, and once every year afterwards, enhanced head CT and bone scan (additional examinations can be performed if clinically indicated) once every year until neoplasm recurrence, metastasis, initiation of a new anti-tumor therapy, withdrawal of informed consent, death or end of study, whichever occurs first. Subsequent tumor imaging examination method should be consistent with that in the screening period.

Follow-up of neoplasm recurrence

If a patient does not experience neoplasm recurrence during treatment withdrawal, he/she needs to receive subsequent neoplasm recurrence follow-up:

If a patient does not experience neoplasm recurrence during treatment withdrawal, he/she needs to receive subsequent follow-up for tumor assessment until neoplasm recurrence.

Survival follow-up

After PD or neoplasm recurrence, survival follow-up is still needed to collect subsequent anti-tumor therapy and survival information of patients:

Patients who experience PD or neoplasm recurrence will receive follow-up of survival status and subsequent anti-tumor therapy every 30 days (± 7 days) via outpatient visit or telephone until death, loss to follow-up, withdrawal of informed consent or end of study (EOS), whichever occurs first.

**Funder:** OrienGene Biotechnology Ltd.	**Protocol No.:** OrienX010-II-12
**Study drug:** Recombinant Human GM-CSF Herpes Simplex Virus Injection (OrienX010)<br>Recombinant Humanized Anti-PD-1 Monoclonal Antibody Injection (Toripalimab Injection)	**Study phase: phase 1**
**Study title:** An Open-label Phase 1b Clinical Trial of Recombinant Human GM-CSF Herpes Simplex Virus Injection (OrienX010) in Combination with Recombinant Humanized Anti-PD-1 Monoclonal Antibody Injection (Toripalimab Injection) as Neoadjuvant Therapy of Completely Resectable Stage III and IV (M1a) Melanoma	
**End of study (EOS):**<br>The primary endpoint measure will be analyzed at month 12 after the last patient is enrolled or when at least 24 cases of pathological response event have been observed (whichever occurs first). Survival data of patients will continue to be collected after analysis of pathological response. It is planned to end the whole study after survival of 80% of patients has been collected.<br>If some patients are still using the drugs after EOS, the funder and/or drug donating party will continue providing the drugs until patients' EOT/death/drug marketing. During continuation of study drug treatment, tumor progression evaluation, survival and drug-related SAEs will be collected as supplemental data.	
**Main inclusion/exclusion criteria**<br>**Main inclusion criteria:**<br>1. An ICF approved by the Ethic Committee will be voluntarily signed by the patient prior to initiating any screening or specific study procedures;<br>2. Male or female patients between 18 and 75 years of age;<br>3. Patients with definite diagnosis of completely resectable stage III and IV (M1a) melanoma based on histology and/or cytology, and at least one measurable lesion.<br>4. Patients with ECOG performance status of 0 or 1;<br>5. Expected survival> 4 months;<br>6. Patients have sufficient organ functions and should meet the following conditions at screening according to reference laboratory range:<br>● White blood cell count ≥ 3.0 × 10^9/L;<br>● Absolute neutrophil count ≥ 1.5 × 10^9/L;<br>● Platelet count ≥ 100 × 10^9/L;<br>● Hemoglobin ≥ 90 g/L;<br>● Serum albumin ≥ 2.5 g/dL;	

**Funder:** OrienGene Biotechnology Ltd.	**Protocol No.:** OrienX010-II-12
**Study drug:** Recombinant Human GM-CSF Herpes Simplex Virus Injection (OrienX010)<br>Recombinant Humanized Anti-PD-1 Monoclonal Antibody Injection (Toripalimab Injection)	**Study phase: phase 1**
**Study title:** An Open-label Phase 1b Clinical Trial of Recombinant Human GM-CSF Herpes Simplex Virus Injection (OrienX010) in Combination with Recombinant Humanized Anti-PD-1 Monoclonal Antibody Injection (Toripalimab Injection) as Neoadjuvant Therapy of Completely Resectable Stage III and IV (M1a) Melanoma	

- Liver function: Bilirubin total ≤ 1.5 × upper limit of normal (ULN); alanine aminotransferase (ALT) and aspartate aminotransferase (AST) < 2.5 × ULN;
- **Renal function:** Serum creatinine ≤ 1.5 × ULN or the 24-hour creatinine clearance ≥ 50 mL/min (Cockcroft and Gault formula);
- International normalized ratio (INR) ≤ 1.5, and, activated partial thromboplastin time (APTT) or partial thromboplastin time ≤ 1.5 × ULN;

7. Female patients with childbearing potential (including premature menopause, menopausal < 2 years and non-surgical sterilization), male patients, and partners of male patients must agree to use effective contraception during the study: Surgical sterilization, oral contraceptives, intrauterine devices, sexual abstinence or barrier contraceptive combination spermicides; All patients must continue contraception for 6 months after the last treatment.

Main exclusion criteria:

1. Patients previously treated with T-VEC or similar drug therapy; patients previously treated with anti-PD-1, anti-PD-L1, anti-PD-L2 drug therapy;
2. Patients with negative anti-herpes simplex virus type I (HSV-1) antibodies IgG and IgM;
3. The patient's lesion does not meet the requirement of the intratumoral injection volume or is not suitable for intratumoral injection;
4. Patients who received anti-herpes simplex virus therapy (such as acyclovir, ganciclovir, valaciclovir, vidarabine) within 4 weeks prior to the first dose of study treatment;
5. Patients who have received another anti-tumor monoclonal antibody (mAb) within 4 weeks prior to the first dose of study treatment or haven't recover (≤ Grade 1) from adverse events due to prior therapy (occurring earlier than 4 weeks);
6. Patients with a history of other (including unknown primary) malignancies within 5 years prior to the first dose of trial treatment. Note: Except for fully treated stage 1 or 2 basal/squamous cell carcinoma of the skin, superficial bladder cancer, or in situ cancer that is treated with potentially curative therapy;
7. Patients with known hypersensitivity to the study drug, its active ingredient, excipients;
8. Patient with HBsAg positive and HBV DNA copies > 1×10^3 copies/mL;

**Funder:** OrienGene Biotechnology Ltd.	**Protocol No.:** OrienX010-II-12
**Study drug:** Recombinant Human GM-CSF Herpes Simplex Virus Injection (OrienX010)<br>Recombinant Humanized Anti-PD-1 Monoclonal Antibody Injection (Toripalimab Injection)	**Study phase: phase 1**
**Study title:** An Open-label Phase 1b Clinical Trial of Recombinant Human GM-CSF Herpes Simplex Virus Injection (OrienX010) in Combination with Recombinant Humanized Anti-PD-1 Monoclonal Antibody Injection (Toripalimab Injection) as Neoadjuvant Therapy of Completely Resectable Stage III and IV (M1a) Melanoma	

9. Patients with positive hepatitis C virus (HCV) antibodies or human immunodeficiency virus (HIV) antibodies;
10. Patients with any unstable systemic disease, including but not limited to: Serious infection, uncontrolled diabetes mellitus, unstable angina, cerebrovascular accident or transient cerebral ischemia, myocardial infarction, congestive heart failure, and serious arrhythmia, liver, kidney, or metabolic disease requiring medical treatment;
11. Patients with active CNS metastases. Patients may participate in the study if their CNS is adequately treated and their neurological symptoms recover to levels less than or equal to Grade 1 (CTCAE) for at least 2 weeks before enrollment, with the exception of residual signs or symptoms associated with CNS therapy. In addition, patients must be those who do not use corticosteroids or who take stable doses of ≤ 10 mg prednisone/day (or equivalent dose) or who decrease to ≤ 10 mg prednisone/day;
12. Patients with autoimmune disease, received liver or other organs transplantation once before, active pulmonary tuberculosis; or patients received major surgical procedures, live vaccination, immunotherapy within 4 weeks prior to study initiation;
13. Tumor's macrovascular invasion in the iliac and femoral vessels;
14. The disease (e.g., mental illness, etc.) or condition (e.g., alcoholism or drug abuse, etc.) of the patient may increase the patient's risk of receiving trial medication or affect the patient's compliance with the study requirements, or may confuse the study results;
15. Within 30 days of screening, the patient had received any other study product or had participated in another interventional clinical trial;
16. Pregnant or lactating women or women who are preparing for pregnancy or lactation in the study period; men or women who are unwilling to take effective contraception;
17. Other situations unsuitable for enrollment according to the investigator’s judgment.

Study drug

Drug A: OrienX010

Produced by OrienGene Biotechnology Ltd. Strength: 1.0 mL/vial. Labeled amount: the titer is 8.0×10^{7} pfu/mL, and the number of particles is not more than 1×10^{11} VP/mL.

**Funder:** OrienGene Biotechnology Ltd.	**Protocol No.:** OrienX010-II-12
**Study drug:** Recombinant Human GM-CSF Herpes Simplex Virus Injection (OrienX010)<br>Recombinant Humanized Anti-PD-1 Monoclonal Antibody Injection (Toripalimab Injection)	**Study phase: phase 1**
**Study title:** An Open-label Phase 1b Clinical Trial of Recombinant Human GM-CSF Herpes Simplex Virus Injection (OrienX010) in Combination with Recombinant Humanized Anti-PD-1 Monoclonal Antibody Injection (Toripalimab Injection) as Neoadjuvant Therapy of Completely Resectable Stage III and IV (M1a) Melanoma	

Drug B: Toripalimab Injection (Tuoyi®)

Strengths: 240 mg/6 mL/vial, 80 mg/2 mL/vial; sterile injection; shelf life: 24 months or 36 months; date of production: subject to the date of production indicated on the product package; storage condition: protect from light and store at 2 - 8 °C.

Dosing schedule

Subjects will receive preoperative toripalimab injection (Tuoyi®) combined with OrienX010 after enrollment (neoadjuvant treatment period) and will continue toripalimab treatment for up to 1 year after operation (adjuvant treatment period) (the one-year duration will be counted from 1st dose in neoadjuvant treatment). Treatment regimen is as follows:

Drug A: OrienX010

- Preoperative (neoadjuvant treatment period): OrienX010 will be administered by intratumoral injection at a total dose not over 10 mL (8.0×10^7pfu/mL, 1 mL/vial, maximum of 10 vials) each time, once every 2 weeks (every 2 weeks per cycle) for 6 doses (6 cycles). The dose is selected according to the size of the tumor (long diameter): When the long diameter is ≥5 cm, the dose is 10 ml; when 2-5 cm, the dose is not less than 5 ml; when ＜2 cm, the dose is not less than 3 ml. Investigators should evaluate the size of injected lesions of patients within 24h before each injection to determine the injection dose of the study drug. Toripalimab injection will be given after OrienX010 injection (at an interval of more than 60 min).

Drug B: Toripalimab Injection (Tuoyi®)

- Preoperative (neoadjuvant treatment period): 3 mg/kg, IV infusion: Once every 2 weeks for 6 doses (every 2 weeks per cycle; 6 cycles);
- Postoperative (adjuvant treatment period): Subjects will intravenously continue toripalimab therapy at 3 mg/kg every 3 weeks (every 3 weeks per cycle) for up to 1 year after surgery (the one-year duration will be counted from 1st dose in neoadjuvant treatment).

It should be seriously examined before liquid preparation that the package of toripalimab injection is intact, and there is no solidification, turbidity and precipitation of the solution in bottle.

Under aseptic conditions, a disposable syringe is used to withdraw the corresponding volume of study drug into a 100 mL normal saline (0.9% sodium chloride solution) infusion bag, the mixed diluent is gently inverted for 3-5 times to avoid forceful shaking. After mixing, use an aseptic, low-pyrogen, low-protein adsorption in-line filter (0.2 or 0.22 μm) for intravenous infusion, and the infusion time is 60 ± 5 min. If the diluted solution cannot be used immediately after prepared, it can be stored at 2 - 8 °C or room temperature for 12 hours. It is suggested to use within 4 hours.

**Funder:** OrienGene Biotechnology Ltd.	**Protocol No.:** OrienX010-II-12
**Study drug:** Recombinant Human GM-CSF Herpes Simplex Virus Injection (OrienX010)<br>Recombinant Humanized Anti-PD-1 Monoclonal Antibody Injection (Toripalimab Injection)	**Study phase: phase 1**
**Study title:** An Open-label Phase 1b Clinical Trial of Recombinant Human GM-CSF Herpes Simplex Virus Injection (OrienX010) in Combination with Recombinant Humanized Anti-PD-1 Monoclonal Antibody Injection (Toripalimab Injection) as Neoadjuvant Therapy of Completely Resectable Stage III and IV (M1a) Melanoma	

If neoplasm recurrence, metastasis or intolerable AE occurs or subjects withdraw informed consent during the treatment period or there are other situations meeting the criteria for EOT, subjects need to terminate study drug treatment. Or else, patients will receive treatment according to study protocol until: CR (disappearance of all lesions, only OrienX010), clinical related progressive disease (PDr), unacceptable toxicity, loss to follow-up, death or other situations meeting the criteria for EOT, whichever occurs first.

PDr: Progressive disease related to deterioration of clinical conditions, and/or the investigator considers patients must change anti-tumor therapy in such situation. Patients can continue administration until PDr to allow the occurrence of "delayed anti-tumor immune response".

Criteria for End of Treatment (EOT)

The treatment will be discontinued permanently if patients meet any one of the following criteria:

- If the investigator considers that it is in patient's best interests to discontinue study drug due to SAEs or serious clinical laboratory abnormalities, the drug will be discontinued and the funder will be immediately notified;
- Patients need to use another study drug for the treatment of melanoma for any cause;
- PDr;
- Neoplasm recurrence;
- Intolerable toxic effects;
- The interval between two adjacent doses is over 6 weeks for any cause;
- Patients become pregnant or fail to take appropriate contraception (for patients of childbearing potential).

Additionally, if patients' condition is not improved and/or continues to deteriorate after starting study treatment, study treatment can be discontinued according to the investigator's judgment. In the event of PDr, the investigator should obtain the PD confirmed by tumor imaging. If the investigator considers a patient needs EOT due to clinical progression, the investigator should discuss with the funder's medical monitor before withdrawal.

**Funder:** OrienGene Biotechnology Ltd.	**Protocol No.:** OrienX010-II-12
**Study drug:** Recombinant Human GM-CSF Herpes Simplex Virus Injection (OrienX010)<br>Recombinant Humanized Anti-PD-1 Monoclonal Antibody Injection (Toripalimab Injection)	**Study phase: phase 1**
**Study title:** An Open-label Phase 1b Clinical Trial of Recombinant Human GM-CSF Herpes Simplex Virus Injection (OrienX010) in Combination with Recombinant Humanized Anti-PD-1 Monoclonal Antibody Injection (Toripalimab Injection) as Neoadjuvant Therapy of Completely Resectable Stage III and IV (M1a) Melanoma	

Study duration:

All patients will receive screening assessment during the screening period (within 28 days before the first dose of study drug).

As per the protocol, patients will receive combination therapy of OrienX010 and toripalimab injection before operation, and receive toripalimab injection for up to 1 year after surgery (the one-year duration will be counted from 1st dose in neoadjuvant treatment) until occurrence of PDr, unacceptable toxicity, loss to follow-up, death or meeting other EOT criteria, whichever occurs first;

On day 28 (± 7 days) after the last dose, patients should receive EOT follow-up. Safety follow-up will be performed until 90 days (± 7 days) after the last dose.

If a patient does not experience neoplasm recurrence at EOT, he/she needs to receive subsequent tumor assessment and follow-up until neoplasm recurrence. See neoplasm recurrence follow-up above for details.

After neoplasm recurrence, subsequent anti-tumor therapy information of the patient requires to be collected and his/her survival status will be followed up until death. The whole study will end after survival status of 80% of patients has been collected.

Study evaluation measures:

Efficacy measures:

Primary efficacy measures:

- Pathological response rates (pPR, Major PR/Near pCR and pCR rates)
- Clinical response rate (CR, PR and SD rates based on RECIST1.1)

Secondary efficacy measures:

- Clinical response rate (based on iRECIST and iT-RECIST)
- RFS
- EFS
- OS

The primary efficacy measures will be evaluated and documented based on pathological response rates and efficacy based on RECIST1.1.

Tumor imaging examination will be performed at week 12 of neoadjuvant treatment to evaluate anti-tumor efficacy of neoadjuvant therapy. Tumor imaging examination and follow-up will be performed in subjects after operation. Tumor imaging examination methods include PET-CT, enhanced CT, MRI, ECT, and superficial lymph node B-mode ultrasound; if superficial lymph node B-mode ultrasound shows suspected lymph node metastases and it is unable to clarify clinically, aspiration biopsy can be performed. If necessary, lymphadenectomy or dissection can be performed to clarify whether there are metastases and the number of metastases. If subjects are allergic to contrast media, plain CT scan can be performed.

<table>
<tr><td>Funder: OrienGene Biotechnology Ltd.</td><td>Protocol No.: OrienX010-II-12</td></tr>
<tr><td>Study drug: Recombinant Human GM-CSF Herpes Simplex Virus Injection (OrienX010)
Recombinant Humanized Anti-PD-1 Monoclonal Antibody Injection (Toripalimab Injection)</td><td>Study phase: phase 1</td></tr>
<tr><td colspan="2">Study title: An Open-label Phase 1b Clinical Trial of Recombinant Human GM-CSF Herpes Simplex Virus Injection (OrienX010) in Combination with Recombinant Humanized Anti-PD-1 Monoclonal Antibody Injection (Toripalimab Injection) as Neoadjuvant Therapy of Completely Resectable Stage III and IV (M1a) Melanoma</td></tr>
<tr><td colspan="2">In the first year of postoperative toripalimab therapy, chest, abdomen and pelvic enhanced CT, superficial lymph node B-mode ultrasound will be performed once every 3 months. At the end of adjuvant treatment period, enhanced head CT and bone scan (additional examinations may be performed if clinically indicated) will be performed once; if subjects do not have neoplasm recurrence or metastasis at EOT, they need to continue tumor imaging follow-up, enhanced chest, abdomen and pelvic CT scan and superficial lymph node B-mode ultrasound once every 4 months in the second year upon postoperative toripalimab therapy, once every 6 months from the third year to the fifth year, and once every year afterwards, enhanced head CT and bone scan (additional examinations can be performed if clinically indicated) once every year until neoplasm recurrence, metastasis, initiation of a new anti-tumor therapy, withdrawal of informed consent, death or end of study, whichever occurs first. Subsequent tumor imaging examination method should be consistent with that in the screening period.
Safety measures:
Vital signs
Physical examination
ECOG performance status
Laboratory tests (hematology, blood chemistry and urinalysis)
12-lead ECG
AEs, treatment emergent adverse events (TEAEs), SAEs and adverse events of special interest (AESIs)</td></tr>
<tr><td colspan="2">Statistical analysis method:
Statistical analysis is completed by statisticians, including:
Mainly the descriptive statistics due to the small number of subjects, and the results should be combined with professional analysis.
Statistics of the number of subjects enrolled, conditions of dropped out and removed subjects, demographics and other baseline characteristics and safety and efficacy analysis.</td></tr>
</table>

**Funder:** OrienGene Biotechnology Ltd.	**Protocol No.:** OrienX010-II-12
**Study drug:** Recombinant Human GM-CSF Herpes Simplex Virus Injection (OrienX010)<br>Recombinant Humanized Anti-PD-1 Monoclonal Antibody Injection (Toripalimab Injection)	**Study phase: phase 1**
**Study title:** An Open-label Phase 1b Clinical Trial of Recombinant Human GM-CSF Herpes Simplex Virus Injection (OrienX010) in Combination with Recombinant Humanized Anti-PD-1 Monoclonal Antibody Injection (Toripalimab Injection) as Neoadjuvant Therapy of Completely Resectable Stage III and IV (M1a) Melanoma	
Statistical analysis procedures shall be subject to statistical analysis plan.<br>**Selection of statistical analysis data:**<br>①Safety Set (SS): Among all enrolled patients, all the cases that have used the drug at least once are included in the safety set for safety analysis.<br>②Full Analysis Set (FAS): It is an ideal case set based on the principle of intention-to-treat as much as possible, obtained by excluding the fewest and unreasonable cases from all subjects.<br>③Per-protocol Set (PPS): Patients who complete the drug treatment according to the protocol without major deviation from the protocol, and complete all the evaluations constitute the PPS of this study. PPS includes at least the following criteria:<br>Meet the inclusion/ exclusion criteria specified in the trial protocol;<br>Complete all scheduled medications and visits;<br>No drugs or treatments that may affect the efficacy evaluation are used during the trial.<br>Safety analysis:<br>All AEs will be classified according to the Medical Dictionary for Regulatory Activities coding system and graded according to CTCAE 5.0. The number of cases and percentage of all TEAEs, grade 3 and above TEAEs, SAEs and study drug-related TEAEs, study drug-related SAEs, TEAEs leading to discontinuation of study drug, TEAEs and AESIs leading to termination of trial will be summarized by system organ class (SOC), preferred term (PT) and group. Moreover, the severity of TEAEs and their correlation with study drug will also be summarized by SOC, PT and group.<br>Observation values and changes from baseline in vital signs, physical examination, laboratory tests and 12-lead ECG will be analyzed using descriptive statistical method. Baseline results and the worst results in the trial will be presented with crosstab. ECOG baseline results and the worst results in the trial will be presented with crosstab.	

IV Schedule of Activities

Table1 Schedule of Activities

Stage	Screening period	Baseline	Neoadjuvant treatment period[a]	Surgical therapy[b]	Adjuvant treatment period[c]	After EOT		
			OrienX010 + toripalimab injection		Toripalimab injection	EOT visit[d]	Follow-up of neoplasm recurrence[e]	Survival follow-up[f]
Number of cycles (C) days (D)	D-28~ D-1	C1D1	CnD1	2 weeks after the last dose of neoadjuvant therapy	CnD1 (3 weeks (± 7 days) after surgery is C1D1 of the cycle)	Day 28 after the last dose	Once every 3 months	Telephone follow-up once every 30 days
Time window (days)	-	-	±3	±7	±3	±7	±28	±7
Informed Consent	X							
Evaluation of inclusion/exclusion criteria	X	X						
Demographic information	X							
Prior medical history[g]	X							
Prior medication	X							
Measuring height/weight[h]	X	X	X[l]		X[l]	X		
Physical examination	X	X	X[l]		X[l]	X	X	
Vital signs	X	X	X[l]		X[l]	X	X	
ECOG performance status	X	X	X[l]		X[l]	X	X	

Stage	Screening period	Baseline	Neoadjuvant treatment period[a]	Surgical therapy[b]	Adjuvant treatment period[c]	After EOT		
			OrienX010 + toripalimab injection		Toripalimab injection	EOT visit[d]	Follow-up of neoplasm recurrence[e]	Survival follow-up[f]
Number of cycles (C) days (D)	D-28~ D-1	C1D1	CnD1	2 weeks after the last dose of neoadjuvant therapy	CnD1 (3 weeks (± 7 days) after surgery is C1D1 of the cycle)	Day 28 after the last dose	Once every 3 months	Telephone follow-up once every 30 days
Time window (days)	-	-	±3	±7	±3	±7	±28	±7
score								
12-lead ECG[i]	X	X[i]				X		
Tumor TNM staging	X							
Hematology	X	X[j]	X[l]		X[l]	X	X	
Blood chemistry	X	X[j]	X[l]		X[l]	X	X	
Urinalysis	X	X[j]	X[l]		X[l]	X	X	
Blood coagulation function	X		X[k]			X		
Thyroid function	X		At week 12 of treatment		Every 9 weeks	X		
Blood pregnancy test[m]	X					X		
Virus serology test	X							
HSV-1 antibody test	X					X		
Tumor tissue biopsy[n]	X		X[n]					
Blood sample retention for	X		Every 6 weeks (±7d)		X			

Stage	Screening period	Baseline	Neoadjuvant treatment period[a]	Surgical therapy[b]	Adjuvant treatment period[c]	After EOT		
			OrienX010 + toripalimab injection		Toripalimab injection	EOT visit[d]	Follow-up of neoplasm recurrence[e]	Survival follow-up[f]
Number of cycles (C) days (D)	D-28~ D-1	C1D1	CnD1	2 weeks after the last dose of neoadjuvant therapy	CnD1 (3 weeks (± 7 days) after surgery is C1D1 of the cycle)	Day 28 after the last dose	Once every 3 months	Telephone follow-up once every 30 days
Time window (days)	-	-	±3	±7	±3	±7	±28	±7
biomarker test[o]								
Tumor imaging evaluation (CT or MRI)[p]	X		Week 12 (± 7d)		Every 12 weeks (±7d)	X	X	
Tumor-related clinical evaluation[q]			Every 6 weeks (±7d)					
Photography and calliper measurement of superficial lesions[r]		X	X[l]		X[l]	X	X	
Pre-dose evaluation[s]		X	X[l]		X[l]			
Cotton swab for wiping and sampling[t]			X[t]		X[t]	X[t]		
Collecting information of concomitant medications	X	X	X		X	X		
Collecting information of	X	X	X		X	X		

Stage	Screening period	Baseline	Neoadjuvant treatment period[a]	Surgical therapy[b]	Adjuvant treatment period[c]	After EOT		
			OrienX010 + toripalimab injection		Toripalimab injection	EOT visit[d]	Follow-up of neoplasm recurrence[e]	Survival follow-up[f]
**Number of cycles (C) days (D)**	**D-28~ D-1**	**C1D1**	**CnD1**	**2 weeks after the last dose of neoadjuvant therapy**	**CnD1 (3 weeks (± 7 days) after surgery is C1D1 of the cycle)**	**Day 28 after the last dose**	**Once every 3 months**	**Telephone follow-up once every 30 days**
**Time window (days)**	**-**	**-**	**±3**	**±7**	**±3**	**±7**	**±28**	**±7**
**AEs**								
**Subsequent anti-tumor therapy and survival status**							X	X

Abbreviations: AE = adverse event. BMI = body mass index. CR = complete response. CT = computerised tomography. ECOG = Eastern Cooperative Oncology Group. EOS = end of study. EOT = end of treatment. HSV-1 = anti-type I herpes simplex virus. iCPD = progressive disease confirmed based on iRECIST. iUPD = progressive disease unconfirmed based on iRECIST. MRI = magnetic resonance imaging. PD = progressive disease. PDr = clinical related progressive disease. SAE = serious adverse event.

a. Patients will continue treatment in the neoadjuvant treatment period until: CR (disappearance of all lesions, only OrienX010), PDr, unacceptable toxicity, loss to follow-up, death or situations meeting other EOT criteria, whichever occurs first. Neoadjuvant treatment period of OrienX010 + toripalimab injection: First inject OrienX010 at a total dose of not more than 10 mL/dose (8.0×10^7 pfu/mL, 1 mL/vial, maximum of 10 vials; the dose is determined by tumor size (long diameter)), followed by toripalimab injection at 3 mg/kg by intravenous infusion within 1 hour, once every 2 weeks for both drugs.

b. Surgical treatment period: 2 weeks after the last dose of neoadjuvant treatment (± 7 days), the investigator will design the surgical protocol of the melanoma radical surgery according to the patient's individual disease, and perform postoperative care according to the patient's condition. Subjects' specimens will be collected during operation and the specimens will be processed according to relevant requirements. Pathological image reading personnel of the study site will carry out routine pathological examination and report, and evaluate pathological response.

c. Adjuvant treatment period: Patients will start adjuvant therapy with toripalimab injection since 3 weeks (± 7 days) after operation for up to 1 year (the one-year duration will be counted from 1st dose in neoadjuvant treatment) until: the occurrence of PDr, unacceptable toxicity, loss to follow-up, death or meeting other EOT criteria, whichever occurs first; Toripalimab injection 3 mg/kg will be administered by intravenous infusion within 1 hour once every 3 weeks.

d. EOT visit: Patients need to receive EOT visit on day 28 (± 7 days) after the last dose. Patients who withdraw from the study prematurely will also require EOT visit. In addition, safety follow-up will be performed until 90 days (± 7 days) after the last dose. During safety follow-up period, investigators can collect all study drug-related AEs via outpatient or telephone visit.

e. Tumor recurrence follow-up: Patients who discontinue treatment for causes other than neoplasm recurrence will require to receive subsequent tumor assessment follow-up [once every 3 months (± 28 days) in the first year after starting postoperative treatment, as of the date of the last efficacy evaluation, once every 4 months (± 28 days) in the second year, once every 6 months (± 28 days) from the third year to the fifth year, afterwards, enhanced chest, abdomen and pelvis CT, superficial lymph node B-mode ultrasound once every year (± 28 days), enhanced head CT and bone scan (additional examinations can be performed if clinically indicated) once every year until neoplasm recurrence, metastasis, starting a new anti-tumor therapy, withdrawal of informed consent, death or EOS, whichever occurs first] if they do not have neoplasm recurrence at EOT.

f. Survival follow-up: Patients who experience PD or neoplasm recurrence will receive follow-up of survival status and subsequent anti-tumor therapy every 30 days (± 7 days) via outpatient visit or telephone until death, loss to follow-up, withdrawal of informed consent or EOS, whichever occurs first. The whole study plan will end after survival of 80% of patients has been collected. If some patients are still using OrienX010 and/or toripalimab injection after EOS, the funder and/or drug donating party will continue providing such drug until these patients cannot benefit from the treatment any more (at the discretion of investigator)/death/drug marketing. During continuation of the drug, information about tumor progression evaluation, survival and drug-related SAEs will be collected as supplemental data.

g. Past medical history: It must include diagnosis history of melanoma, diagnosis date, previous treatment history of melanoma (including surgical treatment, radiotherapy, chemotherapy, hormone therapy, immunotherapy and other therapies), tumor progression within 6 months before signing informed consent form, concomitant disease, history of smoking, history of drinking, allergy history, history of surgery and trauma (surgery history includes operation with significance, such as gastrointestinal endoscopy, biopsy and other diagnostic or therapeutic non-invasive procedures).

h. Body height and weight: Body height will be recorded only at screening visit to calculate BMI. Afterwards, only body weight will be measured before each dose and at EOT visit.

i. ECG examination does not need to be repeated at baseline if its screening examination is within 7 days before baseline, unless the investigator considers it is necessary to receive examination again based on patients’ conditions.

j. Clinical laboratory tests (hematology, blood chemistry, and urinalysis): If clinical laboratory tests in the screening period are performed within 7 days before the first dose, they will not need to repeat on C1D1 and the screening results will be taken as baseline data.

k. Blood coagulation function will be only tested within 7 days before tumor tissue biopsy, and it is not required to be tested if there is no tumor tissue biopsy. In addition, this test is necessary before additional tumor tissue biopsy, which is required by the investigator according to change in patient's condition during the trial.

l. Tests in Note 1 (body weight, physical examination, vital signs, ECOG performance status score, hematology, blood chemistry, urinalysis, photography and measurement of superficial lesions, pre-dose evaluation) will be performed before each dose.

m. Pregnancy test: Female patients of childbearing potential (including early menopause, menopause < 2 years and non-surgical sterilization) must receive blood pregnancy test in the screening period, at EOT visit and at the discretion of the investigator.

n. Tumor tissue biopsy: In the screening period, investigators need to collect tumor tissues and send the tissues to the pathological department for test. Blood coagulation function should be tested before each biopsy. At week 6 of neoadjuvant therapy, additional biopsy will be performed at the discretion of the investigator and with consent of the patient. Blood coagulation function should be tested before each biopsy and biopsy samples will be collected and sent to the pathological department for test. Tissue biopsy may not be performed if there is no site for collecting lesions. Investigator can add tumor tissue biopsy according to change in patient's condition during the trial.

o. For biomarker test, about 20 mL of blood will be collected with 4 whole blood tubes and 1 serum collection tube.

p. Tumor imaging evaluation: Whole-body PET-CT scan and CT/MRI scan of other clinically indicated sites will be performed within 28 days before enrollment. The PET-CT and CT/MRI scans before signing ICF is acceptable for those within 28 days before enrollment, which don't need to be repeated unless the investigator considers tumor burden of the patient has been changed. At week 12 (± 7 days) during neoadjuvant therapy, whole-body PET-CT scan and CT/MRI scan of all other clinically indicated sites will be performed. During adjuvant therapy, head (if necessary), chest, abdomen, pelvis CT/MRI scan and CT/MRI scan of all other clinically indicated sites will be performed every 12 weeks (± 7 days) using the assessment method consistent with that in the screening period/baseline period until PDr or PD, neoplasm recurrence, unacceptable toxicity, loss to follow-up, death, withdrawal of informed consent or EOS, whichever occurs first. If clinically indicated, bone scan should be performed. If the investigator considers it is necessary to have additional tumor imaging evaluation based on clinical evaluation results to clarify PD, recurrence or response, unscheduled imaging evaluation can be arranged. If patients discontinue treatment due to PD or neoplasm recurrence, tumor imaging evaluation will not be required at EOT visit.

q. Tumor related clinical evaluation: The investigator needs to carry out tumor related clinical evaluation of patients (including but not limited to symptoms, signs, size of superficial tumor, and laboratory tests) every 6 weeks during neoadjuvant treatment period. If the investigator considers it is necessary to have additional tumor imaging evaluation based on tumor related clinical evaluation of patients to confirm PD, recurrence or response, unscheduled imaging evaluation can be arranged.

r. Superficial lesions will be photoed and measured with callipers at baseline, before each dose and at EOT visit. That is, superficial lesions larger than 10 mm in diameter that cannot be examined by CT or MRI will be measured with callipers. In case of skin lesions, colorful photos will be taken as records and photos will indicate size and proportion of lesions measured. If there are multiple lesions, the lesions should be numbered. If the lesions can be clinically measured and radiographically examined, CT or MRI must be performed at the time point of tumor imaging evaluation. During clinical measurement and recording, whether there are signs of efficacy with drug therapy should be observed particularly: for example, whether there is tumor shrinkage, flattening, necrosis, erythema, vitiligo, inflammation of one or multiple tumors, etc.

s. Pre-dose evaluation: Patients should receive pre-dose evaluation within 24 hours before each dose to determine injection dose of study drug. OrienX010: Long diameter will be measured with callipers and recorded. Subcutaneous deep lesions can be estimated with B-mode ultrasound and their long diameter should be

recorded, at the discretion of the investigator. According to CT/MRI measurements (lesions that cannot be measured with CT/MRI will be measured with callipers. Subcutaneous deep lesions will be estimated using B-mode ultrasound, at the discretion of the investigator), injection dose of each lesion and total injection dose will be calculated with the longest diameter of lesions and records will be made; toripalimab injection: administration dose will be calculated based on body weight of patients.

t. Throughout the study, in case of cold sores or other suspected lesions caused by herpes virus after injection of OrienX010, it must be wiped with cotton swab for sampling.

V Table of Contents

I Signature Page: Funder 2
II Signature Page: Investigator 3
III Protocol Synopsis 4
IV Schedule of Activities 16
V Table of Contents 23
VI List of Abbreviations 26
1 Ethics 29
1.1 Ethics Committee (EC) 29
1.2 Ethical Guidelines of the Study 29
1.3 Informed Consent of Patients 29
2 Study Background 30
2.1 Overview of Disease 30
2.2 Study Drug[12] 31
2.3 Benefit and Risk Assessment 51
3 Study Objectives 54
3.1 Primary Objectives 54
3.2 Secondary Objectives 54
4 Study Plan 55
4.1 Overall Study Design 55
4.2 Dose Adjustment Regimen 56
4.3 Duration of Study 58
4.4 Discussion of Study Design 58
5 Study Population 60
5.1 Inclusion Criteria 60
5.2 Exclusion Criteria 60
5.3 EOT and patients' withdrawal from the study 62
5.4 Study Termination Principle 63
6 Study Treatment 63
6.1 Characteristics of Study Drug 63
6.2 Administration Method of Study Drug[12] 64
6.3 Precautions of Administration 66
6.3.1 Precautions of OrienX010 Administration 66
6.3.2 Precautions of Administration of Toripalimab Injection 66
6.3.3 Continued administration 70
6.4 Criteria for interruption/permanent discontinuation of OrienX010 and toripalimab injection 71
6.4.1 Criteria for interruption/permanent discontinuation of OrienX010 therapy 71
6.4.2 Criteria for interruption/permanent discontinuation of toripalimab injection 71
6.5 Package and Label of the Study Drug 71
6.6 Storage and Management of Study Drugs 71
6.7 Handling of Clinical Events 72

6.8 Concomitant Medications and Supportive Therapies 72
6.9 Study Compliance 73
6.10 Randomization 74
6.11 Blinding 74
7 Study Procedures 75
7.1 Schedule of Study Visits 75
7.1.1 Screening Visits (D-28 to D-1) 75
7.1.2 Treatment Period 76
7.1.3 EOT visit (day 28 ± 7 days after the last dose) 79
7.1.4 Follow-up of neoplasm recurrence 80
7.1.5 Survival follow-up (every 30 days ± 7 days after EOT) 80
7.1.6 End of Study 80
7.2 Study Assessment 81
7.2.1 Physical examination 81
7.2.2 Height and Weight 81
7.2.3 Vital signs 81
7.2.4 12-lead ECG 81
7.2.5 ECOG performance status score 82
7.2.6 Laboratory Tests 82
7.2.7 Cotton Swab for Wiping and Sampling 82
7.2.8 Pre-dose Evaluation 83
7.2.9 Tumor Radiographic Images 83
7.2.10 Photography and caliper measurement of superficial lesions 83
7.2.11 Tumor tissue and blood sample collection for biomarker test 84
8 Efficacy Evaluation 85
8.1 Efficacy Measures 85
8.2 Efficacy Evaluation Methods 85
9 Adverse Events 87
9.1 Definition of Adverse Events 87
9.2 Report of Serious Adverse Event 88
9.3 Handling and Follow-up of AEs 89
9.4 Adverse Events of Special Interest 89
9.4.1 AESIs of OrienX010 89
9.4.2 AESIs of toripalimab injection 89
9.4.2.1 Allergic Reactions 89
9.4.2.2 Infusion Reactions 90
9.4.2.3 Immune-related Adverse Events (irAEs) 92
9.5 Pregnancy 96
10 Statistical Analysis 99
10.1 Determination of Sample Size 99
10.2 Populations for Statistical Analysis 99

10.3 General Principles for Statistical Analysis 99
10.4 Statistical Analysis Method 99
10.4.1 Handling of Drop-out or Missing Data 99
10.4.2 Multi-center Study 99
10.4.3 Statistical Analysis 99
10.4.4 Demographics and Other Baseline Characteristics 100
10.4.5 Analysis of Compliance and Drug Exposure 100
10.4.6 Efficacy Analysis 100
10.4.7 Safety Analyses 100
10.4.8 Interim Analysis 101
10.4.9 Subgroup Analysis 101
11 Data Management 102
11.1 Case Report Form 102
11.2 Data Entry and Modification 102
11.3 Locking of Database 103
12 Quality Assurance 104
12.1 Monitoring 104
12.2 Audit and Inspection 104
13 Study Management 105
13.1 Imaging Assessment 105
13.2 Management Structure 105
13.3 Preservation of Original Data 105
13.4 Recording of Study Data 105
13.5 Confidentiality 105
13.6 Review of the Ethics Committee (EC) 106
13.7 Protocol Amendment 106
13.8 Publication of Study Results 106
13.9 Protocol Compliance and Protocol Violation 106
13.10 Clinical Study Report 107
13.11 The final clinical study report will be written according to the NMPA guidelines for structure and contents of clinical study report. The final clinical study report must be prepared no matter whether the study is completed or prematurely terminated. Insurance, Compensation and Indemnification 107
13.12 Termination of the Study 107
13.13 Management of Study Site Documents 107
14 References 108
Appendixes I ECOG Performance Status Scoring Criteria 109
Appendixes II Response Evaluation Criteria in Solid Tumors (RECIST) 1.1 110

VI List of Abbreviations

Abbreviations	English Name
5-FU	5-Fluorouracil
ADL	Instrumental Activities of Daily Living
AE	Adverse Event
AESI	Adverse Event of Special Interest
ALB	Albumin
ALP	Alkaline Phosphatase
ALT	Alanine Aminotransferase
APTT	Activated Partial Thromboplastin Time
AST	Aspartate Aminotransferase
BIL	Bilirubin Urine
BSA	Body Surface Area
NMPA	National Medical Products Administration
CK	Creatine kinase
CL1	Wild-Type Herpes Simplex Virus 1
CR	Complete Response
Cr	Creatinine
CRA	Clinical Research Associate
CRO	Contract Research Organization
CT	Computerised Tomography
CTCAE	Common Terminology Criteria for Adverse Events
CTX	Cyclophosphamide
DBIL	Direct Bilirubin
DCR	Disease Control Rate
DOR	Duration of Response
DRR	Durable Response Rate
EC	Ethics Committee
ECG	Electrocardiogram
ECOG	Eastern Cooperative Oncology Group
eCRF	Electronic Case Report Form
EOS	End of Study
EOT	End of Treatment
FAS	Full Analysis Set
FDA	Food and Drug Administration
FPG	Fasting Plasma Glucose
GCP	Good Clinical Practice
GLP	Good Laboratory Practice
GLU	Glucose Urine
GM-CSF	Recombinant Human Granulocyte-Macrophage Colony-Stimulating Factor
Hb	Hemoglobin
HBsAg	Hepatitis B Surface Antigen
HBV	Hepatitis B Virus
HCT	Hematocrit

Abbreviations	English Name
HCV	Hepatitis C Virus
HDL-C	High Density Lipoprotein Cholesterol
HIV	Human Immunodeficiency Virus
HSV-1	Herpes Simplex Virus 1
ICF	Informed Consent Form
ICH	International Conference on Harmonization
iCPD	Progressive Disease Confirmed Based on iRECIST
iUPD	Progressive Disease Unconfirmed Based on iRECIST
INR	International Normalised Ratio
irPR	Immune-Related Partial Response
irSD	Immune-Related Stable Disease
ITT	Intention To Treat (Analysis Set)
KET	Urine Ketone Body
LDH	Lactate Dehydrogenase
LDL-C	Low Density Lipoprotein Cholesterol
MCV	Mean Corpuscular Volume
MCH	Mean Corpuscular Hemoglobin
MCHC	Mean Corpuscular Hemoglobin Concentration
MedDRA	Medical Dictionary for Regulatory Activities
MRI	Magnetic Resonance Imaging
OrienX010	Recombinant Human GM-CSF Herpes Simplex Virus Injection
ORR	Objective Response Rate
OS	Overall Survival
PD	Progressive Disease
PDr	Clinical Related Progressive Disease
PFS	Progression-free Survival
pH	Potential of Hydrogen
PLT	Platelet
PPS	Per-protocol Set
PR	Partial Response
pPR	Pathologic Partial Response
Major PR/Near pCR	Near Pathological Complete Response
pCR	Pathologic Complete Response
PRO	Urine Protein
PT	Prothrombin Time/Preferred Term
PTT	Partial Thromboplastin Time
QoLS	Quality of Life Set
RBC	Red Blood Cell Count
RECIST	Response Evaluation Criteria in Solid Tumors
SAE	Serious Adverse Event
SAS	Safety Analysis Set
SD	Stable Disease

Abbreviations	English Name
SG	Specific Gravity
SOP	Standard Operating Procedure
SS	Safety Set
TBIL	Total Bilirubin
TC	Total Cholesterol
TEAE	Treatment-emergent Adverse Event
TG	Triglyceride
TP	Total Protein
ULN	Upper Limit of Normal
UREA	Urea
URIC	Uric Acid
URO	Urobilinogen
WBC	White Blood Cell Count
WHO-DD	World Health Organization Drug Dictionary

1 Ethics

1.1 Ethics Committee (EC)

The study will be conducted according to guidelines of the Ethics Committee (EC) and Good Clinical Practice (GCP)[1] and relevant regulations and data protection requirements.

Before starting the study, written and dated approval of EC for protocol/protocol amendment, informed consent form, other updated consent forms, patient recruitment procedure (such as advertisement) and other literal documents provided to patients should be obtained. EC's approval letter should indicate version number of protocol and name of each reviewed document.

1.2 Ethical Guidelines of the Study

The study will be conducted in compliance with GCP guidelines, the Declaration of Helsinki (2013) and all relevant regulatory requirements.

1.3 Informed Consent of Patients

The investigator will explain benefits and risks from taking part in the study to each patient, patient's legal representative or witness and obtain their written informed consent. Written informed consent of patients should be obtained before taking part in the study or receiving any study-related procedures (including use of study drugs).

The investigator will preserve the original informed consent form that has been signed and dated by patient or legal representative and relevant personnel taking part in informed consent discussion as study document. The investigator will provide a copy of signed informed consent form to all patients.

During the study progress, the informed consent form should be revised if new important information related to the safety of patients is available. In such case, EC's approval should be obtained and patients who have previously signed the informed consent form should be informed of the updated information and receive informed consent again. The process will be also recorded.

After obtaining patients' consent, the investigator can tell patients' treating doctor that the patients will take part in the clinical study.

2 Study Background

2.1 Overview of Disease

Melanoma, also known as malignant melanoma, is a malignant tumor from melanocytes and is commonly observed in the skin, mucosa and ocular choroid, etc. It is one of the common malignant tumors in clinical practice and one of the malignant tumors that has the fastest growing incidence, with an annual growth rate of 3-5%. In 2012, there were 232,000 new cases and 55,000 deaths of melanoma worldwide[2]. In China, the prevalence of melanoma is low. However, with a trend of rapid increase in recent years, there are about 20,000 new cases every year. According to the data of previous Chinese cancer registry annual reports, mortality of melanoma in China tends to increase in a fast manner year by year[3], and melanoma has become one of the diseases that seriously threatens the health of Chinese people.

In China, patients who have stage III or IV melanoma at the first presentation account for over 35% and the prognosis is poor. According to statistics, the median survival of stage M1a melanoma, stage M1b melanoma, bone metastases, hepatic and brain metastases is 15 months, 8 months, 6 months and 4 months, respectively. The overall median survival of metastatic melanoma is 7.5 months only, with 2-year survival of 15%[4]. Resection of primary melanoma or dissection of regional lymph nodes only is not enough to block tumor progression. Adjuvant therapy can be carried out to improve patients' survival. Anti-PD-1 monoclonal antibodies which have been studied as adjuvant therapy after operation for melanoma demonstrate it can prolong RFS of patients and has been approved by FDA in 2017 as adjuvant therapy of cutaneous melanoma. In the same year, a study of T-VEC combined with anti-PD-1 antibody for the treatment of melanoma showed that tumor response rate with combination therapy reached 62% and the CR was 33%[5]. In June 2018, a study published on JAO reported that T-VEC combined with monoclonal antibody achieved significant efficacy for treatment of melanoma. Combination of the two drugs as preoperative neoadjuvant therapy may provide more benefits to patients.

Toripalimab injection (JS001) is a self-developed novel recombinant humanized (97%) anti-PD-1 monoclonal antibody (Chinese patent authorization number CN104250302B), and can specifically bind to PD-1 and effectively block the interaction between PD-1 and its ligand PD-L1 (i.e., B7-H1) and PD-L2 (i.e., B7-DC), so as to activate cytotoxic T lymphocytes and inhibit the growth of tumors.

It is a Class 1 new therapeutic biological product. On December 27, 2015, approval (No.: 2015L05752) of drug clinical trial was obtained from National Medical Products Administration (NMPA). On December 17, 2018, this product was approved by NMPA for the treatment of unresectable or metastatic melanoma that failed previous systemic therapy. The drug approval document number is NMPA S20180015.

T-Vec is manufactured by Amgen in the USA. On October 27, 2015, US Food and Drug Administration (FDA) approved OncoVex $^{GM\text{-}CSF}$ (T-Vec) for topical treatment of unresectable skin, subcutaneous and lymph node lesions of patients with relapsed melanoma after the first surgery. This is the first FDA-approved oncolytic virus therapeutic drug and is a genetically modified herpes simplex virus 1 (HSV-1). It can be copied in tumor cells and express

granulocyte-macrophage colony-stimulating factor (GM-CSF) of immune activator protein. Direct injection into melanoma can cause tumor cell lysis, leading to tumor cell rupture, releasing tumor-derived antigen and GM-CSF, and accelerating anti-tumor immune response. As seen from study results of marketed T-Vec, it has certain efficacy, good safety and tolerability in topical treatment of unresectable skin, subcutaneous and lymph node lesions of patients with relapsed melanoma after the first surgery. However, no related product has been marketed in China.

2.2 Study Drug[12]

GM-CSF Herpes Simplex Virus Injection (hereinafter referred to as OrienX010) independently developed by OrienGene Biotechnology Ltd. is a gene therapy drug with the genetically engineered herpes simplex virus 1 (HSV-1) as the carrier which is inserted with GM-CSF. Its carrier is wild-type herpes simplex virus 1 (CL1 strain) isolated from the oral cavity of Chinese patients, and through the gene recombination technology, ICP34.5 and ICP47 genes are deleted, inactivated ICP6 gene is inserted, and human GM-CSF gene is inserted at the site of the original ICP34.5 under the guidance of the IE promoter of hCMV. It's anti-tumor mechanism is similar to that of T-Vec. After this drug is locally injected at the tumor site, the anti-tumor effect can be achieved through two mechanisms: On the one hand, due to the oncolytic characteristic of herpes simplex virus, the modified HSV-1 virus carrier kills tumor cells at the injection site in a specific "oncolytic" manner; on the other hand, the virus carrier expresses and produces high-level GM-CSF at the tumor site to enhance the antitumor immune function, plays a "bystander killing effect", and produces an inhibitory effect on the distant metastases.

After the modified HSV-1 virus carrier enters tumor lesions by local injection, it kills tumor cells at the injection site in a specific “oncolytic” manner through the following mechanism:

- Due to the shutdown of protein synthesis, ICP34.5-deleted HSV-1 cannot replicate in normal cells or tissues, but it still can replicate in malignant tumor cells. So it plays a selectively oncolytic role.
- The deletion of ICP47 makes the vector-infected tumor cells easier to be recognized by the immune system, thereby enhancing the presentation of tumor antigens (released after tumor cells are destroyed by the oncolytic vector) to the immune system and amplifying the anti-tumor immune response. On the other hand, ICP47 deletion enhances the replication ability of tumor cells in the viral vector, improving the oncolytic ability of the vector.
- Insertion of inactivated ICP6 gene can further enhance the high selectivity and specificity of OrienX010 to tumor cells, improving the safety of the product. In addition, the HSV-1 carrier inserted with inactivated ICP6 gene has neurotoxicity decreased and high sensitivity to anti-viral drugs acyclovir and ganciclovir.

The virus carrier expresses and produces high-level GM-CSF at the tumor site to enhance the antitumor immune function, and plays a “bystander killing effect” through the following mechanism:

- GM-CSF can enhance the activity of granulocytes and macrophages, increase the expression of MHC-I and II molecules and B7 molecule, and promote the maturation of

antigen presenting cells (especially dendritic cells), thereby greatly enhancing the immune efficiency of gene vaccines. It can also mobilize bone marrow to promote proliferation and differentiation of bone marrow hematopoietic stem cells, inhibit apoptosis of white blood cells, and thereby increase the count of white blood cells in peripheral blood.

- Following the local intratumral injection of modified GM-CSF gene carried by a viral vector, only its bioactive GM-CSF proteins were stably expressed in the tumor and the infiltration of $CD4^+$ and $CD8^+$ lymphocytes, macrophages, and eosinophils at the intratumral injection site was observed; in addition, the amount of GM-CSF expressed by the therapeutic vector in the tumor was not sufficient to affect the changes of GM-CSF level in peripheral blood, and no proportional changes in white blood cell count or polymorphonuclear leukocytes, monocytes, and eosinophils in peripheral blood were observed.
- GM-CSF carried by the viral vector produces GM-CSF proteins in a low amount and constantly and stably in the form of autocrine or paracrine at the injection site, directly acting on the immune effector cells at the injection site; meanwhile, the replication of the viral vector causes necrosis of tumor cells to release tumor antigens, and the immune response induced by such antigens is magnified by GM-CSF to produce a systemic antitumor effect, but there is no effect on the normal hematopoietic system.

Pre-clinical Studies

CMC studies

OrienX010 is a Recombinant Human GM-CSF Herpes Simplex Virus Injection with the PBS buffer containing 2% polysaccharides as its vehicle. It is in a white frozen state at -18°C. After thawing and reconstitution, it is a colorless or off-white suspension liquid without foreign matters or visible particles. The pH in liquid state is in the range of 6.5-7.5.

General Pharmacology Studies

General pharmacology study of locomotor activities of BALB/c mice

This trial set 3 groups, i.e., PBS vehicle control group, OrienX010 low-dose group (5×10^8 pfu/kg), and OrienX010 high-dose group (2.5×10^9 pfu/kg), with 10 mice (5/sex) in each. Throughout this trial, the mice were dosed once via intramuscular injection. The results showed: Through the comparison of the 0.5h, 1h, 2h, 3h, 4h post-dose locomotor activities of the mice to their pre-dose activities, OrienX010 had no effect on the locomotor activities of BALB/c mice.

General pharmacology study on intramuscular injection in Rhesus monkeys

This trial set 3 groups, i.e., PBS vehicle control group, OrienX010 low-dose group (2.5×10^7 pfu/kg), and OrienX010 high-dose group (1.25×10^8 pfu/kg), with 6 rhesus monkeys (3/sex) in each. Throughout this trial, the rhesus monkeys were dosed once via intramuscular injection at a dose volume of 250 µl. The results showed that intramuscular injection of PBS vehicle, low- and high-dose OrienX010 had no effect on the body temperature, blood pressure, heart rate, and ECG of rhesus monkeys.

Results of pharmacodynamic studies

Summary of OrienX010 antitumor primary pharmacodynamics study (in vitro)

This trial tested the effects of OrienX010 on the growth of in vitro xenograft cells. The active drug was 5-fluorouracil (5-FU). The test cells included: human lung cancer cell A549, human breast cancer cell MCF-7, human gastric cancer cell BGC-823, human gastric cancer cell SGC7901, human liver cancer cell HepG2, human liver cancer cell SMMC-7721, human colon cancer cell HT-29, human colon cancer cell CaCo2, human colon cancer cell HCT, human esophageal cancer cell Eca109, human esophageal cancer cell SHEEC, human cervical cancer cell Hela, murine melanoma cell B16-BL6, human tongue cancer cell KBV-200, human oral cancer cell KB, 15 types of tumor cells in total.

OrienX010 concentrations were 40, 20, 10, 5, 2.5, 1.25, 0.625, 0.3125 pfu/cell. The results showed: OrienX010 exhibited a good dose-dependent relationship to the growth suppression of all test cells, the duplicate tests results were consistent, and the setting concentrations of the test article basically covered the drug effect window. The mean EC_{50} was ranged from 0.836 pfu/cell to 3.835 pfu/cell.

Compared to the growth suppression effect of the routine chemotherapeutic drug 5-FU (0.1 μg/ml) on the test cells, the potency of OrienX010 was close. With close observation time and within the set concentration range of the test article, the maximum inhibitory effect on the growth of test cells was ranged from 86.5% to 100.0%.

Summary of OrienX010 antitumor primary pharmacodynamics study (in vivo)

This experiment tested the effects of OrienX010 on the growth of human colonic adenocarcinoma HT-29 xenografts in nude mice, human breast cancer MCF-7 xenografts in nude mice, human tongue cancer KBV-200 xenografts in nude mice, human lung adenocarcinoma A549 xenografts in nude mice, and B16-BL6 murine melanoma, and using Cyclophosphamide for Injection (CTX) as the positive control drug.

Three OrienX010 [low (5×10^5 pfu/tumor), medium (5×10^6 pfu/tumor), and high (1×10^7 pfu/tumor)] dose groups, a blank control group and an active drug group were set. The results showed that, OrienX010 had an inhibitory effect on the xenografts of each kind of test tumor and after two duplicate tests, the inhibition rate of OrienX010 dose groups on human colonic adenocarcinoma HT-29 xenografts in nude mice was ranged from 28.0% to 77.0%; there was a significant dose-response relationship between dose groups, and the potency of OrienX010 was comparable to that of the active drug CTX (100 mg/kg×2); and the dose-response relationship between the medium-dose group and low-dose group was more significant.

Summary of OrienX010 antitumor primary pharmacodynamics study (validation of GM-CSF effect)

This trial tested the effect of OrienX010 and GM-CSF (expressed by OrienX010) on the growth of murine ascitogenous hepatoma H22 xenografts in Kunming mice. The results showed: OrienX010 had an inhibitory effect on the growth of murine ascitogenous hepatoma H22 xenografts in Kunming mice. Both of the human OrienX010 and murine OrienX010 dose groups had a significant inhibitory effect on the growth of murine ascitogenous hepatoma H22

xenografts in Kunming mice, where the mean inhibition rate of human OrienX010 was 46.06% to the dosed tumor masses at the head and 35.02% to the non-dosed tumor masses at the tail; while the corresponding mean inhibition rates of murine OrienX010 were 78.87% and 61.06%, respectively. Compared to human OrienX010, murine OrienX010 had a significantly higher inhibition rate to murine H22 xenografts and a more significant "bystander killing effect" (61.06% vs 35.02%), suggesting that GM-CSF expressed by the test article had a clear effect.

Toxicological Studies

1) Acute toxicity tests

Acute toxicity study of OrienX010 intravenous injection in BALB/c mice

BALB/c mice were intravenously injected with OrienX010 via a caudal vein by the maximum tolerated dose method. Three groups, i.e., PBS vehicle control, human OrienX010 group (4×10^7 pfu/animal), and murine OrienX010M group [including high-dose group (3×10^7 pfu/animal), medium-dose group (2×10^7 pfu/animal), and a low-dose group (1×10^7 pfu/animal)] were designed for the entire acute toxicity study. Conclusion based on study results: for caudal vein injection to BALB/c mice, the maximum tolerated dose of human OrienX010 and murine OrienX010M was 4×10^7 pfu and 2×10^7 pfu, respectively.

Acute toxicity study of OrienX010 intramuscular injection in BALB/c mice

BALB/c mice with 6 weeks of age were intramuscularly injected with OrienX010 by the maximum tolerated dose method for acute toxicity study. The study consisted of 3 groups: PBS vehicle control group, human OrienX010 group, and murine OrienX010M group, the dosage used was 6×10^7 pfu/animal for all groups and the dose volume was up to 0.6 ml/animal. Conclusion based on study results: for intramuscular injection to BALB/c mice with 6 weeks of age, the maximum tolerated dose was 6×10^7 pfu for both of human OrienX010 and murine OrienX010M.

Corpus striatum-related neurotoxicity evaluation study of OrienX010

This trial explored the toxic side effects of OrienX010 intracerebral injection on the corpus striatum-related nervous system of rats. Rats were randomly divided into 2 groups. In Group 1 (control group, n = 12), 3 μl of 0.9% normal saline was injected into the rat's right corpus striatum via stereotactic injection; in Group 2 (treatment group, n = 16), 3 μl of OrienX010 was injected into rat's corpus striatum via stereotactic injection. The results showed: In the control group, there were no abnormal changes in the neurological symptom scores, forelimb grip test, and balance exercise test at 6h, 24h, and 10d, and pathological observation revealed injection site vasodilation and peripheral edema at 24h, which returned to normal at 10d; in the treatment group, pathological observation at 10d after administration revealed significant neurological symptoms and pathological changes of one female rat, and mild pathological changes were also observed in the cerebral pathological sections of other female rats, but there was no significant change in the behavioral symptoms, and no abnormal changes were observed in the cerebral pathological sections of male rats.

2) Long-term toxicity studies

Repeat-dose toxicity study of OrienX010 intramuscular injection in BALB/c mice

According to the requirements of China's Good Laboratory Practice (GLP), a toxicity study of OrienX010 intramuscular injection was conducted in BALB/c mice to observe the toxic effects of OrienX010 on the body and severity of such effects, main target organ of toxicity, and reversibility of damage. This trial chose SPF BALB/c mice with 6 weeks of age (age at the time of arrival). A total of 5 dose groups were set for human and murine OrienX010, specifically: PBS vehicle control group, human OrienX010 low-dose (5×10^8 pfu/kg) and high-dose (2.5×10^9 pfu/kg) groups, as well as murine OrienX010 low-dose (5×10^8 pfu/kg) and high-dose (2.5×10^9 pfu/kg) groups.

Throughout this trial, the mice were dosed 7 times via intramuscular injection. The test results showed: OrienX010 did not cause any biologically significant changes in the animals' general clinical symptoms, injection site, hematology, serum chemistry, or organ weights, and pathological examination did not reveal any OrienX010-related histopathological changes. After intramuscular injection to the mice, anti-HSV-1 antibodies were produced in the animals, but no expression of human and murine GM-CSF caused by human and murine OrienX010 was detected, and there was no significant effect on the percentages of CD^{4+} and CD^{8+} cells or CD^{4+}/CD^{8+} ratio.

Long-term toxicity study of OrienX010 repeated intramuscular injection in Rhesus monkeys

The test animals were conventional Rhesus monkeys with 3 years of age. This test set three groups: PBS control group, OrienX010 low-dose (2.5×10^7 pfu/kg) group, and OrienX010 high-dose (1.25×10^8 pfu/kg) group. The animals were dosed for a total of 7 times via intramuscular injection. The test results showed that long-term intramuscular injection of OrienX010 to Rhesus monkeys at a dose level 20 times higher than the intended clinical dose was safe and did not cause any significant toxic side effects.

Long-term toxicity study of OrienX010 repeated intramuscular injection in SD rats

In this study, 150 SD rats (75/sex) were randomly divided into 5 groups (30 animals/group, 15/sex), specifically the vehicle control group, positive control low-dose group, positive control high-dose group, OrienX010 low-dose group, and OrienX010 high-dose group. Animals in the dose groups were intramuscularly injected with OrienX010 once weekly at the dose levels of 0.8×10^6 (murine), 8.0×10^7 (murine), 8.0×10^6 (OrienX010), and 8.0×10^7 (OrienX010) pfu/kg, respectively. The high dose of OrienX010 was the maximum dosage used under the conditions of this study. The animals were dosed for a total of 27 times via intramuscular injection.

The test results showed that long-term intramuscular injection of OrienX010 to SD rats at a dose level 13 times of the intended clinical dose (i.e., $\leq 8.0\times10^7$ pfu/kg) was safe and did not cause any significant toxic side effects.

In vivo Biological Distribution Studies

1) **Biological distribution of OrienX010 repeated intramuscular injection in Rhesus monkeys**

In the long-term toxicity study of OrienX010 repeated intramuscular injection in Rhesus

monkeys, the tissue distribution of OrienX010 was determined concurrently. The results indicate that, only trace distribution of OrienX010 was detected in a few organs or blood and urine of a few animals at 24h after 7 intramuscular injection doses to Rhesus monkeys, and no distribution of OrienX010 was detected at 2 months of recovery.

2) Biological distribution of OrienX010 intramuscular injection in normal mice

The study chose clean BALB/c mice with 6 weeks of age and set three groups: human OrienX010 group (5×10^8 pfu/kg, i.e., 1×10^7 pfu/animal), murine OrienX010 group (5×10^8 pfu/kg, i.e., 1×10^7 pfu/animal), and normal tissue extraction group. The animals were dosed for a total of 3 times via intramuscular injection.

The study results indicate that, the tissue distribution profiles of human and murine OrienX010 were essentially consistent in animals. At 24 hours after the last dose, distribution of OrienX010 was detected in all the tissues and organs sampled, predominantly in the nerve tissues and injection site muscle; at 2 months after the last dose, distribution of OrienX010 was detected in all the tissues and organs sampled, the tissue distribution was relatively higher in the dorsal root ganglion, while the distribution in other organs was significantly decreased compared to that at 24 hours after the last dose, showing a trend that the tissue distribution decreases and disappears gradually over time.

Other safety studies

Active anaphylaxis test of OrienX010 intramuscular injection in guinea pigs

This trial investigated whether any test article-induced anaphylaxis occurs after intramuscular injection of OrienX010 to guinea pigs to provide reference for its clinical studies. Method: Hartley guinea pigs with 4 weeks of age were chosen and divided into the negative control group (PBS vehicle), positive control group (bovine serum albumin), OrienX010 low-dose group (2.5×10^7 pfu/kg) and OrienX010 high-dose group (1.25×10^8 pfu/kg). A back-up animal group was additionally set. The results showed: Systemic anaphylaxis was negative in guinea pigs following intramuscular injection of OrienX010.

Clinical studies

At present, a phase 1a clinical study and a phase 1b clinical study of OrienX010 intratumoral injection have been completed. A phase 1b clinical study and a phase 1c clinical study are ongoing. This section summarizes clinical study progress of OrienX010 and lists phase 3 clinical study results of T-Vec for reference.

Phase 1a clinical study of OrienX010 (OrienX010-II-07)

"An Open-label, Dose-escalation, Phase 1 Clinical Trial of Recombinant Human GM-CSF Herpes Simplex Virus Injection (OrienX010) intratumral Injection in the Treatment of Malignant Tumors in Adults" was conducted and completed between April 2012 and December 2014 in Fudan University Shanghai Cancer Hospital and Beijing Shijitan Hospital, Capital Medical University.

This is a two-center, open-label, dose-escalation, phase 1 clinical trial. The target population of this study was patients with advanced malignant tumors that lack effective usual care or have

failed or relapsed after the usual care. All patients received OrienX010 monotherapy. This study consisted of two parts (single-dose trial and multi-dose trial), single-dose and multi-dose dose-escalation were carried out successively. There was a 4-week observation period following single intratumoral injection of OrienX010, the 4 dose groups were 10^6 pfu, 10^7 pfu, 10^8 pfu, and 4×10^8 pfu, and in the multi-dose trial, OrienX010 was injected once every two weeks for a total of 3 times at the dose levels of 10^8 pfu and 4×10^8 pfu, respectively. Meanwhile, the safety evaluation, biological distribution, biological effect study and preliminary efficacy observation of single-dose and multi-dose OrienX010 were conducted concurrently.

This trial enrolled 24 patients in total, of whom 18 completed the trial and 6 withdrew from the trial midway due to adverse reaction or progressive disease (PD) or other reasons. The tumor types of the patients included: malignant melanoma (8 cases), breast cancer (5 cases), cutaneous anaplastic large cell lymphoma (2 cases), rectal cancer (2 cases), carcinoma of submaxilary gland (2 cases), stromal tumor of the small intestine (1 case), gingival carcinoma (1 case), metastatic squamous cell carcinoma in lymph nodes (1 case), lung cancer (1 case), and malignant thymoma (1 case). All patients had baseline Eastern Cooperative Oncology Group (ECOG) score data, with 13 cases having a score of 0 and the rest 11 having a score of 1. All subjects were positive for HSV-1-IgG in the anti-HSV-1 antibody serological test at baseline.

Safety

In this trial, no dose-limiting toxicity (DLT) case was observed in the four dose groups of single-dose administration or two dose groups of multi-dose administration during dose-escalation. No maximum tolerated dose (MTD) was observed in this study.

The overall incidence of adverse events (AEs) and the incidences of AEs per category were basically comparable between dose groups. They were also basically comparable between single-dose group and multi-dose group, except the incidences of fever and injection site reactions were higher in the multi-dose group (100.0% and 42.9%, respectively) than in the single-dose group (62.5% and 25.0%, respectively).

There were 21 patients who reported at least 1 study drug-related AE. During the dosing period, a total of 66 study drug-related AEs was reported, all of which were Grade 1 to 2 in severity, there were no Grade 3 or above study drug-related AEs, and most AEs resolved spontaneously in 1 to 2 weeks without any treatment measure. The common study drug-related AEs were fever (defined as body temperature ≥ 37.5℃, occurring in 75% of patients), injection site reactions (manifested as pruritus, pain, tension sensation, burning sensation, redness and swelling, and exudation at the injection site, occurring in 29.2% of patients), asthenia (25%), headache (12.5%), and peripheral coldness (8.3%).

During the trial period, a total of 5 serious adverse events (SAEs) occurred, 4 of which resulted in hospitalization (Grade 3 in severity) and were assessed as unlikely related to the study drug by the investigator; the rest 1 was death, whose relationship with the study drug was deemed as not assessable by the investigator.

The main fluctuation in patient's vital signs was the increase in body temperature at 4 to 48 hours post-dose; blood pressure, respiratory rate, heart rate, and body weight basically

remained stable; both pre-dose and post-dose ECOG scores were ≤ 1.

In the abnormal and clinically relevant laboratory test results, blood bilirubin increased (1 case) and urobilinogenuria (1 case) were assessed as possibly related to the drug by the investigator, and other abnormal laboratory values that were definitely unrelated or unlikely related to the study drug mainly included white blood cell, hemoglobin, AST, bilirubin, protein, blood potassium, blood glucose, and urine glucose, each occurring in 1 to 3 patients.

Abnormal and clinically relevant ECG findings included: occasional atrial premature beats, occasional ventricular premature beats, sinus bradycardia, T wave changes with Q-T interval prolongation, each occurring in 1 patient; 1 patient showed T wave changes in both of the baseline and subsequent ECG examinations, while no abnormality was revealed by the subsequent ECG examination of the rest patients.

Efficacy

All the 18 patients of the per protocol analysis set received tumor assessments at baseline and 4 weeks after the single-dose/three-dose administration, among which 1 had PR and was confirmed as SD 4 weeks later, 10 had SD, and 7 had PD. 11 patients (61.1%) achieved clinical benefits (i.e., SD). In the patients who had PD (n = 7, 38.9%), 6 (33.3%) experienced PD at the target lesion sites, and 3 (16.7%) at the non-target lesion sites. In the above 11 patients who achieved clinical benefits, 5 continued the treatment with the study drug and received tumor follow-up assessment, with the follow-up period lasting 85 to 113 days. By the time of the last follow-up, 3 subjects still maintained SD, while the rest 2 had PD. Eight patients received single-dose injection of the study drug at 14 superficial target lesion sites, after which there was no significant change (-18.3% to 18.7%) in the long diameter of 6 injected lesions, while there was a > 20% increase in the long diameter of the rest 8 lesions. Three patients received multiple-dose injection of the study drug at 3 superficial lesions, after which there was no significant change (-4.4% to 4.9%) in the long diameter of the injected lesions. Two patients received single-dose injection of the study drug at 4 deep target lesions, after which there was no significant change (-3.6% to 3.3%) in the long diameter of the injected lesions. Seven patients received multiple-dose injection of the study drug at 7 deep lesions, after which there was no significant change (-24.2% to 9.1%) in the long diameter of 4 injected lesions, while there was a > 20% increase (30.3% to 53.3%) in the long diameter of the rest 3 lesions.

Immunogenicity

In this trial, only patients with positive anti-HSV-1 antibody were screened, therefore, the differences in safety and biological effects between negative HSV-1 antibody group and positive HSV-1 antibody group were not analyzed. There were no statistically significant differences in patients' blood OrienX010 copy number, urine OrienX010 copy number, blood GM-CSF protein concentration, GM-CSF antibody concentration, blood lymphocyte differential test, and antinuclear antibody (ANA) titer test, suggesting that OrienX010 will not cause any significant changes in the results of the above hematological indicators, and the inter-patient differences were caused by patients' individual differences. The blood anti-HSV-1 neutralizing antibody test results showed that OrienX010 injection caused an increase in the anti-HSV-1 neutralizing antibody level in some patients, which may affect the efficacy of

subsequent OrienX010 doses. For injection site needle aspiration and tissue GM-CSF RNA quantitation, the GM-CSF RNA level at the OrienX010 injection site showed no dose-dependent relationship between dose groups under single-dose conditions, but the GM-CSF RNA level at the OrienX010 injection site of patients receiving multiple doses was higher than that of patients receiving a single dose. Active OrienX010 was detected at the injection site of a few patients after OrienX010 injection, but all these patients basically turned negative 24 hours later.

Conclusions

In this study, no MTD and DLT of OrienX010 intratumoral single-dose and multi-dose administrations were observed, and OrienX010 intratumoral injection was well tolerated. The main adverse reactions were injection site reactions and fever. It is suggested that subsequent clinical trials should adopt a minimum dose of 4×10^8 pfu and the dosing interval should not be longer than or exceed 2 weeks. 11 of the 24 patients (45.8%) achieved SD in post-treatment evaluation, and the duration of post-treatment SD was up to 16 weeks in 2 patients. It is suggested that subsequent clinical studies should increase the duration of subject observation and follow-up. In view of the good performance of the foreign predicate product OncoVex$^{GM\text{-}CSF}$ in patients with melanoma, it is suggested to further explore the safety and efficacy of the study drug in malignant melanoma. In addition, compulsory stay in isolation ward is not needed for the future clinical administration.

Phase 1b clinical study of OrienX010 (OrienX010-II-08)

In view of the good performance of the foreign predicate product T-VEC in patients with melanoma and to explore the safety and efficacy of the study drug in malignant melanoma, "An Open-label Phase 1b Clinical Trial of Recombinant Human GM-CSF Herpes Simplex Virus Injection (OrienX010) Intratumral Injection in the Treatment of Stage IIIb, IIIc, and IV M1a Malignant Melanoma that Has failed the Standard Treatment Regimen" was conducted in Beijing Cancer Hospital between April 2014 and August 2015.

This is a single-center, open-label phase 1b clinical trial without any control group.

Subjects received study drug (OrienX010, titer 8×10^7 pfu/ml) via intratumoral injection, with the injection volume determined according to the size of the tumor. If the tumor lesions increase or shrink during the treatment, the dose can be adjusted according to the actual measurement of the lesions prior to each injection. The total volume of each injection did not exceed 5 mL (i.e., 4×10^8 pfu). The frequency of injection was every 2 weeks, 6 weeks per cycle, 3 doses per cycle, 2 cycles in total.

Upon the completion of efficacy evaluation at the end of Cycle 2, if in the judgment of investigator, the patient can benefit from the continued administration, the study drug can be continued until PD, intolerable toxicity, or patient's withdrawal of informed consent.

This clinical trial enrolled 12 subjects in total as of data cutoff (April 11, 2015), of whom 10 completed all observations of the clinical trial, and 2 subjects withdrew midway due to PD. Five subjects had stage IIIc melanoma, 4 had stage IV M1a melanoma, 2 had stage IV M1b melanoma, and 1 had stage IV M1c melanoma. All patients had baseline ECOG score data, with 1 patient having a score of 0 and the rest 11 having a score of 1. All subjects were positive

for HSV-1-IgG in the anti-HSV-1 antibody serological test at baseline.

Safety

During continuous administration at 4×10^8 pfu, OrienX010 had good safety and tolerability.

The overall AE incidence related to study drug decreased with more injections, and the incidence of study drug-related AEs was the highest after the first intratumoral administration. Of all study drug-related AEs, the incidence of fever increased with more injections, while the incidence of most of the rest AEs decreased with more injections.

The enrolled 12 patients (100.0%) received at least 3 doses of study drug and all experienced at least one case of AE. The most common AEs (incidence ≥ 15%) included fever (66.7%), proteinuria (58.3%), injection site reactions (50.0%), white blood cell count decreased (25.0%), vomiting (25.0%), nausea (25.0%), pain of extremities (25.0%), rash (25.0%), neutrophil count decreased (16.7%), peripheral edema (16.7%), and white blood cells urine positive (16.7%). A total of 108 AEs was reported, including 81 grade 1 AEs (75%), 26 grade 2 AEs (24.1%), and 1 grade 3 and above AE (0.9%).

There were 12 patients who reported at least 1 study drug-related AE. During the dosing period, a total of 98 study drug-related AEs was reported, all of which were Grade 1 to 2 in severity, there were no Grade 3 or above study drug-related AEs, and most AEs resolved spontaneously in 1 to 2 weeks without any treatment measure. The most common AEs (incidence ≥ 15%) included fever (66.7%), proteinuria (50.0%), injection site reactions (50.0%), white blood cell count decreased (25.0%), vomiting (25.0%), nausea (25.0%), pain of extremities (25.0%), rash (25.0%), neutrophil count decreased (16.7%), peripheral edema (16.7%), and white blood cells urine positive (16.7%), etc.

No SAEs occurred throughout the treatment period.

The main fluctuation in patient's vital signs was the increase in body temperature at 4 to 48 hours post-dose; blood pressure, respiratory rate, heart rate, and body weight basically remained stable; both pre-dose and post-dose ECOG scores were ≤ 1.

Most of abnormal and clinically relevant laboratory test results and ECG findings that were related or possibly related to study drugs according to the investigator’s judgment were occasional and required for continuous observation in subsequent trials in individual cases.

Efficacy

Of 12 patients, 2 withdrew from the trial midway due to PD and the rest 10 completed 2 treatment cycles (i.e., 12 weeks of treatment) according to study protocol. After completing 2-cycle treatment, 5 patients continued study drug treatment until PD.

According to irRC criteria[13], after study drug treatment, one patient achieved immune-related partial response (irPR), and 6 patients remained immune-related stable disease (irSD), with clinical benefit rate (irPR+ irSD) of 58.3% (7/12). Of all patients obtaining clinical benefit, the average PFS was 26.6 weeks (median: 24 weeks; range: 12 to 54 weeks). The duration of response (DOR) of one patient during OrienX010 treatment reached 42 weeks.

According to Response Evaluation Criteria In Solid Tumours (RECIST) 1.1, one patient

achieved PR, and 4 patients remained SD, with clinical benefit rate (PR + SD) of 41.7% (5/12). Of all patients obtaining clinical benefit, the average PFS was 26.4 weeks (median: 24 weeks; range: 12 to 48 weeks). The DOR of one patient during OrienX010 treatment reached 12 weeks.

Anti-tumor immune response of the drug was observed in the region around injected lesions.

Evaluation of biological distribution and biological effects

As of data cutoff (April 11, 2015), of test indicators related to biological distribution and biological effect, tests of blood lymphocytes and injection site wipe test had been completed and the other tests were ongoing.

Of all 12 patients who received intratumoral injection of OrienX010, OrienX010 activity was not detected in injection site wipe samples within 24h of intratumoral injection.

Average test values of three blood lymphocyte subsets $CD^{4+}CD^{25+}$, $CD^{8+}CD^{28+}$ and $CD^{8+}CD^{28-}$ showed that there were obvious changes in the percentages of lymphocyte subsets of patients before and after administration.

Analysis results of blood cell classification of 3 patients who achieved PR or remained long-term stable disease (SD, PFS > 24 weeks) showed that: ① In the whole treatment period, the percentage of $CD^{4+}CD^{25+}$ subset remained essentially unchanged, between 5% and 10%; ② $CD^{8+}CD^{28+}$tended to increase. For example, the percentage of $CD^{8+}CD^{28+}$ and that of $CD^{8+}CD^{28-}$ of patient 04 inverted obviously following injection of OrienX010 and they were maintained throughout the treatment period, which was consistent with efficacy results (PR); ③ $CD^{8+}CD^{28-}$ tended to decrease. For example, although $CD^{8+}CD^{28-}$ of patient 07 increased obviously on C1D15, its percentage continuously decreased with prolonged treatment.

Conclusions

In this trial, long-term intratumoral administration of OrienX010 had a good safety and a high tolerability, which is consistent with phase 1a clinical study results. Efficacy follow-up was performed in all of the 12 patients until PD. The follow-up period was up to 58 weeks. Seven patients (58.3%) achieved PR or SD during OrienX010 treatment period. Progression-free survival (PFS) of 4 patients (33.3%) was over 24 weeks, PFS of 2 patients (16.7%) was over 36 weeks, and PFS of 1 patient (8.3%) reached 54 weeks.

Considering the maximum dose (4×10^8 pfu) of OrienX010 in the study had a good safety and standard treatment period was short (12 weeks, 1 injection/2 weeks), treatment regimen was adjusted in subsequent clinical trials: ① Increase the injection dose to 8×10^8pfu; ② prolong standard treatment period to 16 weeks (1 injection/2 weeks).

Phase 1b clinical study of OrienX010 (OrienX010-II-09)

"An Open-label Phase 1b Clinical Trial of Recombinant Human GM-CSF Herpes Simplex Virus Injection (OrienX010) Intratumral Injection in the Treatment of Stage IIIb, IIIc, and IV M1a and M1b Malignant Melanoma that Has failed the Standard Treatment Regimen" was initiated in Beijing Cancer Hospital in May 2016 and is ongoing.

This is a single-center, open-label phase 1b clinical trial without any control group to evaluate the safety and efficacy of continuous OrienX010 intratumoral injection in the treatment of stage

IIIb, IIIc, and IV M1a and M1b malignant melanoma patients who had failed the standard treatment, so as to further determine the recommended dose and administration method of phase 2/3 clinical trials.

Patients were treated with OrienX010 (8×10^7 pfu/mL) via intratumoral injection, with the injection volume determined by the size of the tumor lesion. The total volume of each injection will not exceed 10 mL. The frequency of injection is once every 2 weeks, 4 doses per cycle, 2 cycles in total. Upon the completion of efficacy evaluation at the end of Cycle 2, if in the judgment of investigator, the patient can benefit from the continued administration, the study drug can be continued until PD, intolerable toxicity, or patient's withdrawal of informed consent.

Phase 1c clinical study of OrienX010 (OrienX010-II-10)

"An Open-label Phase 1c Clinical Trial of Recombinant Human GM-CSF Herpes Simplex Virus Injection (OrienX010) Intratumral Injection in the Treatment of Stage IV M1c Malignant Melanoma with Liver Metastasis that Has failed the Standard Treatment Regimen" was launched in Beijing Cancer Hospital in December 2016 and is ongoing.

This is a single-center, open-label phase 1c clinical trial without any control group to evaluate the safety and efficacy of continuous OrienX010 intratumoral injection in the treatment of stage IV M1c malignant melanoma patients with liver metastasis who have failed the standard treatment regimen, so as to further determine the recommended dose and administration method of phase 2/3 clinical trials.

Patients were treated with OrienX010 (8×10^7 pfu/mL) via intratumoral injection, with the injection volume determined by the size of the tumor lesion. The total volume of each injection will not exceed 10 mL. The frequency of injection is once every 2 weeks, 4 doses per cycle, 2 cycles in total. Upon the completion of efficacy evaluation at the end of Cycle 2, if in the judgment of investigator, the patient can benefit from the continued administration, the study drug can be continued until PD, intolerable toxicity, or patient's withdrawal of informed consent.

Summary of the phase 3 clinical trial of T-VEC (OncoVex GM-CSF)

This global, randomized, open-label, phase 3 clinical study of T-VEC enrolled more than 400 patients with unresectable stage IIIb/IIIc, and IV malignant melanoma. In the T-VEC group, the study drug was injected into the cutaneous/subcutaneous lesion or metastatic lymph node with a starting dose not more than 4 ml x 10^6 pfu/ml, followed by doses not more than 4 ml x 10^8 pfu/ml three weeks later, once every 2 weeks for continuous administration; the control group was subcutaneously injected with GM-CSF 125 $\mu g/m^2$ once daily for 14 days in 28-day cycles; patients were distributed to the test group and control group at a ratio of 2:1. The primary endpoint was DRR (defined as proportions of CR and PR lasting $\geq$ 6 weeks). The revised WHO criteria were adopted for efficacy evaluation.

436 patients were included in the ITT analysis set, 30% of stage IIIb/IIIc, 27% of stage IV M1a, 21% of stage IV M1b, and 22% of stage IV M1c. ORR of T-VEC group reached 31% (11% reaching CR), while that of the control group was only 6% (1% reaching CR). DRR of T-VEC group reached 19%, while that of the control group was only 2%. From the perspective of

disease staging, DRR (T-VEC, GM-CSF) was 33% VS 0% for stage IIIb/IIIc, 16% VS 2% for stage M1a, 3% VS 4% for stage M1b, and 8% VS 3% for stage M1c, respectively. The median time to response was 4.1 months (1.2 months to 16.7 months). The DOR was long and over 9 months in 68% of patients in T-VEC group. Commonly reported AEs in T-VEC group included asthenia, chills and fever. Important AEs included PD, cellulitis and fever (incidence was 26% in T-VEC group and 13% in the control group). No grade 3 and above AE with an incidence > 3% was observed in any of the two groups.

Of patients with unresectable stage IIIb/IIIc to IV malignant melanoma, there was significant difference in DRR improvement between the group of T-VEC intratumoral injection and the control group (GM-CSF subcutaneous injection), and T-VEC showed to be safe and well tolerated. Interim analysis also revealed the trend of prolonging OS. It had potential efficacy in local and distant metastasis.

The study results were published at the end of 2013. Based on the study results, FDA formally approved the marketing of T-VEC on October 27, 2015.

Summary of Trials on Toripalimab Injection

Clinical studies of toripalimab injection are only carried out in China, including a number of ongoing and planned studies to evaluate the efficacy and safety of toripalimab alone or in combination with other drugs. As of August 31, 2018, a total of 17 phase 1-3 projects are ongoing and have enrolled over 900 patients. The complete list of ongoing studies is as follows:

No.	Study Code	Trial Title	Status
CT1	HMO-toripalimab injection-I-CRP-01	An open-label, single-center, dose-escalation, phase 1 study to investigate the tolerability and pharmacokinetics of single- and multi-dose recombinant humanized anti-PD-1 monoclonal antibody injection in patients with advanced tumor	Enrollment ended
CT2	Toripalimab injection-I-CRP-1.4	A phase 1a clinical study to evaluate the safety, tolerability, pharmacokinetics and pharmacodynamics of single-dose recombinant humanized anti-PD-1 monoclonal antibody injection combined with multi-dose in patients with advanced solid tumors	Enrollment ended
CT3	Toripalimab injection-I-CRP-1.3	A phase 1 clinical study to evaluate the safety, tolerability, pharmacokinetics and pharmacodynamics of single- and multi-dose recombinant humanized anti-PD-1 monoclonal antibody injection in patients with advanced malignant tumors	Enrollment ended
CT4	HMO-toripalimab injection-II-CRP-01	An open-label, multicentre, single-arm, phase 2 clinical study to investigate the efficacy and safety of recombinant humanized anti-PD-1 monoclonal antibody injection in patients with locally advanced or metastatic melanoma who have failed standard treatment	Enrollment ended
CT5	Toripalimab injection-	A multicentre, open-label, phase 1b/2 clinical study	Enrolling

	Ib-CRP-1.0	to evaluate toripalimab injection in treating advanced gastric adenocarcinoma, oesophageal squamous cell carcinoma, nasopharyngeal cancer and head and neck squamous cell carcinoma	
CT6	Toripalimab Injection-I	A phase 1 clinical study to evaluate the safety, tolerability, pharmacokinetics and pharmacodynamics of multi-dose recombinant humanized anti-PD-1 monoclonal antibody injection in patients with relapsed or refractory malignant lymphoma	Enrollment ended
CT7-1	HMO-toripalimab injection-I-PK-01	A phase 1 and parallel control study to compare the similarity of pharmacokinetics and safety of single-dose recombinant humanized anti-PD-1 monoclonal antibody injection in advanced NSCLC patients before and after process changes	Enrollment ended
CT7-2	HMO-toripalimab injection-I-PK-02	A phase 1 and parallel control study to compare the similarity of pharmacokinetics and safety of single-dose recombinant humanized anti-PD-1 monoclonal antibody injection in advanced melanoma patients before and after process changes	Enrollment ended
CT8	HMO-toripalimab injection-II-MM-02	A randomized, controlled, multicentre, phase 2 clinical study to compare recombinant humanized anti-PD-1 monoclonal antibody injection versus high-dose interferon as adjuvant therapy of completely excised mucosal melanoma	Enrolling
CT9	HMO-toripalimab injection-I-CRP-03	An open-label, single-center, dose-escalation, phase 1 study to investigate the tolerability and pharmacokinetics of single- and multi-dose recombinant humanized anti-PD-1 monoclonal antibody injection in patients with advanced triple negative breast cancer	Enrollment ended
CT10	HMO-toripalimab injection-I-CRP-1.4	A prospective, phase 1 clinical study of radiotherapy in combination with recombinant humanized anti-PD-1 monoclonal antibody injection (toripalimab injection) in treating advanced triple negative breast cancer	Enrolling
CT12	HMO-toripalimab injection-II-CRP-02	An open-label, multicentre, single-arm, phase 2 clinical study to investigate the efficacy and safety of recombinant humanized anti-PD-1 monoclonal antibody injection in patients with locally advanced or metastatic urothelial bladder carcinoma who have failed standard treatment	Enrolling
CT13	HMO-toripalimab injection-Ib-CRP-01	An open-label, single-center, dose-escalation, phase 1b clinical study to investigate the tolerability and pharmacokinetics of recombinant humanized anti-PD-1 monoclonal antibody injection combined with axitinib in patients with advanced renal cancer and melanoma who have failed standard treatment	Enrolling

CT14	HMO-toripalimab injection-Ib-NEC-02	A phase 1b clinical study to investigate the safety and efficacy of recombinant humanized anti-PD-1 monoclonal antibody injection in patients with advanced neuroendocrine tumor who have failed standard treatment	Enrolling
CT15	Toripalimab injection-NPC-III	An international, multicentre, randomized, double-blind, phase 3 study to evaluate the efficacy and safety of toripalimab injection/placebo in combination with GP regimen (gemcitabine and cisplatin) in treating advanced nasopharyngeal cancer	Initiating
CT16	Toripalimab injection-016-II-HCC	A randomized, double-blind, multicenter, phase 2 study to evaluate the efficacy and safety of recombinant humanized anti-PD-1 monoclonal antibody (toripalimab injection) as adjuvant therapy of patients after radical resection for high-risk relapsed liver cancer	Initiating
CT17	Toripalimab injection-017-III-MM	A randomized, controlled, multicentre, phase 3 clinical study to investigate recombinant humanized anti-PD-1 monoclonal antibody injection (toripalimab injection) versus dacarbazine as first-line therapy of unresectable or metastatic melanoma	Enrolling
CT18	Toripalimab injection-018-II-LC	A multicentre, single-arm, phase 2 clinical study to evaluate toripalimab injection combined with pemetrexed and carboplatin in treating advanced or relapsed EGFR-sensitive mutatant, T790M-negative non-small cell lung cancer that failed EGFR-TKI treatment	Enrolling

The existing safety and efficacy clinical data of toripalimab injection are mainly from the following 5 clinical trials in patient with solid tumors. Including:

Study HMO-toripalimab injection-I-CRP-01 (CT1) is a phase 1, open-label, single-center, dose-escalation study to investigate the tolerability and pharmacokinetics of single- and multiple-dose toripalimab injection in advanced tumor patients. Dose escalation groups included 1 mg/kg Q2W, 3 mg/kg Q2W, and 10 mg/kg Q2W and a total of 36 patients with solid tumors (mainly including advanced melanoma, renal cancer and urothelial carcinoma) who failed previous treatment were enrolled. No DLT was observed in any dose group. 10 mg/kg was the maximum exposure dose. Effective cases of clinical response were observed in all dose groups.

Study toripalimab injection-I-CRP-1.4 (CT2) is a phase 1a clinical study to evaluate the safety, tolerability, pharmacokinetics and pharmacodynamics of single-dose toripalimab injection combined with multiple doses in patients with advanced solid tumors. Dose escalation groups included 0.3 mg/kg, 1 mg/kg Q2W, 3 mg/kg Q2W, 10 mg/kg Q2W, and 240 mg Q2W and a total of 25 patients with solid tumors (mainly including gastric cancer, oesophageal cancer, and nasopharyngeal cancer, etc) who failed previous treatment were enrolled. No DLT was observed in any dose group. 10 mg/kg was the maximum exposure dose. Effective cases of

clinical response were observed in multiple dose groups.

Study HMO-toripalimab injection-II-CRP (CT4) is an open-label, multicentre, single-arm, phase 2 clinical study to evaluate the efficacy and safety of toripalimab injection 3 mg/kg Q2W in treating patients with locally advanced or metastatic melanoma who failed standard treatment. The primary endpoint was Independent Review Committee-assessed objective response rate (ORR) as per RECIST 1.1. As of January 04, 2018, a total of 128 patients with locally advanced or metastatic melanoma who failed previous treatment were enrolled, and a total of 128 patients were included to SAS. Toripalimab injection showed to be safe and well tolerated. Most adverse reactions were grades 1 - 2. As compared to foreign marketed predicate products, no new, unexpected safety signal has been found.

Toripalimab injection-Ib-CRP-1.0 (CT5) is a phase 1b/2 clinical study in multiple cohorts (basket trial) to preliminarily evaluate anti-tumor effect and tolerability of toripalimab injection in treating advanced gastric adenocarcinoma, oesophageal squamous cell carcinoma, nasopharyngeal cancer and head and neck squamous cell carcinoma, providing basis for subsequent phase 3 clinical study. The primary endpoint was the ORR assessed by the investigator based on RECIST 1.1. Interim analysis as of November 30, 2017 showed that a total of 161 patients were included into the efficacy assessment analysis set. Preliminary data showed that ORR was 22.4%. A total of 201 patients were included into the SAS. The overall safety and tolerability were good and most adverse reactions were grades 1 - 2. No new, unexpected safety signal was observed.

Toripalimab injection-I (CT6) is a phase 1 dose-escalation clinical study to evaluate the safety, tolerability, pharmacokinetics and pharmacodynamics of multi-dose toripalimab injection in patients with relapsed or refractory malignant lymphoma. Dose-escalation groups included 1 mg/kg Q2W, 3 mg/kg Q2W, and 10 mg/kg Q2W. The primary objective was to determine the safety and tolerability of toripalimab injection alone in subjects with previously treated advanced or relapsed malignant lymphoma. Analysis results as of February 23, 2018 showed that a total of 13 patients with lymphoma who failed previous treatment were enrolled. No DLT was observed in any dose group. 10 mg/kg was the maximum exposure dose. Effective cases of clinical response were observed in multiple dose groups.

Clinical trials of toripalimab injection in treating melanoma

HMO-toripalimab injection-II-CRP-01 is an open-label, single-arm, multicentre, phase 2 clinical study to evaluate the safety and efficacy of toripalimab in patients with unresectable or metastatic melanoma who have failed previous systemic treatment. The study excluded patients with ocular melanoma; active autoimmune disorder; previous use of systemic immunosuppressive drug; active metastases to central nervous system; active HIV, HBV or HCV infection; previous use of anti-PD-1 and anti-PD-L1; ECOG PS score of ≥ 2. Patients with BRAF V600E mutation-positive melanoma are not limited to previous use of BRAF inhibitor therapy.

Patients received this product at 3 mg/kg by intravenous infusion once every 2 weeks until PD (investigator-evaluated as per RECIST 1.1 and irRECIST) or unacceptable toxicity. In the first year, efficacy assessment was performed with imaging examination every 8 weeks after the

first dose of study drug; in the second year, assessment was performed every 12 weeks; afterwards, assessment was performed every 16 weeks.

The study enrolled 128 Chinese patients, of whom 127 were included to FAS. The median age was 52.5 years (range: 21 - 76 years). Patients aged ≥ 65 years accounted for 14.2% and males accounted for 45%. Patients with ECOG score of 0 and 1 accounted for 57% and 43%, respectively. Stages III and IV patients accounted for 11% and 89%, respectively. All patients have previously received systemic therapy, of whom 68.5% have previously received ≥ second-line systemic therapy and 44.1% have previously received ≥ third-line systemic therapy. 26.8% of patients reported BRAF mutation, and 20.5% of patients reported PD-L1 positive. Clinical pathological types included cutaneous (non-acral) (22.8%), cutaneous (acral) (39.4%), mucosal (17.3%), and unknown primary site (20.5%). Patients with ocular melanoma were not enrolled.

The primary efficacy endpoint was IRC-assessed ORR) as per RECIST 1.1. The secondary efficacy endpoints were DOR, PFS (investigator-assessed or IRC-assessed as per RECIST 1.1 or irRECIST), ORR (IRC-assessed as per irRECIST, investigator-assessed as per RECIST 1.1 or irRECIST) and OS.

The median follow-up duration of 127 patients was 12.4 months (range: 0.9, 20.5). The last enrolled subject was followed up for at least 12 months. Critical efficacy results are summarized in the table below.

Table of critical efficacy results

**Endpoints**	**Toripalimab 3 mg/kg, once every 2 weeks (N=128)**
**IRC-assessed best overall response as per RECIST 1.1**	N = 127 (IRC-assessed FAS)
Objective response rate (ORR) (%)	22 (17.3%)
(95% CI)	(11.2， 25.0)
Disease control rate (DCR) (%)	73（57.5%）
(95% CI)	(48.4， 66.2)
Complete response (CR)	1（0.8%）
Partial response (PR)	21（16.5%）
Stable disease (SD)	51（40.2%）
**Duration of response (DOR)*, IRC-assessed as per RECIST 1.1**	N=22
Median (month) (95% CI)	NE(12.8, NE)
DOR range	3.7 ~ 14.8+
Percentage of patients with continuous response at month 6 (%)	90.2%
Percentage of patients with continuous response at month 12 (%)	83.7%
**Progression-free survival (PFS)**, IRC-assessed as per RECIST 1.1**	N=127
Median (month) (95% CI)	3.6（2.7， 5.3）
PFS rate at month 6 (%)	36.2%
PFS rate at month 12 (%)	29.9%

**Overall survival****	N=127
Median (month) (95% CI)	NE（16.0, NE）
OS rate at month 6 (%)	87.8%
OS rate at month 12 (%)	69.3%

IRC = Independent Review Committee, NE = not evaluated

FAS = Full Analysis Set, including patients who have received at least one toripalimab therapy and have evaluable baseline lesions (RECIST 1.1), totaling 127. One patient was excluded because pathological biopsy showed pulmonary tuberculosis after enrollment.

*Best overall response includes confirmed CR or PR

** Value based on Kaplan-Meier estimation

Subgroup population analysis

Analysis according to clinical pharmacology subtype:

IRC-assessed ORR as per RECIST 1.1 included: cutaneous (non-acral) 31.0% (9/29, 95% CI: 15.3, 50.8%); cutaneous (acral) 14.0% (7/50, 95% CI: 5.8, 26.7%); mucosal 0% (0/22, 95% CI: 0.0, 15.4); unknown primary lesion 23.1% (6/26, 95% CI: 9.0, 43.7%).

Analysis according to PD-L1 expression status:

Immunohistochemistry was performed to test PD-L1 expression using SP142 antibody. 26 patients were PD-L1 positive (≥ 1% of tumor cells were positive), with ORR of 38.5% (10/26, 95% CI: 20.2, 59.4%); 84 patients were PD-L1 negative, with ORR of 11.9% (10/84, 95% CI: 5.9, 20.8%); 17 patients had unknown PD-L1 status.

Analysis based on BRAF mutation:

86 patients were BRAF wild-type, with ORR of 9.3% (8/86, 95% CI: 4.1, 17.5%); 34 patients were BRAF-mutant, with ORR of 32.4% (11/34, 95% CI: 17.4, 50.5%). 7 patients had unknown BRAF status.

Adverse reactions of toripalimab injection in clinical study of melanoma:

HMO-toripalimab injection-II-CRP-01 is an open-label, multicentre, single-arm, phase 2 clinical study enrolling patients with unresectable or metastatic melanoma who have failed previous systemic treatment. A total of 128 patients received toripalimab 3 mg/kg once every 2 weeks until PD or unacceptable toxicity. Median exposure time of the patients was 4.33 months (range: 1 day to 19.7 months).

Of patients who received treatment with this product, the incidence of all adverse reactions was 97.7%. Adverse reactions with an incidence of ≥ 10% included rash, skin depigmentation, pruritus, anemia, asthenia, hypothyroidism, decreased appetite, fever and cough. The incidence of grade 3 and above adverse reactions was 28.9%. Grade 3 and above adverse reactions with an incidence of ≥ 1% included hypertriglyceridaemia, anemia, hypertension, liver injury, and thrombocytopaenia. The incidence of drug-related SAEs was 11.7%. Drug-related SAEs with an incidence of ≥ 1% included pancreatitis, liver injury, upper gastrointestinal hemorrhage, and thrombocytopaenia.

15.6% of patients discontinued the drug permanently due to adverse reactions. Adverse reactions with an incidence of ≥ 1% included ALT increased, blood creatine phosphokinase

increased, AST increased, pancreatitis, thrombocytopaenia, amylase increased, lipase increased, liver injury and upper gastrointestinal hemorrhage. 7.0% of patients discontinued the drug temporarily due to adverse reactions. Adverse reactions with an incidence of ≥ 1% included ALT increased, hypertriglyceridaemia and rash.

Summary tabulation of adverse reactions (incidence ≥ 5%) and summary tabulation of laboratory test abnormalities (incidence ≥ 10%) in patients who received monotherapy of the product in the study are as follows.

Tabulation of adverse reactions of all grades with an incidence ≥ 5% in HMO-toripalimab injection-II-CRP-01 study* (N = 128)

**Adverse reaction**	**All grades (%)**	**Grade 3-4 (%)**
**Skin and subcutaneous tissue disorders**		
Rash	24.2	0
Skin depigmentation	23.4	0
Pruritus	21.1	0.8
**General disorders and administration site conditions**		
Asthenia	18.8	0
Fever	11.7	0
Pain	5.5	0
**Metabolism and nutrition disorders**		
Decreased appetite	14.8	0
Hyperglycaemia	7.8	0
Hypertriglyceridaemia	5.5	3.1
**Endocrine disorders**		
Hypothyroidism	14.1	0
Hyperthyroidism	6.3	0
**Gastrointestinal disorders**		
Nausea	7.0	0
Constipation	5.5	0
**Respiratory, thoracic and mediastinal disorders**		
Cough	10.9	0
**Blood and lymphatic system disorders**		
Anemia	19.5	2.3
Thrombocytopaenia	7.8	1.6
**Infections and infestations**		
Upper respiratory tract viral infection	9.4	0
**Cardiac disorders**		
Sinus bradycardia	7.0	0
**Musculoskeletal and connective tissue disorders**		
Musculoskeletal pain	6.3	0
Pain of extremities	5.5	0
**Nervous system disorders**		
Dizziness	6.3	0

**Vascular disorders**		
Hypertension	5.5	2.3

*: Adverse reactions are defined as adverse events that are definitely related to, probably related to, possibly related to, unlikely related to study drugs and unevaluable according to the investigator's judgment. Adverse events definitely unrelated to study drug according to the investigator's judgment are excluded.

MedDRA version: 21.0; refer to NCI-CTCAE 4.03; data cutoff: September 15, 2018.

A subject with the same AE more than once has that event counted only once within each SOC and PT.

Note: The following terms represent a group of related events describing a certain clinical symptom other than a single event.

Rash: Including rash, rash generalised, blistery rash, rubella, blister, maculo-papular rash, and dermatitis acneiform

Skin depigmentation: including skin hypopigmentation, skin depigmentation, vitiligo and leukoplakia

Pruritus: Including itching, pruritis, pruritus generalised and pruritus due to rash

Asthenia: Including fatigue and asthenia

Anemia: Including anemia, hemoglobin decreased and red blood cell count decreased

Thrombocytopaenia: including platelet count decreased and thrombocytopaenia

Musculoskeletal pain: including back pain, myalgia, musculoskeletal pain;

Tabulation of laboratory test abnormalities of all grades with an incidence ≥ 10% in HMO-toripalimab injection-II-CRP-01 study (N = 128)*:

Laboratory test abnormality	Incidence of all CTCAE grades (%)	Incidence of CTCAE grades 3 - 4 (%)
**Chemistry**		
ALT increased	30.5	2.3
Blood creatine phosphokinase increased	25.0	1.6
Sugar blood increased	22.7	0.8
AST increased	21.1	1.6
Amylase increased	18.8	3.9
Total bilirubin increased	17.2	0
Bilirubin conjugated increased	17.2	0
**Hematology**		
White blood cell count decreased	21.9	0
Anemia	19.5	2.3
Neutrophil count decreased	16.4	0
**Thyroid function**		
Blood thyroid stimulating hormone increased	29.7	0
Thyroxine free decreased	15.6	0
Blood thyroid stimulating hormone decreased	14.1	0
Thyroxine free increased	12.5	0
Tri-iodothyronine free increased	10.9	0
**Urinalysis**		
Protein urine present	26.6	0

White blood cells urine positive	22.7	0.8
Blood urine present	18.8	0
Red blood cells urine positive	12.5	0

*: Adverse reactions are defined as adverse events that are definitely related to, probably related to, possibly related to, unlikely related to study drugs and unevaluable according to the investigator's judgment. Adverse events definitely unrelated to study drug according to the investigator's judgment are excluded.

Refer to NCI-CTCAE V4.03, data cutoff: September 15, 2018.

2.3 Benefit and Risk Assessment

Risks

No significant safety concerns were identified in the preclinical studies of OrienX010.

Systemic and injection site adverse reactions were observed in phase 1, 2 and 3 clinical trials of foreign predicate products. The former mainly included: mild flu-like symptoms, such as fever, chills, asthenia, nausea, vomiting, headache. In addition, blood pressure decreased, tachycardia, dyspnea, vitiligo, and hepatic enzyme abnormal were observed in individual patients. The latter mainly included injection site skin inflammation, erythema, and fever. Injection site ulcer and vesicle were observed in individual patients. Injection site reactions were mild to severe. Severe reactions mainly occurred following administration at 10^7 pfu to patients with pre-dose serologically negative anti-HSV-1 antibody. Most of the adverse reactions were temporary or transient and reversible.

The systemic and injection site adverse reactions observed in phase 1 clinical studies of OrienX010 were similar to those of foreign predicate products.

Of 24 patients who took part in the phase 1a clinical study, commonly reported study drug-related AEs included: fever (75%), injection site reactions (29.2%), asthenia (25%), headache (12.5%), and peripheral coldness (8.3%), etc. Of 5 SAEs in the whole treatment period, 4 were unrelated to study drug and one was unevaluable.

In the phase 1b clinical trial including 12 patients, the common study drug-related AEs included: fever (66.7%), injection site reactions (50.0%), proteinuria (50.0%), white blood cell count decreased (25.0%), vomiting (25.0%), nausea (25.0%), pain of extremities (25.0%), rash (25.0%), neutrophil count decreased (16.7%), peripheral edema (16.7%), and white blood cells urine positive (16.7%), etc. No SAEs occurred throughout the treatment period.

Safety of toripalimab injection adopted in the study is summarized as follows:

Safety summary of toripalimab injection monotherapy is from 8 single-arm, open-label, single-/multi-center clinical studies. A total of 598 patients were enrolled, including those with advanced melanoma (n = 191), nasopharyngeal cancer (n = 135), esophageal cancer (n = 65), gastric cancer (n = 63), head and neck squamous cell carcinoma (n = 34), non-small cell lung cancer (n = 33), breast cancer (n = 20), lymphoma (n = 24), soft tissue sarcoma (n = 12), urothelial carcinoma (n = 9), renal cancer (n = 6), pancreatic cancer (n = 2), and various other tumors (n = 4). Administration doses of this product were 0.3 mg/kg (n = 3), 1 mg/kg (n = 39), 3 mg/kg (n = 522), 10 mg/kg (n = 31), and 240 mg (n = 3). In the 3 mg/kg group, exposure time of 132 patients (25.3%) was ≥ 6 months and that of 67 patients (12.8%) ≥ 12 months.

The incidence of adverse reactions of all grades of this product as monotherapy was 93.8%. Adverse reactions with an incidence of ≥ 10% included anemia, ALT increased, asthenia, AST increased, rash, fever, blood thyroid stimulating hormone increased, white blood cell count decreased, cough, pruritus, hypothyroidism, decreased appetite, blood glucose increased and blood bilirubin increased (if an AE/SAE is determined to be definitely related, probably related, possibly related or unlikely related by the investigator, causal relationship of the event will be classified as related to study drug). Most adverse reactions were mild and moderate (grade 1 to 2).

The incidence of grade 3 and above adverse reactions was 29.4%. Adverse reactions with an incidence of ≥ 1% included anemia, hyponatremia, infectious pneumonitis, amylase increased, lipase increased, ALT increased, asthenia, AST increased and thrombocytopaenia.

88 patients (14.7%) experienced study drug-related SAEs. The SAEs occurring in ≥ 4 (0.7%) patients included lung infection (n = 10, 1.7%), death (n = 8, 1.3%), pneumonia (n = 6, 1.0%), and platelet count decreased (n = 5, 0.8%). 78 patients (13.0%) experienced study drug-related AEs causing discontinuation of study drug treatment. 52 patients (8.7%) experienced study drug-related AEs causing temporary discontinuation of study drug treatment.

Immune-related adverse reactions were observed in 155 (25.9%) patients, with manifestations of immune-related interstitial lung disease, immune-related hypothyroidism and hyperthyroidism, immune-related pancreatitis, immune related hyperglycaemia or diabetes mellitus, immune-related hepatic function abnormal, and immune-related corticoadrenal insufficiency. Most of these immune-related adverse reactions were grade 1 to 2 and didn't cause discontinuation or interruption of study drug treatment, which completely resolved. These adverse reactions were consistent with those reported for similar products. No new immune-related adverse reaction was observed.

Overall, the AEs observed in the pooled safety data showed that various lab abnormalities were more common, most of which were grade 1-2 in severity, or consistent with the underlying disease profile. Immune-related adverse reactions were consistent with those reported for similar products, without any new safety signals identified, showing good tolerance of the product and controllability of overall AEs.

Benefits

In terms of efficacy, the published phase 2 and 3 clinical study results of the foreign predicate product T-VEC and phase 1 clinical study results of OrienX010 are very promising.

The completed phase 1 studies of OrienX010 indicated: following OrienX010 treatment, of 12 patients with stage IIIb, IIIc and IV M1a malignant melanoma who have failed standard treatment regimen, one achieved irPR and 6 remained irSD as per irRC criteria, with clinical benefit rate (irPR + irSD) of 58.3% (7/12). Of all patients obtaining clinical benefit, the average PFS was 26.6 weeks (median: 24 weeks; range: 12 to 54 weeks). The DOR of one patient during OrienX010 treatment reached 42 weeks.

Although the number of patients treated with the study drug is currently limited, compared to the intended clinical indication and expected efficacy of OrienX010, the above-mentioned systemic and injection site adverse reactions are within an acceptable range.

In addition, OrienX010 alone or combined with toripalimab injection in the study is preferred for advanced melanoma so far.

In summary, it is believed that benefit of the trial outweighs risk.

3 Study Objectives

To evaluate the efficacy and safety of recombinant human GM-CSF herpes simplex virus injection (OrienX010) in combination with recombinant humanized anti-PD-1 monoclonal antibody (toripalimab injection) as neoadjuvant therapy of completely resectable stage III and IV (M1a) melanoma.

3.1 Primary Objectives

To evaluate pathological response rates (pPR, Major PR/Near pCR and pCR rates) and clinical response rate (CR, PR and SD as per RECIST1.1) with recombinant human GM-CSF herpes simplex virus injection (OrienX010) in combination with recombinant humanized anti-PD-1 monoclonal antibody (toripalimab injection) as neoadjuvant therapy of completely resectable stage III and IV (M1a) melanoma.

3.2 Secondary Objectives

To evaluate the clinical response rates (as per iRECIST and iTRECIST), one-year recurrence-free survival (RFS), 2-year RFS, one-year event-free survival(EFS), 2-year EFS, overall survival (OS) and safety of recombinant human GM-CSF herpes simplex virus injection (OrienX010) in combination with recombinant humanized anti-PD-1 monoclonal antibody (toripalimab injection) as neoadjuvant therapy of completely resectable stage III and IV (M1a) melanoma.

4 Study Plan

4.1 Overall Study Design

The study is designed to evaluate the efficacy and safety of recombinant human GM-CSF herpes simplex virus injection (OrienX010) in combination with recombinant humanized anti-PD-1 monoclonal antibody (toripalimab injection) as neoadjuvant therapy of completely resectable stage III and IV (M1a) melanoma.

It plans to enroll 30 patients with completely resectable stage III and IV (M1a) melanoma meeting protocol requirements at a single site Beijing Cancer Hospital.

Patients will enter a 28-day (maximum) screening period after signing written informed consent form (ICF). In the screening period, investigators will evaluate patients' eligibility according to the protocol, including complete medical history, body height, body weight, vital signs, physical examination, ECOG performance status scores, electrocardiogram, laboratory related evaluation, computerised tomography (CT) or magnetic resonance imaging (MRI), and other tumor imaging evaluation.

Subjects will receive preoperative toripalimab injection (Tuoyi®) combined with OrienX010 after enrollment (neoadjuvant treatment period) and will continue toripalimab treatment for up to 1 year after operation (adjuvant treatment period) (the one-year duration will be counted from 1st dose in neoadjuvant treatment).

During neoadjuvant treatment period, patients will receive study visit every 2 weeks. During adjuvant treatment period, patients will receive study visit every 3 weeks until EOT (see section 5.3 for EOT criteria). On day 28 (± 7 days) after the last dose, the patients should receive EOT follow-up. On day 90 (± 7 days) after the last dose, safety follow-up should be performed. See study diagram for specific visit plan.

PD assessment

The primary efficacy measures will be evaluated based on pathological response rates and clinical response rates as per RECIST1.1. At the same time. it is necessary to assess efficacy based on iRECIST and iT-RECIST.

- If patients experience progressive disease (PD) based on RECIST 1.1 at any time, it does not need to evaluate the efficacy based on RECIST 1.1 any more; however, it still requires to assess immune response as per iRECIST criteria until **progressive disease confirmed based on iRECIST (iCPD)**.
- If patients experience **progressive disease unconfirmed based on iRECIST (iUPD)** at any time, it is necessary to confirm in the next 4 - 8 weeks according to iRECIST requirements to judge whether the patients meet iCPD. It can be judged by the investigator according to the actual conditions of patients.
- If a patient meets iCPD, because the injected lesion has response, when the investigator judges it is iT-RECIST-defined progressive disease (iTPD: the investigator considers the patient (if the patient remains clinically stable) can continue benefiting from intratumoral immunotherapy, the sequence of injecting lesions is re-determined and intratumoral

immunotherapy will be continued), intratumoral immunotherapy can be continued and imaging should be performed every 4 - 12 weeks until any one of the following conditions (according to iT-RECIST, the response becomes iCPD):

1. PD related to worsening of clinical status (signs, symptoms or performance status)
2. The investigator and/or patient decides to discontinue treatment due to intolerance
3. Radiological progression, especially in injected lesions (indicating injection cannot block growth), or another treatment (e.g., lesions invade spinal cord, and emergency intervention is required) determined by the doctor based on clinical need

At week 12 of neoadjuvant treatment period and at the end of every 12 weeks during adjuvant treatment period, patients will receive tumor imaging examination and efficacy of anti-tumor therapy will be evaluated according to the above criteria.

Neoadjuvant treatment period (preoperative): OrienX010 + toripalimab injection:

Drug A: **OrienX010** will be intratumorally injected at a total dose of not more than 10 (8.0×10^7pfu/mL, 1 mL/vial, maximum of 10 vials)/dose every 2 weeks. The dose is selected based on tumor size (long diameter): when the long diameter ≥ 5 cm, the dose is 10 mL; when between 2 cm and 5 cm, the dose is not less than 5 mL; when < 2 cm, the dose is not less than 3 mL. Investigators should evaluate the size of injected lesions of patients within 24h before each injection to determine the injection dose of the study drug. Toripalimab injection will be given for 6 doses (6 cycles) after OrienX010 injection (at an interval of more than 60 min)

Drug B: **Toripalimab injection (Tuoyi®)**: 3 mg/kg, IV infusion: Once every 2 weeks for 6 doses (every 2 weeks per cycle; 6 cycles);

Surgical treatment period: The investigator will perform radical resection of melanoma and postoperative care according to individual conditions of subjects.

Adjuvant treatment period (postoperative):

Toripalimab injection (Tuoyi®): Subjects will continue using toripalimab at 3 mg/kg by intravenous infusion every 3 weeks (every 3 weeks per cycle) for up to 1 year (the one-year duration will be counted from 1st dose in neoadjuvant treatment) after surgery. If neoplasm recurrence, metastasis or intolerable AE occurs or subjects withdraw informed consent during the treatment period or there are other situations meeting the criteria for EOT, subjects need to terminate study drug treatment.

4.2 Dose Adjustment Regimen

- **Toripalimab injection**

The dose of toripalimab should not be reduced in the study.

If a subject has a toripalimab related AE, study treatment can be interrupted at the investigator's discretion for no more than 7 days. Or else, it is considered the dose is missing (the subject will receive the next visit according to the original plan (time of the next visit calculated by taking C1D1 as base point)). If a subject has discontinued the drug for over 56 days due to the toripalimab related AE, and the investigator considers the risk of continuing toripalimab therapy outweighs its benefit, EOT will be considered.

Toripalimab therapy may be associated with irAE, such as immune-related hepatitis, pneumonia, colitis, pancreatitis, endocrine disorders (thyroid function decreased, hyperthyroidism, insufficiency adrenal cortex, hyperglycaemia or diabetes mellitus). The investigator should interrupt or terminate toripalimab therapy and/or provide symptomatic treatment, such as steroid therapy, according to the severity of events.

- If subject needs to gradually reduce the dose of steroids for AE, toripalimab can be discontinued for a longer period of time until completing dose reduction of steroids or prednisone dose ≤ 10 mg/day (or equivalent).
- When the drug has been interrupted for more than 56 days, toripalimab can be continued, provided that the subject can clinically benefit from continuing toripalimab at the discretion of the investigator based on overall risk-benefit assessment (the benefit outweighs risk).

The investigator must document the decision of any permanent drug withdrawal.

Please refer to Investigator's Brochure for more information on dose adjustment of toripalimab.

- **OrienX010**

Patients must receive pre-dose evaluation within 24 hours before each dose to determine the dose (see section 7.2.8). If the tumor lesions increase or shrink during the treatment, the dose can be adjusted according to the actual measurement of the lesions prior to each injection. The adjustment principle is that the total dose does not exceed 10 ml.

Patients will receive treatment according to the protocol until: complete response (CR, disappearance of all lesions, only OrienX010), disappearance of all injectable lesions, clinical related progressive disease (PDr), unacceptable toxicity, loss to follow-up, death or situations meeting any EOT criteria (see section 5.3), whichever occurs first.

Definition of PDr: PD related to deterioration of clinical conditions, and/or the investigator considers patients must to change anti-tumor therapy in such situation. Patients can continue administration until PDr to allow the occurrence of "delayed anti-tumor immune response".

Data collection after EOT

EOT visit: Patients need to receive EOT visit on day 28 (± 7 days) after the last dose. Patients who withdraw from the study prematurely will also require EOT visit. In addition, safety follow-up will be performed until 90 days (± 7 days) after the last dose. During safety follow-up period, investigators can collect all study drug-related AEs via outpatient or telephone visit.

Tumor recurrence follow-up: Patients who discontinue treatment for causes other than neoplasm recurrence will receive subsequent neoplasm recurrence follow-up:

- If a patient does not experience neoplasm recurrence during treatment withdrawal, he/she needs to receive subsequent follow-up for tumor assessment until neoplasm recurrence.

Survival follow-up: After PD or neoplasm recurrence, survival follow-up is still needed to collect subsequent anti-tumor therapy and survival information of patients:

- Patients who experience PD or neoplasm recurrence will receive follow-up of survival

status and subsequent anti-tumor therapy every 30 days (± 7 days) via outpatient visit or telephone until death, loss to follow-up, withdrawal of informed consent or EOS, whichever occurs first.

End of study (EOS)

The primary endpoint measure is pathological response rate and will be analyzed at month 12 after the last patient is enrolled or when at least 24 cases of pathological response event have been observed (whichever occurs first). Survival data of patients will continue to be collected after analysis of pathological response. It is planned to end the whole study after survival of 80% of patients has been collected.

If some patients are still using the drugs after EOS, the funder will continue providing the drugs until patients' EOT/death/drug marketing. During continuation of study drug treatment, tumor progression evaluation, survival and drug-related SAEs will be collected as supplemental data.

4.3 Duration of Study

All patients will receive screening assessment during the screening period (within 28 days before the first dose of study drug).

During neoadjuvant treatment period, patients will receive combination therapy of OrienX010 and toripalimab injection according to protocol. During adjuvant treatment period, patients will receive treatment with toripalimab injection according to protocol for up to 1 year (the one-year duration will be counted from 1st dose in neoadjuvant treatment) until occurrence of CR (only OrienX010), PDr, unacceptable toxicity, loss to follow-up, death or meeting other EOT criteria, whichever occurs first;

On day 28 (± 7 days) after the last dose, patients should receive EOT follow-up. Safety follow-up will be performed until 90 days (± 7 days) after the last dose.

If a patient does not experience neoplasm recurrence at EOT, he/she needs to receive subsequent neoplasm recurrence follow-up (see section 4.1) until neoplasm recurrence. After neoplasm recurrence, subsequent anti-tumor therapy information of the patient requires to be collected and his/her survival status will be followed up until death. The whole study will end after survival status of 80% of patients has been collected.

4.4 Discussion of Study Design

The study is designed to evaluate the efficacy and safety of OrienX010 intratumoral injection in combination with recombinant humanized anti-PD-1 monoclonal antibody (toripalimab injection) as neoadjuvant therapy of resectable stage III and IV (M1a) melanoma.

Selection of study population

Study population is patients with resectable stage III and IV (M1a) melanoma.

Selection of co-administered drug

In the study, toripalimab injection is selected to co-administer with OrienX010, because it is a recommended drug for advanced melanoma so far.

Dose selection of OrienX010

According to a phase 1b clinical study of OrienX010 (OrienX010-II-08) in which a maximum dose of 5 mL (4×10^8 pfu) was adopted, long-term intratumoral administration of OrienX010 was safe and well tolerated, which is consistent with phase 1a clinical study results. Considering the maximum dose (4×10^8 pfu) of OrienX010 in the study had a good safety, treatment regimen was adjusted in subsequent clinical trials, that is, the dose was increased to 8×10^8 pfu.

At present, the dose (maximum of 10 mL, i.e., 8×10^8 pfu) has been adopted in study OrienX010-II-09 and study OrienX010-II-10. Study OrienX010-II-09 is to evaluate the efficacy and safety of OrienX010 (at the highest dose of 8×10^8 pfu, intratumoral injection) in patients with stage IIIb, IIIc, IV M1a/M1b malignant melanoma who have failed standard treatment regimen. Study OrienX010-II-10 is to evaluate the efficacy and safety of OrienX010 (at the highest dose of 8×10^8 pfu, intratumoral injection) in patients with stage IV M1c malignant melanoma and metastasis to liver who have failed standard treatment regimen. As of the date of the protocol, the two studies enrolled 20 and 26 patients, respectively and preliminarily demonstrated the safety of the highest dose 8×10^8 pfu. Therefore, 8.0×10^8 pfu is selected in the study.

Efficacy evaluation criteria and period

RECIST 1.1 that has been widely used in clinical practice and drug review is selected as the evaluation criteria for anti-tumor efficacy of study drugs. Meanwhile, iRECIST and iT-RECIST criteria are adopted for evaluation of anti-tumor efficacy of OrienX010. This is because that OrienX010 is an immunotherapy and false progression (temporary enlargement of tumor size, appearance of new lesions) and unique manifestation of immune-related efficacy may occur during immunotherapy. Although RECIST is widely used in clinical practice and drug review, false progression may be classified as PD under RECIST. Taking RECIST 1.1 as the main evaluation criteria for anti-tumor efficacy of study drugs and referring to iRECIST and iT-RECIST criteria can avoid “premature withdrawal of patients who can benefit from immunotherapy” and avoid “excessive treatment of patients who cannot benefit from immunotherapy”. Additionally, the concept of PDr is introduced to more comprehensively and objectively evaluate PD of patients according to clinical manifestations of patients.

In this study, tumor imaging evaluation will be performed once at Week 12 of the neoadjuvant treatment period and every 12 weeks in the adjuvant treatment period. However, the investigator is allowed to carry out the evaluation in a flexible manner based on clinical evaluation of tumor progression. If patients are suspected with PD or response, an unscheduled tumor imaging evaluation will be performed. Therefore, it can avoid “premature withdrawal of patients who can benefit from immunotherapy” and protect patients’ rights and interests, meeting ethical requirements.

5 Study Population

A total of about 30 patients with resectable stage III and IV (M1a) melanoma are required to be enrolled.

5.1 Inclusion Criteria

Patients who meet all of the following criteria can be included in this study:

1. An ICF approved by the Ethic Committee will be voluntarily signed by the patient prior to initiating any screening or specific study procedures;
2. Male or female patients between 18 and 75 years of age;
3. Patients with definite diagnosis of completely resectable stage III and IV (M1a) melanoma based on histology and/or cytology, and at least one measurable lesion;
4. Patients with ECOG performance status of 0 or 1;
5. Expected survival> 4 months;
6. Patients have sufficient organ functions and should meet the following conditions at screening according to reference laboratory range:
   - White blood cell count ≥ 3.0 × 10^9/L;
   - Absolute neutrophil count ≥ 1.5 × 10^9/L;
   - Platelet count ≥ 100 × 10^9/L;
   - Hemoglobin ≥ 90 g/L;
   - Serum albumin ≥ 2.5 g/dL;
   - Liver function: Bilirubin total ≤ 1.5 × upper limit of normal (ULN); alanine aminotransferase (ALT) and aspartate aminotransferase (AST) < 2.5 × ULN;
   - Renal function: Serum creatinine ≤1.5 × ULN or the 24-hour creatinine clearance ≥50 mL/min (Cockcroft and Gault formula);
   - International normalized ratio (INR) ≤ 1.5, and, activated partial thromboplastin time (APTT) or partial thromboplastin time ≤ 1.5 × ULN;
7. Female patients with childbearing potential (including premature menopause, menopausal < 2 years and non-surgical sterilization), male patients, and partners of male patients must agree to use effective contraception during the study: Surgical sterilization, oral contraceptives, intrauterine devices, sexual abstinence or barrier contraceptive combination spermicides; All patients must continue contraception for 6 months after the last treatment.

5.2 Exclusion Criteria

Patients who meet any of the following criteria cannot be included in this study:

1. Patients previously treated with T-VEC or similar drug therapy; patients previously treated with anti-PD-1, anti-PD-L1, anti-PD-L2 drug therapy;

2. Patients with negative anti-herpes simplex virus type I (HSV-1) antibodies IgG and IgM;
3. The patient's lesion does not meet the requirement of the intratumoral injection volume or is not suitable for intratumoral injection;
4. Patients who received anti-herpes simplex virus therapy (such as acyclovir, ganciclovir, valaciclovir, vidarabine) within 4 weeks prior to the first dose of study treatment;
5. Patients who have received another anti-tumor monoclonal antibody (mAb) within 4 weeks prior to the first dose of study treatment or haven't recover (≤ Grade 1) from adverse events due to prior therapy (occurring earlier than 4 weeks);
6. Patients with a history of other (including unknown primary) malignancies within 5 years prior to the first dose of trial treatment. Note: Except for fully treated stage 1 or 2 basal/squamous cell carcinoma of the skin, superficial bladder cancer, or in situ cancer that is treated with potentially curative therapy;
7. Patients with known hypersensitivity to the study drug, its active ingredient, excipients;
8. Patient with HBsAg positive and HBV DNA copies > 1×10^3 copies/mL;
9. Patients with positive hepatitis C virus (HCV) antibodies or human immunodeficiency virus (HIV) antibodies;
10. Patients with any unstable systemic disease, including but not limited to: Serious infection, uncontrolled diabetes mellitus, unstable angina, cerebrovascular accident or transient cerebral ischemia, myocardial infarction, congestive heart failure, and serious arrhythmia, liver, kidney, or metabolic disease requiring medical treatment;
11. Patients with active CNS metastases. Patients may participate in the study if their CNS is adequately treated and their neurological symptoms recover to levels less than or equal to Grade 1 (CTCAE) for at least 2 weeks before enrollment, with the exception of residual signs or symptoms associated with CNS therapy. In addition, patients must be those who do not use corticosteroids or who take stable doses of ≤ 10 mg prednisone/day (or equivalent dose) or who decrease to ≤ 10 mg prednisone/day;
12. Patients with autoimmune disease, received liver or other organs transplantation once before, active pulmonary tuberculosis; or patients received major surgical procedures, live vaccination, immunotherapy within 4 weeks prior to study initiation;
13. Tumor's macrovascular invasion in the iliac and femoral vessels;
14. The disease (e.g., mental illness, etc.) or condition (e.g., alcoholism or drug abuse, etc.) of the patient may increase the patient's risk of receiving trial medication or affect the patient's compliance with the study requirements, or may confuse the study results;
15. Within 30 days of screening, the patient had received any other study product or had participated in another interventional clinical trial;
16. Pregnant or lactating women or women who are preparing for pregnancy or lactation in the study period; men or women who are unwilling to take effective contraception;
17. Other situations unsuitable for enrollment according to the investigator’s judgment.

5.3 EOT and patients’ withdrawal from the study

Criteria for EOT of patients

EOT does not mean that patients will withdraw from the study. Patients should complete subsequent study procedures (see section 4.1 Data Collection after EOT) according to the study protocol after EOT.

The treatment will be discontinued permanently if patients meet any one of the following criteria:

- If the investigator considers that it is in patient's best interests to discontinue study drug due to SAEs or serious clinical laboratory abnormalities, the drug will be discontinued and thefunder will be immediately notified;
- Patients need to use another study drug for the treatment of melanoma for any cause;
- PDr;
- Neoplasm recurrence
- Intolerable toxic effects;
- The interval between two adjacent doses is over 6 weeks for any cause;
- Patients become pregnant or fail to take appropriate contraception (for patients of childbearing potential).

Additionally, if patients’ condition is not improved and/or continues to deteriorate after starting study treatment, study treatment can be discontinued according to the investigator’s judgment. **In the event of PDr, the investigator should obtain the PD confirmed by tumor imaging.** If the investigator considers a patient needs EOT due to clinical progression, the investigator should discuss with the funder’s medical monitor before withdrawal.

Withdraw from study

Patients can withdraw from the study at any time for any reasons without any punishment or compromising medical service in the future.

Withdrawal at the investigator's discretion

It refers to the situations in which an enrolled patient is no longer suitable for continuing the trial, and the study doctor decides that the patient should withdraw from the trial.

1. Safety of patients is influenced due to violation of inclusion or exclusion criteria;
2. Patients’ compliance is poor, influencing efficacy and tolerability evaluation;
3. The investigator requests the patient to withdraw from the study for any medical reasons.

Patient's voluntary withdrawal

1. The patient is unwilling to continue the clinical trial and withdraws the informed consent forms;
2. Lost to follow-up (at least 3 documented attempts to contact before loss to follow-up);

3. The patient dies.

Sponsor's decision of withdrawal

The sponsor stops the patient from participating in the study for medical, safety, regulatory or other reasons according to applicable laws, regulations and Good Clinical Practice.

Handling of withdrawal from study

In any cases, all causes and the leading cause of withdrawal from study, withdrawal date, examinations and their findings at withdrawal should be recorded in the electronic Case Report Form (eCRF). If a patient withdraws from the study prematurely for any reasons, the investigator should make every effort to convince the patient to receive corresponding evaluation and continue follow-up of unresolved AEs:

- If a patient withdraws from the study without neoplasm recurrence, the investigator should make every effort to convince the patient to receive EOT visit and neoplasm recurrence follow-up (section 7.1.4);
- If a patient withdraws from the study during neoplasm recurrence follow-up, subsequent anti-tumor therapy and survival information should be collected via telephone visit, that is, survival follow-up. See section 7.1.5;
- If a patient withdraws from the study/prematurely discontinues study drug treatment due to an AE, the event should be followed up as far as possible until it recovers, becomes stable or resolves to baseline.
- If a patient withdraws consent and is unwilling to provide subsequent information, the patient will not receive any subsequent evaluation or have any additional information collected. The investigator may preserve and continue to use the information collected before withdrawal of consent.

5.4 Study Termination Principle

In the following situations, the investigator will terminate the study and inform EC in writing timely. To ensure the safety of patients, the investigator will be responsible for immediately informing patients of decision of study termination.

- Important safety information is found, such as study drug-related SAE influencing the conduct of the study;
- Other information or causes influencing the conduct of the study.

6 Study Treatment

6.1 Characteristics of Study Drug

Study drugs in the study are OrienX010 and toripalimab injection.

Study drug: Recombinant Human GM-CSF Herpes Simplex Virus Injection (OrienX010). Produced by OrienGene Biotechnology Ltd. Strength: 1.0 mL/vial. Labeled amount: the titer is 8.0×10^7 pfu/mL, and the number of particles is not more than 1×10^{11} VP/mL. Storage condition: Store below -18°C. Shelf life: 30 months. Toripalimab injection. Toripalimab

injection is manufactured by Shanghai Junshi Biosciences Co., Ltd./Suzhou Zhonghe Bio-pharmaceutical Technology Co., Ltd. Storage conditions: Protect from light, store in tight containers at 2 - 8 °C. Shelf life: 24 months.

Table 2 Detailed information of study drug

Study drug	OrienX010	Toripalimab injection
Strength	The titer is 8.0×10^7 pfu/ mL, 1.0 mL/vial	240 mg (6 ml)/bottle, 80 mg (2 ml)/bottle
Excipients	Sodium dihydrogen phosphate, disodium hydrogen phosphate, sodium chloride, sorbitol and inositol	Citric acid monohydrate, sodium citrate dihydrate, sodium chloride, mannitol, and polysorbate 80
Description	It is in a white frozen state at -18°C. After thawing and reconstitution, it is a colorless or off-white suspension liquid without foreign matters or visible particles.	Colorless or light-yellow clear liquid possibly with slight opalescence.
Storage conditions	Store below -18°C	Store and ship at 2 - 8 °C, protect from light, avoid freezing and forceful shaking
Shelf life	30 months	24 months or 36 months
Manufacturer	OrienGene Biotechnology Ltd.	Shanghai Junshi Biosciences Co., Ltd.<br>Suzhou Zhonghe Bio-pharmaceutical Technology Co., Ltd.

*Note: Recombinant human GM-CSF herpes simplex virus injection (OrienX010) is provided by the funder OrienGene Biotechnology Ltd.; recombinant humanized anti-PD-1 monoclonal antibody (toripalimab injection) is donated by Shanghai Junshi Biosciences Co., Ltd./Suzhou Zhonghe Bio-pharmaceutical Technology Co., Ltd.

6.2 Administration Method of Study Drug[12]

Dose Selection

Neoadjuvant treatment period (OrienX010 combined with toripalimab injection)

OrienX010 is administered by intratumoral injection with the maximum injection dose of 8×10^8 pfu, that is, the maximum injection volume of 10 ml (8×10^7 pfu/mL, 1 mL/syringe, maximum amount of 10 syringes). Injection volume is determined based on the size of tumor lesion. Inject once every 2 weeks. A total of 6 treatment cycles.

The dose is selected based on tumor size (long diameter) measured with CT/MRI (if the tumor cannot be measured by CT/MRI, calipers can be used. The investigator can also use B-mode ultrasound for subcutaneous deep lesions if necessary): When the long diameter ≥ 5 cm, the dose is 10 mL; when 2 - 5 cm, the dose is not less than 5 mL; when < 2 cm, the dose is not less than 3 mL.

Toripalimab injection is administered at 3 mg/kg every 2 weeks.

Patients will receive OrienX010 intratumoral injection and intravenous infusion of toripalimab injection every 2 weeks. Toripalimab injection will be given after OrienX010 injection (at an

interval of more than 60 min). The dose of OrienX010 is determined based on tumor size. The total dose per injection does not exceed 10 mL (maximum of 10 vials), that is, 8.0×10^8 pfu. Toripalimab injection (3 mg/kg) will be administered by intravenous infusion within 1 hour for 6 treatment cycles.

Adjuvant treatment period: toripalimab injection

The planned dose of toripalimab injection is: 3 mg/kg, every 3 weeks, by intravenous infusion within 1 hour. Subjects will continue toripalimab therapy after operation for up to 1 year (the one-year duration will be counted from 1st dose in neoadjuvant treatment).

Preparation method of toripalimab injection

Under aseptic conditions, a disposable syringe is used to withdraw the required volume (as per the protocol) of toripalimab injection into a 100 mL normal saline (0.9% sodium chloride solution) infusion bag slowly, the mixed diluent is gently inverted for 3-5 times to avoid forceful shaking. After mixing, use an aseptic, low-pyrogen, low-protein adsorption in-line filter (0.2 or 0.22 μm) for intravenous infusion. The final drug concentration after dilution is 1-3 mg/mL.

Diluent should be prepared within 24 hours after taking this product out of the refrigerator. Visually observe whether there are particles and changes in color of the drug before preparation. This product is colorless or light-yellow clear liquid possibly with slight opalescence. If there are visible particles or color abnormality, do not use the drug. The diluent prepared in an aseptic manner can be stored at room temperature for up to 8 hours, including the storage duration in infusion bag at room temperature and the duration of infusion. It can be stored at 2 - 8 °C for up to 24 hours. In case of cold storage, please let the diluent return to room temperature before administration. Do not store frozen. Do not reuse the remaining drug. Do not use 5% glucose solution. Do not mix or dilute with other medicinal product. Wash the tube with 100 mL of normal saline at the end of infusion.

Administration Method of Toripalimab Injection

The first intravenous dose should be administered for at least 60 minutes. If it is well tolerated, the duration of the second intravenous dose can be shorten to 30 minutes. If it is also well tolerated, all subsequent infusions can be completed within 30 minutes. Do not adopt intravenous push or single rapid intravenous injection.

Dose Adjustment

If the tumor lesions increase or shrink during the OrienX010 treatment, the dose can be adjusted according to the actual measurement of the lesions of the recent CT/MRI (caliper if necessary). The adjustment principle is that the total dose does not exceed 10 ml.

Precautions in Dose Selection and Adjustment

1) For multiple tumor lesions, if the dose allows, several lesions can be selected for injection, but no more than 10 lesions. The lesions for each injection should remain the same, unless the injected lesions are reduced to the extent that they cannot receive injections.

2) Provided that the size and the number of tumor lesions are enough, the maximum dose

should be injected as much as possible.

3) In case of residual drugs due to the shrinkage of the tumor lesions, a new lesion can be selected for injection.

4) For cases with multiple tumor lesions, at least a non-injected lesion should be retained to observe its changes. If the lesions are widely distributed, then local, intraregional, and distal lesions can be retained for observation.

6.3 Precautions of Administration

6.3.1 Precautions of OrienX010 Administration

1) The study drug should be stored frozen. It needs to be reconstituted before injection. After reconstitution, it should be stored at 2-8°C for no more than 8 hours and used the same day, and cannot be refrozen. It can be stored at room temperature for no more than 2 hours. It should be used the same day and cannot be refrozen.

2) No dilution of the study drug is allowed.

3) During the injection, the principle of aseptic operation should be strictly implemented.

4) Generally, single-point insertion of needle is used to avoid spilling the drug from the injection hole as much as possible.

5) Specific administration method of intratumoral injection: Inject OrienX010 into the target tumor using a 10 mL syringe. The maximum injection dose is 10 mL. The specific operation should be completed by an experienced clinician. If the investigator evaluates a patient cannot receive direct intratumoral injection, intratumoral injection can be performed under ultrasound guidance.

6) See Injection Operation Manual of OrienX010 for safety risk control and processing measures of study drugs.

6.3.2 Precautions of Administration of Toripalimab Injection

1) Preventive medication

When subjects experience infusion reaction, it should be managed timely. If grade 2 infusion reaction occurs, this product can be continued under close monitoring or interrupted. Analgesic-antipyretic and anti-inflammatory drugs and antihistamines can be considered for prevention in subsequent therapy. Analgesic-antipyretic (such as acetaminophen) and antihistamines (such as diphenhydramine) can be used for prevention before each subsequent dose to reduce the risk of infusion reaction.

2) Recombinant humanized anti-PD-1 monoclonal antibody injection should not be administered by intravenous push or fast injection. Peripheral or central venous access should be established. Before infusion, sufficient epinephrine for subcutaneous injection, intravenous diphenhydramine hydrochloride or other antiallergic drugs and resuscitation equipment should be prepared for timely managing serious allergic reactions. After infusion, venous access should keep open for administration if necessary. If there is no any complication during 1-hour observation after infusion, venous access can be withdrawn.

Infusion rate

In-line filter (0.2 or 0.22 μm) should be used for intravenous infusion of this drug. The first dose should be intravenously infused for at least 60 minutes. If infusion reaction occurs, the infusion rate may be slowed down or interrupted and necessary treatment may be given until the patient's symptoms improve somewhat, and then the infusion may be continued at half of the previous rate.

3) Monitoring during intravenous infusion

ECG monitoring should be used during each infusion of this product to monitor conditions of subjects. In addition, the first use of ECG monitoring should be under the guidance of experienced doctors. Vital signs (body temperature, respiration, blood pressure and heart rate), complexion of subjects, whether there is sweeting or headache should be closely monitored before and during infusion and within at least 1 hour after infusion, so as to find signs and symptoms of infusion reactions as early as possible.

Some infusion reactions may occur in subsequent administration periods. Even if there is not any infusion reaction of any grade following the first dose, subsequent administration should still be performed under the monitoring of doctors.

For patients with serious infusion reactions, especially patients with serious dyspnea, bronchial spasm, and hypoxaemia, infusion should be stopped immediately. After all symptoms disappear and laboratory test results become normal, the investigator will decide whether to continue infusion; if infusion is resumed, the infusion rate should not be faster than half of the previous rate. If the same SAE reoccurs, drug withdrawal can be considered.

4) Management of infusion reactions

Although antihistamines and glucocorticoids and other drugs for prevention of allergic reaction are used before administration, infusion reaction may still occur during antibody infusion. The investigator can provide timely symptomatic treatment for different grades of infusion reactions according to National Cancer Institute (NCI) Common Terminology Criteria for Adverse Events (CTCAE5.0):

Table 1. Treatment guidelines for infusion reactions

NCI CTCAE grade	Treatment	Pre-treatment in subsequent administration
Grade 1<br>Mild reaction; infusion interruption or treatment intervention is not required	According to medical indications of patients, monitoring of vital signs should be enhanced until the investigator considers conditions of the subjects are stable.	None
Grade 2<br>Infusion interruption is required, but symptomatic treatment (such as antihistamines, NSAIDS, anesthetic, intravenous infusion) should be performed as soon as possible; ≤ 24 hours of	Discontinue infusion and monitor symptoms.<br>Other appropriate drug therapies, including but not limited to:<br>Intravenous infusion<br>Antihistamines	Subjects can receive pre-treatment 1.5 hours (± 30 minutes) before infusion of toripalimab injection. The drugs are as follows:<br>Diphenhydramine 50 mg, per os (or equivalent dose of

preventive administration is required	NSAIDS<br>Acetaminophen<br>Anesthetics<br>According to medical indications of patients, monitoring of vital signs should be enhanced until the investigator considers conditions of the subject are stable.<br>If symptoms of the subject are relieved within 1 hour after stopping infusion, infusion can be resumed at a rate half the previous infusion rate (such as reducing from 100 mL/hr to 50 mL/hr). Or else, the drug should be interrupted until symptom remission. In addition, subjects should receive pre-treatment before the next scheduled administration.<br>For subjects who still experience grade 2 toxicities after sufficient pre-treatment, study treatment should be discontinued permanently.	antihistamines)<br>Acetaminophen 500 - 1,000 mg, per os (or equivalent dose of antipyretic).
Grade 3 or 4<br>Grade 3:<br>Persistent (i.e., failure to have remission rapidly after symptomatic drug therapy and/or temporary discontinuation of infusion); recurrence of symptoms after preliminary improvement; hospitalization required for other clinical sequela (e.g., renal injury, and pulmonary infiltration)<br>Grade 4:<br>Life-threatening; vasopressor or ventilatory support indicated	Infusion discontinued.<br>Other appropriate drug therapies, including but not limited to:<br>Intravenous infusion<br>Antihistamines<br>NSAIDS<br>Acetaminophen<br>Anesthetics<br>Oxygen<br>Vasopressor<br>Corticosteroids<br>Epinephrine<br>According to medical indications of patients, monitoring of vital signs should be enhanced until the investigator considers conditions of the subjects are stable.<br>Hospitalization may be indicated.<br>Subjects should terminate study treatment permanently.	No subsequent administration
Appropriate first-aid equipment should be prepared in ward and doctors should be present during administration.		

5) Treatment adjustment regimen:

According to the safety and tolerability of individual patients, temporary or permanent discontinuation of toripalimab injection may be required. It is not suggested to increase or reduce the dose. See the table below for guidance of temporary or permanent discontinuation of administration:

Immune-related adverse reactions	Severity*	Treatment adjustment regimen
Pneumonia	Grade 2	Dose interruption until recovered to grade 0-1
	Grades 3-4, or recurrence grade 2	Permanent drug withdrawal
Diarrhea and colitis	Grade 2-3	Dose interruption until recovered to grade 0-1
	Grade 4	Permanent drug withdrawal
Hepatitis	Grade 2, aspartate aminotransferase (AST) or alanine aminotransferase (ALT) = 3 - 5 × upper limit of normal (ULN)or bilirubin total = 1.5 - 3 × ULN	Dose interruption until recovered to grade 0-1
	Grade 3 - 4, AST or ALT > 5 × ULN, or bilirubin total > 3 × ULN	Permanent drug withdrawal
Nephritis	Grade 2 - 3 blood creatinine increased	Dose interruption until recovered to grade 0-1
	Grade 4 blood creatinine increased	Permanent drug withdrawal
Endocrine disorders	Grade 2 - 3 symptomatic thyroid function decreased, grade 2 - 3 hyperthyroidism, grade 2 - 3 hypophysitis, grade 2 adrenal gland insufficiency<br>Grade 3 hyperglycaemia or type I diabetes mellitus	Dose interruption until recovered to grade 0-1
	Grade 4 thyroid function decreased<br>Grade 4 hyperthyroidism<br>Grade 4 hypophysitis<br>Grade 3-4 adrenal gland insufficiency<br>Grade 4 hyperglycaemia or type I diabetes mellitus	Permanent drug withdrawal
Cutaneous adverse reaction	Grade 3 rash	Dose interruption until recovered to grade 0-1
	Grade 4 rash<br>Stevens-Johnson syndrome (SJS) or toxic epidermal necrolysis (TEN)	Permanent drug withdrawal
Thrombocytopaenia	Grade 3	Dose interruption until recovered to grade 0-1
	Grade 4	Permanent drug withdrawal
Other	Grade 2-3 blood amylase increased or lipase increased	Dose interruption until recovered to grade 0-1

	Grade 2 pancreatitis<br>Grade 2 myocarditis[a]<br>Grade 2 - 3 other immune-related adverse reactions occurring for the first time	
	Grade 4 blood amylase increased or lipase increased<br>Grade 3-4 pancreatitis<br>Grade 3-4 myocarditis<br>Grade 3 - 4 encephalitis<br>Grade 4 other immune-related adverse reactions occurring for the first time	Permanent drug withdrawal
**Recurrent or persistent adverse reactions**	Recurrent grade 3 - 4 (except for endocrine disorder)<br>Grade 2 - 3 adverse reactions do not recover to grade 0 - 1 within 12 weeks after the last dose (except for endocrine disorder)<br>Corticosteroid is not reduced to ≤ 10 mg/day prednisone equivalent dose within 12 weeks after the last dose	Permanent drug withdrawal
**Infusion reactions**	Grade 2	Reduce infusion speed or interrupt administration. The drug can be resumed after symptoms are recovered and close attention should be paid
	Grade 3-4	The drug must be discontinued immediately and permanently and symptomatic treatment should be provided

6.3.3 Continued administration

Patients will receive treatment according to the study protocol until:

- CR of patients (disappearance of all lesions, only OrienX010);
- Complete disappearance of all injected lesions (only OrienX010);
- Occurrence of PDr;
- Meeting any EOT criteria (see section 5.3);
- The investigator believes it is necessary to change to other anti-tumor therapy to ensure patients' maximum benefit. In this context, any efficacy of treatment or clinical evidence of bioactivity must be recorded in eCRF, including tumor shrinkage, flattening, necrosis, erythema, vitiligo, inflammation of one or more tumors, etc. Any conditions that have an impact on quality of life or patient's compliance (e.g., side effect of treatment, lack of anti-tumor effect, patient's unwillingness to comply with study requirements, such as going to study site every 2 weeks for injection) and on decisions of treatment withdrawal should be indicated in eCRF;

- Withdrawal of informed consent by the patient.

6.4 Criteria for interruption/permanent discontinuation of OrienX010 and toripalimab injection

6.4.1 Criteria for interruption/permanent discontinuation of OrienX010 therapy

1. If a grade 4 AE occurs, OrienX010 therapy should be discontinued permanently.
2. If a study drug related grade 3 AE occurs, OrienX010 therapy should be interrupted until the AE resolves to CTCAE ≤ grade 1 or baseline status; if the AE does not recover to CTCAE ≤ grade 1 or baseline status in the maximum administration interval (6 weeks) permitted by the protocol, OrienX010 therapy should be discontinued permanently.
3. If the investigator considers it is necessary to interrupt OrienX010 therapy based on the subject's AE, laboratory test abnormality or other information, OrienX010 therapy can be interrupted until the investigator considers it can be resumed. The interval between two doses should not be more than the maximum administration interval (6 weeks) specified in the protocol.

6.4.2 Criteria for interruption/permanent discontinuation of toripalimab injection

Refer to section 6.3.2 Treatment Adjustment Plan.

Note: If it can be confirmed that an AE is caused by one of two combined drugs, the drug causing the AE can be discontinued temporarily/permanently. If it cannot be confirmed, both drugs should be discontinued temporarily/permanently.

6.5 Package and Label of the Study Drug

The funder and/or drug donating party will package the study drugs according to local regulatory requirements and paste labels according to applicable regulatory requirements.

The drug labels should have a uniform format with the contents including: clinical trial approval number, protocol number, name of the clinical study drug (indicating it is for clinical trial), dosage and administration, strengths, packaging specifications, storage, batch number, date manufactured, shelf life, Sponsor and/or the drug donating party, etc.

A complete record of all study drug batch numbers and expiration dates as well as drug labels will be maintained in the study file folder.

6.6 Storage and Management of Study Drugs

Distribution of Study Drug

The study drugs will be provided by the funder and/or drug donating party and distributed to study site as planned. The designated personnel of study site should examine transportation temperature of study drugs, count the drugs, and sign after verifying and receiving the drugs. Relevant records should be preserved in ISF. Meanwhile, drug storage temperature should be recorded every day and contents of inventory form should be recorded: including date of receiving study drugs, quantity, batch number, distributed quantity and inventory of study drugs.

During the study period, study group members will distribute the drugs. The members should

ensure patients receive the drugs as planned and record the quantity of drugs distributed and returned and date of distribution and return in the original medical record.

The investigator should use the study drugs in the frame of clinical study according to protocol requirements.

Storage of Study Drug

Study site should strictly comply with the requirements of "Managing Investigational Products" in GCP, preserve and manage study drugs according to the storage conditions required by the funder and/or drug donating party.

OrienX010 must be stored below -18°C (inclusive). Toripalimab injection must be stored in tightly containers at 2 - 8 °C, protected from light. Study drugs must be stored in a safe region before dispensed to patients and only authorized personnel have an access to the drugs.

Destruction and Returning of Study Drug.

The study drugs should be destroyed and returned according to relevant standard operating procedures (SOPs) of study site. The investigator should ensure all study drugs are only used in subjects who take part in the clinical trial. Residual drugs after opened should be destroyed. Unopened drugs should be returned to the funder and/or drug donating party. Study drugs cannot be transferred to any non-clinical trial participant.

Clinical research associate (CRA) should be responsible for monitoring the supply, use and storage of the clinical study drugs and the disposal of the remaining drugs.

6.7 Handling of Clinical Events

In the study, patients will be provided with all necessary supportive therapies. The investigator will determine whether a patient continues treatment according to potential risk/benefit assessment.

6.8 Concomitant Medications and Supportive Therapies

All previous medications/concomitant medications (including allowed and prohibited concomitant medications) and treatments from day 28 before screening to EOT visit should be recorded in the original medical record and eCRF, including the following information on concomitant medications: generic name, route of administration, start date, end date and indication. Any dose adjustment to concomitant medications or changes in treatment regimen should be recorded in the eCRF.

If an AE occurs during the study period, all patients should obtain adequate supportive treatment for the AE. All drugs and supportive treatments used in the study period, including start and end date and instruction, should be recorded in the eCRF and source documents of patients. If the supportive treatment has been used at the beginning of study, it can be continued in the study period. This section specifies the concomitant medications and supportive treatments allowed and prohibited to be used.

Drugs Allowed to be Used

Based on the judgment of the investigator, the following drugs are allowed during the study

period when necessary:

- Antiemetics
- Antidiarrheal
- Antiallergic measures
- Erythrogenin, erythrogenin-like substance, blood or platelet transfusion
- Broad-spectrum antibiotics - broad-spectrum antibiotics for suspected or confirmed infections can be used
- Oral or systemic dose ≤ 10 mg/day prednisone or other equivalent dose of steroids (low-dose systemic steroids injected into joint space, such as triamcinolone acetonide lactone)
- Other necessary drugs at the investigator’s discretion

Prohibited concomitant medications and therapies

- Other concurrent chemotherapy, radiotherapy, targeted therapy or other anti-tumor drugs are not allowed during participation in this study. Any other form of anti-tumor therapy will result in the patient's premature termination of the study;
- Palliative radiotherapy to symptomatic sites where the disease develops is not allowed during the study. Any form of required radiotherapy (including palliative radiotherapy) willresult in the patient's premature termination of the study;
- In principle, preventive auxiliary drugs are not allowed in the study period, including white blood cell increasing drug and immune function enhancer; but the investigator can provide preventive drugs for persistent AEs (such as fever, nausea and pain) during administration and make records strictly;
- High-dose steroids, except for oral and systemic steroids allowed to be used above;
- Antiviral drugs are prohibited before entering the adjuvant treatment period (including but not limited to ganciclovir, valganciclovir, cidofovir, adefovir, foscarnet, zidovudine, Lamivudine, stavudine, didanosine, zalcitabine, delavirdine and saquinavir);
- Systemic corticosteroids and other immunosuppressive medications should not be used before starting toripalimab injection because they may interfere with pharmacodynamic activity of other drugs. However, to treat immune-related adverse reactions, systemic corticosteroids and other immunosuppressive medications can be used after starting treatment with this product.

6.9 Study Compliance

To ensure treatment compliance, the dose will be monitored by the investigator or his/her assistant. Accurate medication time and dose should be recorded in the eCRF. Delayed administration, dose reduction, dose missing and causes for termination should also be recorded in the eCRF. Compliance will be further confirmed according to study drug dispensing, drug preparation, return, study case and eCRF.

Any conditions that have an impact on patient’s compliance (e.g., side effect of treatment, lack

of anti-tumor effect, patient's unwillingness to comply with study requirements, such as going to study site every 2 weeks for intratumoral injection or intravenous infusion) and on decisions of treatment withdrawal should be indicated in eCRF.

Patients' compliance with treatment or protocol refers to voluntary compliance with various aspects of protocol, including compliance with various examinations for safety assessment and imaging examinations for tumor assessment. If a patient does not receive visit on time, his/her participation in the study can be discontinued according to the opinions of the principal investigator or the funder.

6.10 Randomization

Not applicable. The study adopts a single-arm design.

6.11 Blinding

Not applicable. The study adopts an open-label design.

7 Study Procedures

Visits and assessment will be carried out according to schedule of activities (Table 1).

7.1 Schedule of Study Visits

7.1.1 Screening Visits (D-28 to D-1)

After the study protocol is approved by the Ethics Committee of the study site, patients with stage III and IV (M1a) melanoma will be screened. First, they will read the "Informed Consent Form" for the study and have enough time to consider. If they decide to participate in the study, they will sign the written Informed Consent Form and complete screening visit within 28 days before starting study treatment. The following study procedures should be completed at screening visit to ensure patients are eligible for taking part in the study:

- Signing the Informed Consent Form
- Recording demographic data (including date of birth, gender, race)
- Past medical history, including the following contents:
  - Recording diagnosis history and date of melanoma
  - Prior treatment history of melanoma (including surgical treatment, radiotherapy, chemotherapy, hormone therapy, immunotherapy and other therapies)
  - Tumor progression within 6 months before signing the Informed Consent Form
  - Concomitant disease
  - History of smoking, alcoholic use, and allergy.
  - Surgery and trauma history (prior surgery history includes significant operation, such as gastrointestinal endoscopy, biopsy and other diagnostic or therapeutic non-invasive procedures)
- Prior/concomitant medications within 28 days before signing the Informed Consent Form
- Body height and body weight measurement (body weight is expressed in the unit of kg and accurate to one decimal place)
- Physical examination
- Vital signs (including body temperature, pulse, blood pressure and respiration)
- ECOG performance status score
- 12-lead ECG
- Tumor TNM staging
- Laboratory test (hematology, blood chemistry, urinalysis, coagulation function and thyroid function)
- Blood pregnancy test (women of childbearing potential. Female patients of childbearing potential include women with early menopause, menopause < 2 years and non-surgical sterilization)

- Virus serology test: HBsAg, number of HBV DNA copies, HCV antibody and HIV antibody
- Serological test of anti-HSV-1 antibody: Anti-HSV-1 IgG and anti-HSV-1 IgM
- Tumor tissue biopsy: Live tumor tissue biological samples should be collected in the screening period
- Blood sample retention for biomarker test: Blood samples should be collected in the screening period for exploratory study
- Tumor imaging examination: Whole-body PET-CT scan and CT/MRI scan of all other clinically indicated sites will be performed within 28 days before randomization. The PET-CT and CT/MRI scans before signing ICF are acceptable for those within 28 days before enrollment, which don't need to be repeated unless the investigator considers tumor burden of the patient has been changed.
- Checking the inclusion/exclusion criteria;
- Recording AEs.

7.1.2 Treatment Period

Baseline visit (C1D1)

Patients who meet inclusion criteria upon screening should complete the following procedures before the first dose:

- Body weight measurement
- Physical examination
- ECOG performance status score
- ECG examination (ECG examination does not need to be repeated at baseline if its screening examination is within 7 days before baseline, unless the investigator considers it is necessary to receive examination again based on patients' conditions)
- Vital signs (body temperature, pulse, blood pressure and respiration)
- Laboratory tests (hematology, blood chemistry, and urinalysis: If the above tests have been performed within 7 days before the first dose, screening results will be taken as baseline data)
- Pre-dose evaluation (to be completed within 24h pre-dose)
- Photography and caliper measurement of superficial lesions
- Recording concomitant medications
- Recording AEs

After completing the above examinations and records, the investigator will:

- Checking the inclusion/exclusion criteria again

Patients will receive the first dose of study drug:

- Intratumoral injection of OrienX010 in combined with intravenous infusion of toripalimab
- Recording AEs

Study visits

Neoadjuvant treatment period: OrienX010 + toripalimab injection

Patients will receive OrienX010 + toripalimab injection therapy every 2 weeks.

Patients need to complete all of the following examinations and procedures on the day of administration (CnD1 ± 3 days)

- Body weight measurement: Before administration
- Vital signs: Before administration
- Physical examination: Before administration
- ECOG performance status score: Before administration
- Pre-dose evaluation: to be completed within 24h pre-dose
- Laboratory tests (hematology, blood chemistry, urinalysis, 12-lead ECG): Within 48 hours before administration
- Thyroid function: At week 12 of treatment
- Blood sample retention for biomarker test: At weeks 6 and 12 of treatment, before adjuvant therapy
- Tumor biopsy: At week 6 of neoadjuvant therapy, and additional biopsy will be performed at the discretion of the investigator and with consent of the patient. Blood coagulation function should be tested before each biopsy and biopsy samples will be collected and sent to the pathological department for test
- Blood coagulation function: Within 7 days before tumor tissue biopsy (only when tumor tissue biopsy is required)
- Cotton swab sampling (in case of cold sores or other suspected lesions caused by herpes virus after injection of OrienX010, wiping and sampling with cotton swab should be performed)
- Photography and caliper measurement of superficial lesions: Before administration
- Tumor imaging examination: Whole-body PET-CT scan and CT/MRI scan of all other clinically indicated sites will be performed at week 12 of treatment. The investigator can perform additional imaging examination according to the conditions of subjects.
- Study drug administration: Intratumoral injection of OrienX010 in combined with intravenous infusion of 3 mg/kg toripalimab injection
- Recording concomitant medications
- Recording AEs

Surgical treatment period

The investigator will perform radical resection of melanoma and postoperative care according to individual conditions of patients. Subjects' specimens will be collected during operation and the specimens will be processed according to relevant requirements. Pathological image reading personnel of the study site will carry out routine pathological examination and report, and evaluate pathological response.

Adjuvant treatment period
: toripalimab injection

Patients will receive toripalimab injection therapy every 3 weeks.

Patients need to complete all of the following examinations and procedures on the day of administration (CnD1 ± 3 days)

- Body weight measurement: Before administration
- Vital signs: Before administration
- Physical examination: Before administration
- ECOG performance status score: Before administration
- Laboratory tests (hematology, blood chemistry, urinalysis, 12-lead ECG): Within 48 hours before administration
- Blood sample retention for biomarker test: Before adjuvant therapy
- Thyroid function: Every 9 weeks, within 48 hours before administration
- Photography and caliper measurement of superficial lesions: Before administration
- Pre-dose evaluation: to be completed within 24h pre-dose
- Tumor imaging examination: CT/MRI scan of the chest, abdomen, pelvic cavity and all other clinically indicated sites will be performed every 12 weeks of treatment. The investigator can perform additional imaging examination according to the conditions of subjects.
- Cotton swab sampling (in case of cold sores or other suspected lesions caused by herpes virus after injection of OrienX010, wiping and sampling with cotton swab should be performed)
- Study drug administration: 3 mg/kg toripalimab injection
- Recording concomitant medications
- Recording AEs

Tumor assessment

The primary efficacy measure will be evaluated and recorded based on pathological response rates and efficacy as per RECIST 1.1. Meanwhile, for patients who receive treatment, efficacy should be evaluated and recorded based on RECIST 1.1, iRECIST and iT-RECIST criteria[14]. See section 4.1 for details.

At week 12 of neoadjuvant treatment period and at the end of each 12-week adjuvant therapy,

patients will receive tumor imaging examination (see section 7.2.9) and efficacy of anti-tumor therapy will be evaluated according to the above criteria for evaluation of progressive disease in section 4.1. If the investigator considers it is necessary to have additional tumor imaging evaluation based on clinical evaluation results to clarify PD or response, unscheduled imaging evaluation can be arranged.

HSV-1 antibody test

Serum anti-HSV-1 neutralizing antibody of patients will be tested before treatment and at EOT visit to explore the relationship between neutralizing antibodies and efficacy.

Tumor tissue biopsy and blood sample collection for biomarker test

In addition to live tumor tissue biological samples collected in the screening period, additional biopsy will be performed at week 6 of neoadjuvant therapy at the discretion of the investigator and with consent of the patient. During surgery, the investigator should collect corresponding tumor tissue samples of subjects to support the exploratory study in the future. Blood coagulation function test should be performed before each collection of tumor tissue specimens. If the investigator considers the patient's coagulation function meets the requirements, tumor tissue specimens can be collected.

Moreover, the investigator needs to collect blood samples in the screening period, at weeks 6 and 12 of neoadjuvant therapy and before adjuvant therapy to support the exploratory study in the future.

In case of neoplasm recurrence after lesion resection, the investigator can collect live tumor tissue biological samples and blood samples for biomarker test on a case-by-case basis.

See section 7.2.11 for study evaluation operations.

7.1.3 EOT visit (day 28 ± 7 days after the last dose)

Patients need to return to study site for EOT visit on day 28 (± 7 days) after the last dose. Patients should complete all of the following examinations and evaluations at visit:

- Body weight measurement
- Physical examination
- Vital signs
- ECOG performance status score
- ECG test
- Laboratory tests: hematology, blood chemistry, urinalysis coagulation function and thyroid function
- Blood pregnancy test: Women of childbearing potential only, including women with early menopause, menopause < 2 years and non-surgical sterilization
- HSV-1 antibody test
- Tumor imaging evaluation: CT/MRI scan of the chest, abdomen, pelvic cavity and all other clinically indicated sites will be performed. The investigator can perform additional

imaging examination according to the conditions of subjects. If patients have received the above evaluation within 28 days before the visit, it does not need to repeat. If patients discontinue treatment due to PD or neoplasm recurrence, tumor imaging evaluation will not need at EOT visit.

- Photography and caliper measurement of superficial lesions
- Cotton swab sampling (in case of cold sores or other suspected lesions caused by herpes virus, wiping and sampling with cotton swab should be performed)
- Recording concomitant medications
- Recording AEs (safety follow-up will be performed until 90 days (± 7 days) after the last dose. During safety follow-up period, investigators can collect all study drug related AEs via outpatient visit or telephone visit)

7.1.4 Follow-up of neoplasm recurrence

Patients who discontinue treatment for reasons other than neoplasm recurrence will receive neoplasm recurrence follow-up according to the study drug used. See description of neoplasm recurrence follow-up in section 4.1.

The following procedures should be completed during the follow-up:

- Physical examination
- Vital signs
- ECOG performance status score
- Laboratory tests: hematology, blood chemistry, urinalysis
- Tumor imaging evaluation: CT or MRI examination, at an interval as described above. If patients have received the above evaluation within 28 days before the visit, it does not need to repeat.
- Photography and caliper measurement of superficial lesions
- Recording subsequent anti-tumor therapy and survival status

7.1.5 Survival follow-up (every 30 days ± 7 days after EOT)

Patients who experience PD or neoplasm recurrence will receive follow-up of survival status and subsequent anti-tumor therapy every 30 days (± 7 days) via outpatient visit or telephone until patient’s death, loss to follow-up, withdrawal of informed consent or EOS, whichever occurs first.

7.1.6 End of Study

The primary endpoint measure is pathological response rate and will be analyzed at month 12 after the last patient is enrolled or when at least 24 cases of pathological response event have been observed (whichever occurs first). Survival data of patients will continue to be collected after PFS analysis. The whole study plan will end after survival of 80% of patients has been collected.

funder and/or drug donating partyIf some patients are still using OrienX010 and/or toripalimab injection after EOS, the funder and/or drug donating party will continue providing such drug until these patients cannot benefit from the treatment any more (at the discretion of investigator)/death/drug marketing. During continuation of the drug, information about tumor progression evaluation, survival and drug-related SAEs will be collected as supplemental data.

7.2 Study Assessment

7.2.1 Physical examination

The investigator will carry out physical examination of patients according to schedule of activities (Table 1). Examination contents include: General condition, skin, head, neck, ear, eye, nose, mouth, throat, respiratory organ/lung, cardiovascular, gastrointestinal tract/abdomen, urogenital system, lymphatic system, muscle and bone/extremities, nervous system and other.

Abnormal physical examination findings with clinical significance at the investigator's discretion will be recorded as an AE.

7.2.2 Height and Weight

All patients will have height measured only during screening examination. Coat and shoes should be taken off when measuring body weight. Body weight should be measured before each administration of toripalimab injection to determine administration dose. All patients should have weight measured at EOT visit.

7.2.3 Vital signs

Vital signs will be evaluated according to schedule of activities (Table 1). Evaluation contents include: Body temperature, pulse, blood pressure (systolic and diastolic blood pressure) and respiration.

Patients should have systolic and diastolic blood pressure measured on the same arm after rest for 5 minutes in supine/sitting position. Pulse and blood pressure will be measured at the same time.

During the study period, vital sign examination frequency can be increased at the investigator's discretion. Abnormal values with clinical significance will be recorded as AEs.

7.2.4 12-lead ECG

ECG examination will be performed according to schedule of activities (Table 1). Patients should receive the examination after rest for 5 minutes in supine position. The following ECG parameters should be recorded: Heart rate, PR interval, QRS duration, QT interval and QTc interval evaluation and investigator's judgment of ECG curves.

At screening, the investigator or the personnel designated by the investigator will evaluate ECG finding to determine whether it is normal or abnormal. If abnormal, it is necessary to judge whether the abnormality is clinically significant or not and whether the patient can still be enrolled. ECG examination does not need to be repeated at baseline if its screening examination is within 7 days before baseline, unless the investigator considers it is necessary to receive examination again based on patients' conditions.

All ECGs should be evaluated by qualified doctors. The investigator should record any clinically significant findings as AEs.

7.2.5 ECOG performance status score

ECOG performance status will be scored at screening, before each dose and at EOT visit. See Appendix I for the criteria of ECOG performance status score.

7.2.6 Laboratory Tests

Laboratory tests will be carried out in the laboratories of each study sites. Samples will be collected and analyzed according to local laboratory requirements. The following laboratory data will be tested:

- **Hematology:** Red Blood Cell (RBC), hemoglobin (Hb), white blood cell (WBC), neutrophil percentage (N%), lymphocyte percentage (L%), platelets (PLT), monocyte percentage (M%), eosinophil percentage (E%), basophil percentage (B%), hematocrit (HCT), mean corpuscular volume (MCV), mean cell hemoglobin (MCH), and mean cell hemoglobin concentration (MCHC);
- **Blood chemistry:** Alanine aminotransferase (ALT), aspartate aminotransferase (AST), total bilirubin (TBIL), direct bilirubin (DBIL), total protein (TP), albumin (ALB), alkaline phosphatase (ALP), electrolyte (K, Na, Cl), creatinine (Cr), uric acid (URIC), fasting plasma glucose (FPG), urea (UREA), creatine kinase (CK), lactate dehydrogenase (LDH), total cholesterol (TC), triglycerides (TG), high density lipoprotein cholesterol (HDL-C), low density lipoprotein cholesterol (LDL-C), and gamma glutamyl transpeptidase (GGT).
- **Urinalysis:** Urine glucose (GLU), urine protein (PRO), urine ketone body (KET), red blood cells urine (RBC), white blood cells urine (WBC), bilirubin urine (BIL), urine specific gravity (SG), power of hydrogen (pH), urobilinogen (URO), and nitrite urine
- **Blood coagulation function** (test in the screening period and before each collection of tumor tissue specimen): International normalised ratio (INR), activated partial thromboplastin time (aPTT) or partial thromboplastin time (PTT)
- **Virus serology test** (test in the screening period only): HBsAg, number of HBV DNA copies (not required if HBsAg is negative), HCV antibody and HIV antibody
- **Blood pregnancy test:** All female patients of childbearing potential will receive blood pregnancy test in the screening period and at EOT visit.
- **Thyroid function:** total thyroxine (TT4), total tri-iodothyronine (TT3), free thyroxine (FT4), free tri-iodothyronine (FT3), and thyroid stimulating hormone (TSH). Thyroglobulin antibody (TGAb) can be tested when necessary.

During the study period, for safety concerns, the investigator can increase clinical laboratory test frequency and items. Moreover, serological test of HSV-1 antibody will be carried out in the central laboratory (Adicon Clinical Laboratories, LTD):

- **Serological test of anti-HSV-1 antibody:** Anti-HSV-1 IgG and anti-HSV-1 IgM

7.2.7 Cotton Swab for Wiping and Sampling

In the whole study process, in case of cold sores or other suspected lesions caused by herpes virus at any time following injection of OrienX010, wiping and sampling with cotton swab should be performed.

7.2.8 Pre-dose Evaluation

Patients should receive pre-dose evaluation within 24 hours before each dose to determine injection dose of study drug. OrienX010: Long diameter will be measured with callipers and recorded. Subcutaneous deep lesions can be estimated with B-mode ultrasound and their long diameter should be recorded, at the discretion of the investigator. According to CT/MRI measurements (lesions that cannot be measured with CT/MRI will be measured with callipers. Subcutaneous deep lesions will be estimated using B-mode ultrasound, at the discretion of the investigator), injection dose of each lesion and total injection dose will be calculated with the longest diameter of lesions and records will be made; toripalimab injection: administration dose will be calculated based on body weight of patients.

7.2.9 Tumor Radiographic Images

Whole-body PET-CT scan and CT/MRI scan of all other clinically indicated sites will be performed within 28 days before randomization. The PET-CT and CT/MRI scans before signing ICF are acceptable for those within 28 days before enrollment, which don't need to be repeated unless the investigator considers tumor burden of the patient has been changed. At week 12 of neoadjuvant treatment period, whole-body PET-CT scan and CT/MRI scan of all other clinically indicated sites will be performed. During adjuvant therapy, head (if necessary), chest, abdomen, pelvis CT/MRI scan and CT/MRI scan of all other clinically indicated sites will be performed every 12 weeks and the assessment method consistent with that in the screening period/baseline period will be adopted until CR (only OrienX010), PDr or PD, intolerable toxicity, loss to follow-up, death, withdrawal of informed consent or EOS, whichever occurs first. If clinically indicated, bone scan should be performed. If the investigator considers it is necessary to have additional tumor imaging evaluation based on clinical evaluation results to clarify PD, neoplasm recurrence or response, unscheduled imaging evaluation can be arranged.

7.2.10 Photography and caliper measurement of superficial lesions

Superficial lesions will be photographed and measured with calipers in the screening period, before each dose and at EOT visit. That is, superficial lesions larger than 10 mm in diameter that cannot be examined by CT or MRI will be measured with calipers. In case of skin lesions, colorful photos will be taken as records and photos will indicate size and proportion of lesions measured. If there are multiple lesions, the lesions should be numbered. If the lesions can be clinically measured and radiographically examined, CT or MRI must be performed at the time point of tumor imaging evaluation.

During clinical measurement and recording, whether there are signs of efficacy with drug therapy should be observed particularly: for example, whether there is tumor shrinkage, flattening, necrosis, erythema, vitiligo, inflammation of one or multiple tumors, etc.

Photography and archive requirements of color digital photos: Color digital photos of all visible tumor lesions should be obtained at screening visit, before each dose and at EOT visit. Photos

of four extremities should be taken and archived at screening to record the overall disease status at baseline. All photos should be indicated with study number of patients and photography date and keep patients anonymous. All photos should include the same ruler as reference, so that lesion size can be evaluated by other reading personnel. The investigator can take photos of lesions and archive the photos as appropriate at any time of the study. In the event of vitiligo or any other obvious clinical changes, photos should be taken at each subsequent treatment visit until resolution.

7.2.11 Tumor tissue and blood sample collection for biomarker test

In addition to live tumor tissue biological samples collected in the screening period, additional biopsy will be performed at week 6 of neoadjuvant therapy at the discretion of the investigator and with consent of the patient. During surgery, the investigator should collect corresponding tumor tissue samples of subjects to support the exploratory study in the future. Blood coagulation function test should be performed before each collection of tumor tissue specimens. If the investigator considers the patient's coagulation function meets the requirements, tumor tissue specimens will be collected. In the screening period, biopsy should be performed unless patients can provide 30 unstained slices or paraffin-embedded block of live tumor tissues. See "Operation Manual for Tumor Tissue Biopsy in Project OrienX010-II-12" for tumor specimen requirements and SOP for biopsy.

Moreover, the investigator needs to collect blood samples in the screening period, at weeks 6 and 12 of neoadjuvant therapy and before adjuvant therapy to support the exploratory study in the future. For biomarker test, about 20 mL of blood will be collected with 4 whole blood tubes and 1 serum collection tube.

In addition, in case of recurrence after lesion resection, the investigator can collect live tumor tissue biological samples and blood samples for biomarker test on a case-by-case basis.

Blood samples for biomarker test will be temporarily stored at Geneplus-Beijing Clinical Laboratory Co., Ltd. that is entrusted by the Internal Medicine Department of Renal Cancer and Melanoma, Beijing Cancer Hospital for later biomarker test and analysis. A high-throughout gene sequencing platform will be adopted for target region deep sequencing of all exon regions of 312 genes, introns of 38 genes, promoters or breakpoint regions of fusion gene, and coding regions of 709 genes of samples, detection of four variation types of tumor genes. Results are given a full score upon External Quality Assessment of the Ministry of Heath. Therefore, the platform is accurate and comprehensive and provide comprehensive and credible test results.

8 Efficacy Evaluation

8.1 Efficacy Measures

Primary efficacy measure

- Pathological response rate: Pathological response will be evaluated according to the INMC score system. Pathological response can be classified into pathological partial response (pPR, active tumor cells ≤ 50%), near pCR (Major PR/Near pCR, 0% < active tumor cells < 10%), pathological complete response (pCR, no active tumor cells).
- Clinical response rate: Clinical response rate will be evaluated based on RECIST1.1. It is defined as the percentage of patients whose tumor volume shrinkage meets the predetermined value and lasts for the minimum time limit, including CR and PR cases.

Secondary efficacy measures

- Clinical response rate: Clinical response rate will be evaluated as per iRECIST and iT-RECIST.
- 1-year recurrence-free survival (RFS) and 2-year RFS after administration: The duration from starting administration to relapse; if relapse does not occur within 1 year, 1 year will be taken as censoring time. For subjects lost to follow-up within 1 year, the last visit date will be used for censoring. If relapse does not occur within 2 years, 2 years will be taken as censoring time. For subjects lost to follow-up within 2 years, the last visit date will be used for censoring.
- To evaluate the safety of OrienX010 combined with toripalimab injection as neoadjuvant therapy in patients with completely resectable stage III and IV (M1a) melanoma.

To observe surgery related events.

- Overall survival (OS) 2 years after administration: The duration from starting administration to death; if death does not occur within 2 years, 2 years will be taken as censoring time. For subjects lost to follow-up within 2 years, the last visit date will be used for censoring.

8.2 Efficacy Evaluation Methods

The primary efficacy measure will be evaluated based on pathological response rates determined by pathologist as per International Neoadjuvant Melanoma Consortium (INMC) scoring system. Meanwhile, for patients who receive treatment, efficacy should be evaluated and recorded based on RECIST 1.1, iRECIST and iT-RECIST criteria[14]. See section 4.1 for details. See Appendix II for RECIST 1.1.

Methods of tumor measurement

Method of tumor measurement is as follows, including: tumor imaging assessment, photograph and measurement of superficial lesions with calipers. If there are indications for tumor metastases to lymph nodes, color Doppler ultrasound of superficial lymph nodes can be performed. Tumor measurement method should be consistent with that at baseline throughout the study.

Imaging evaluation (CT or MRI): the evaluation method and technical parameters of PET-CT, CT or MRI should be consistent throughout the study (CT slice thickness does not exceed 5 mm); contrast media should be used if there is no contraindication. The evaluation conducted with same method and technical parameters at the same site within 28 days prior to the first administration will be seen as tumor evaluation at baseline. Tumor evaluation should include head, thorax, abdomen, pelvis and any other sites with suspected tumor lesions. **Photography and measurement of superficial lesions with calipers:** Superficial lesions larger than 10 mm in diameter that cannot be examined by CT or MRI will be measured with calipers. In addition to measurement with calipers, color digital photos of skin lesions should be taken as records and be filed. See section 7.2.10 for specific requirements. If the lesions can be clinically measured and radiographically examined, imaging methods must be performed at the time point of imaging evaluation.

Injected lesions and non-injected lesions during neoadjuvant treatment period (OrienX010 + toripalimab injection)

Injected lesion: The lesion where the study drug is injected;

Non-injected lesions: Non-injected lesions needing to be observed include the following three categories:

1. Local non-injected lesions: Non-injected lesions within 2 cm diameter around the injected lesions;
2. Regional non-injected lesions: Non-injected lesions within 2 cm diameter around the injected lesions in the same anatomical region. For example, the injected lesion is a right forearm nodule, so the lesions in the same side of acra and the same side axillary range are regional lesions;
3. Distant non-injected lesions: Non-injected lesions in a different anatomical region from injected lesions. For example, the injected lesion is a right forearm nodule, the lesions in the contralateral upper limb, both lower limbs or the trunk are distant lesions.

Photography and measurement with calipers: At baseline, before each dose, and when achieving PDr. All skin lesions selected as injected lesions and non-injected lesions needing observation should be photographed and measured with calipers. See section 7.2.10.

9 Adverse Events

9.1 Definition of Adverse Events

An adverse event (AE) refers to any untoward medical occurrence in a patient who takes part in a clinical study which does not necessarily have a causal relationship with this treatment. Therefore, an AE can be any adverse and unexpected signs (including abnormal laboratory findings), symptoms, or diseases that have a temporal relationship with the use of study drug, regardless of whether it is considered to be related to the study drug.

A serious adverse event (SAE) is defined as any untoward medical occurrence that meets any of the following conditions at any dose:

- Result in death
- Life-threatening: It refers to an AE in which the patient was at risk of death at the time it occurs; it does not refer to an AE which hypothetically might have caused death if it were more severe
- Result in hospitalization or prolongation of existing hospitalization
- Result in permanent or significant disability or dysfunction
- Congenital deformity/birth defect
- Important medical event: The event may not be immediately life-threatening or cause death or require hospitalization, but may damage patients, or need appropriate measures to avoid the occurrence of any of the above listed events.

Severity

Severity of AEs will be evaluated as per CTCAE 5.0 as follows:

- Grade 1: mild; asymptomatic or mild symptoms; clinical or diagnostic observations only; intervention not indicated
- Grade 2: moderate; minor, local or non-invasive intervention indicated; limiting age-appropriate instrumental activities of daily living (ADL) (instrumental ADL refers to preparing meals, shopping for groceries or clothes, using the telephone, managing the money, etc.)
- Grade 3: severe or medically significant, but not immediately life-threatening; hospitalization or prolongation of hospitalization indicated; disabling; or limiting self-care activities of daily living
- Grade 4: life-threatening, urgent intervention indicated
- Grade 5: death related to AE

Relationship between AEs and study drug

Relationship between study drugs and AEs or the role of study drugs in AEs (see Table 3):

Table 3 Evaluation and analytical method of correlation with ARs

Correlation	Criteria
Related	• There is a reasonable temporal sequence between the occurrence of AE and the medication;<br>• The study drug explains the AE more reasonably than other reasons (e.g., the patient's preexisting disease, environmental or toxic factors, or other treatments the patient received, etc.);<br>• The AE disappeared or relieved with reduced dose or after drug interruption;<br>• The AE meets the known AE types of the suspect drug or its similar drugs;<br>• The AE recurs after re-administration.
Possibly related	• There is a reasonable temporal sequence between the occurrence of AE and the medication;<br>• The study drug explains the AE as reasonably as other reasons (e.g., the patient's preexisting disease, environmental or toxic factors, or other treatments the patient received, etc.);<br>• The AE disappears or lessens after the drug is stopped or the dose is reduced (if applicable).
Unlikely related	• Other reasons (e.g., the patient's preexisting disease, environmental or toxic factors, or other treatments the patient received, etc.) explain the AE more reasonably than the study drug;<br>• The AE does not disappear or lessen after the drug is stopped or the dose is reduced (if applicable), or the situation is unclear;<br>• The AE does not recur after re-administration, or the situation is unclear.
Not related	• There is no reasonable temporal sequence between the occurrence of AE and the medication, or;<br>• The AE has other evident reasons (e.g., the patient's preexisting disease, environmental or toxic factors, or other treatments the patient received, etc.).
Indeterminable	• The above information is unclear, and the investigator believes that it cannot be judged based on the existing information, and the investigator cannot obtain further follow-up information.

Abbreviation: AE=adverse event

9.2 Report of Serious Adverse Event

In case of a SAE during the clinical trial, regardless of whether it is related to administration, the investigator should follow the requirements of relevant regulatory departments or the Ethics Committee.

The investigator should take the following measures immediately:

- Immediately provide appropriate treatments for the patient;
- The investigator should record all available information of the event in the initial report after awareness of the event; the investigator should fill in and submit follow-up report after obtaining follow-up information within the same time limit; the investigator should also immediately report any latest significant follow-up information of the event to the EC and the funder of drugs; see “Contact Information Form for Reporting Serious Adverse

Events" for contact information;

- Report to drug funder as soon as possible and sign and date the report;
- Record SAEs in the column of serious adverse events of AE form of eCRF and the original documents;
- The investigator needs to preserve the original AE reporting form and proof of successful report (such as return receipt of successful fax).

Moreover, the drug funder should report suspected and unexpected serious adverse reactions to regulatory authorities and other investigators according to the requirements of regulatory authorities and local regulations.

9.3 Handling and Follow-up of AEs

The investigator should seriously monitor relevant AEs, evaluate the seriousness, severity, and causality of each AE. All AEs/SAEs occurring during the study period should be followed up until:

- Resolution of AE,
- Abnormal laboratory test values recover to baseline or acceptable stable level according to the evaluation of the investigator and medical monitor,
- Observed AE is explained with satisfaction (e.g., the AE is caused by the patient's underlying disease),
- Loss to follow-up or death of patient.

9.4 Adverse Events of Special Interest

Adverse events of special interest (AESIs) refer to clinically significant or general AEs needing higher alert and special management. If the following adverse reactions occur in the study period, they should be handled according to the following rules.

9.4.1 AESIs of OrienX010

According to previous study experience of OrienX010, immune-related AESIs may occur during the study period and is likely to influence patients' treatment compliance, leading to drop out of patients. AESIs of toripalimab injection are suspected immune-related myocarditis: Cardiac enzymes increased accompanied by electrocardiogram change or clinical symptom of hepatic function abnormal meeting Hy's Law criteria:

ALT or AST increased (> 3 × ULN) accompanied by bilirubin total increased (> 2 × ULN) or clinical jaundice, excluding obstructive jaundice or bilirubin increased for other reasons

It should be noticed that whether there is tumor shrinkage, flattening, necrosis, erythema, vitiligo, inflammation of one or multiple tumors, etc. In this context, it is necessary to explain to the patient that these are the manifestations caused by study drugs to improve patients' treatment compliance.

9.4.2 AESIs of toripalimab injection

9.4.2.1 Allergic Reactions

Antibody administration may cause allergic reactions. Therefore, immediate provision of appropriate medications and medical equipment to treat acute allergic reactions is crucial, and study staff must be trained to recognize and treat allergic reactions. The study site must be equipped with emergency rescue team and equipment, as well as the ability to admit subjects to intensive care unit if necessary. In case of severe allergic reaction, the subject must be given immediate emergency treatment according to the relevant local medical routine. Subjects must be given immediate epinephrine and dexamethasone and immediate ECG monitoring, and consideration may be given to drawing serum IgE specimens. Subjects must be immediately and permanently discontinued from study treatment. The investigator must be informed immediately if such symptoms occur.

The National Institute of Allergy and Infectious Diseases (NIAID) and Food Allergy and Allergy Network (FAAN) Anaphylaxis Network guidelines define an allergic reaction as a serious allergic reaction with rapid onset and potential for death (Sampson et al. 2006). Allergic reactions should be highly suspected in patients exposed to allergens if any of the following 3 conditions occurs:

1. Acute onset (minutes to hours) of allergic reaction involving skin, mucosal tissue, or both (e.g., generalized urticaria, pruritus or flushing, swollen lips and tongue), and at least one of the following conditions occur:
   a) Dyspnea (e.g., dyspnea, stridor-bronchospasm, stridor, decreased peak expiratory flow (PEF), hypoxemia);
   b) Decreased blood pressure (BP) or symptoms associated with end-organ dysfunction (e.g., hypotensive shock, syncope, incontinence).
2. After exposure to an allergen (minutes to hours), patients may experience two or more of the following:
   a) Mucocutaneous involvement (e.g., generalized urticaria, pruritus, swollen lips and tongue);
   b) Dyspnea (e.g., dyspnea, stridor-bronchospasm, stridor, decreased PEF, hypoxemia);
   c) Decreased blood pressure or associated symptoms (e.g., hypotensive shock, syncope, incontinence);
   d) Persistent gastrointestinal symptoms (e.g., abdominal pain, vomiting).
3. Decreased blood pressure of the patient after exposure to a known allergen (minutes to hours):
   a) Infants and children: low systolic pressure (age-specific) or less than 30% decrease in systolic blood pressure;
   b) Adults: Systolic blood pressure less than 90 mm Hg or more than 30% less than the subject's baseline.

9.4.2.2 Infusion Reactions

Use of any recombinant protein may produce infusion reactions. It may be due to recombinant protein-mediated immune cell activation, such as NK cells, dendritic cells, or cytokines induced through Fc segment.

Clinical symptoms of infusion reactions include fever, chilliness, chills, headache, rash, pruritus, arthralgia, hypotension or hypertension, and bronchospasm, etc.

For prompt treatment of infusion-related reactions, necessary medications should be prepared before starting the infusion and be placed under a routine of easy access to bedside and management of infusion-related reactions.

In the event of symptoms of infusion reaction (e.g., chilliness, chills, fever, cervicodynia, nausea), the infusion should be interrupted and the subject should be assessed to determine whether there are other signs or symptoms that suggest a more severe reaction (e.g., hypotension, hypoxia, wheezing, urticaria). Monitor vital signs every 15 minutes until symptoms disappear.

Medications used to treat infusion reactions will be given by the investigator according to local best medical routine. If a severe reaction occurs, ECG monitoring and rescue medication (including but not limited to epinephrine, corticosteroids, antihistamines, bronchodilators, and oxygen) may be required. In the event of infusion reaction or suspected event with CTCAE Grade 2 or higher, infusion of toripalimab injection must be stopped immediately. For the first intravenous drop of toripalimab injection, the infusion should be administered over 60 minutes using an in-line filter (0.2 or 0.22 μm). If infusion reaction occurs, the infusion rate may be slowed down or interrupted and necessary treatment may be given until the patient's symptoms improve somewhat, and then the infusion may be continued at half of the previous rate. Patients are also monitored for vital signs within 30 minutes (±5 minutes) after the end of infusion. Patients will be informed of possible delayed post-infusion symptoms and asked to contact their study doctor if they experience the above symptoms. See the table below for treatment adjustment guidelines for infusion reactions. If a CTCAE Grade 2 or above infusion reaction recurs, the patient must be immediately and permanently discontinued from the study.

CTCAE Grade	Adjustment Measures
Grade 1 - mild	For transient mild reaction, interruption of infusion and clinical intervention are not recommended. Decrease the dripping speed by 50% and observe closely for any worsening symptoms. Clinical intervention is performed when necessary.
Grade 2 - moderate	Suspend the treatment of toripalimab injection and immediately administer systemic therapy (e.g., antihistamines, NSAID, narcotics, intravenous fluid replacement); perform re-administration and decrease its dropping rate by 50% when infusion reaction resolves to Grade 0-1. During this period, closely observe any worsening symptoms. Adopt appropriate therapeutic intervention according to local medical routine.
Grade 3 - serious	Infusion discontinued immediately and the infusion tube disconnected. The investigator should discuss with the study funder or medical CRA of CRO whether re-administration should be performed according to the actual situation of patients [a].<br>If the drug is resumed and at the subsequent treatment, the infusion should be continued for at least 2 hours with appropriate prophylactic medication (e.g., antihistamines and NSAIDs), and the patient should be closely observed for clinical symptoms of associated infusion reactions.<br>Adopt appropriate therapeutic intervention according to local medical routine.
Grade 4 - life-threatening and urgent clinical intervention indicated	Patients with Grade 4 infusion reactions must be immediately and permanently discontinued from the study. Adopt appropriate therapeutic intervention according to local medical routine.

Note: a: If patients who can still benefit from toripalimab injection therapy completely recover from infusion reaction, toripalimab injection therapy can be continued. Toripalimab injection therapy can be resumed after the investigator discusses with medical monitor based on overall risk-benefit assessment and the medical monitor approves and make records.

9.4.2.3 Immune-related Adverse Events (irAEs)

Since toripalimab injection can cause T cell activation and proliferation, immune-related adverse reactions may occur during the clinical study period, including severe and fatal cases. Immune-related adverse reactions may occur during and after toripalimab therapy and may involve any tissue and organ.

Optimal strategies and experience for monitoring immune-related toxicities are accumulating, therefore, subjects receiving toripalimab injection should be carefully monitored for signs and symptoms of irAEs. In the absence of alternative etiologies (e.g., infection or progressive disease), signs or symptoms of all inflammation-related events such as myocarditis, enterocolitis, pneumonia, dermatitis, hepatitis and endocrine disorder should be considered as immune-related. Although most immune-related adverse events observed with immunomodulators are mild and self-limiting, these events should be recognized early and treated promptly to avoid potential major complications. Discontinuation of toripalimab injection may not have an immediate therapeutic effect, and in severe cases, immune-related toxicities may require topical corticosteroids, systemic corticosteroids, mycophenolate mofetil, or tumor necrosis factor inhibitors.

Suspected immune-related adverse reactions should be fully evaluated to exclude other causes. Most immune-related adverse reactions are reversible and can be handled by interruption of toripalimab, corticosteroid therapy and/or supportive treatment. Generally, drug interruption is required for most grades 3 - 4 and some specific grade 2 immune-related adverse reactions. Permanent drug discontinuation is required for grade 4 and some specific grade 3 immune-related adverse reactions. For grades 3 - 4 and some specific grade 2 immune-related adverse reactions, 1 - 2 mg/kg/day of prednisone or an equivalent dose and other therapies should be given until resolved to ≤ grade 1. The dose of corticosteroid should be gradually reduced for at least 1 month until withdrawal, and fast dose reduction may cause deterioration or recurrence of adverse reactions. If an adverse reaction continues deteriorating or is not relieved after corticosteroid treatment, non-corticosteroid immunosuppressant therapy should be increased.

The general management guidelines summarized in the table below will provide guidance to investigators regarding management and recommended interventions to prevent morbidity and mortality from these rare immune-related toxicities. Toxicities related or possibly related to toripalimab injection treatment should be managed according to standard medical practice.

1	Thorough assessment of the subject to identify any alternative etiology
2	In the absence of a clear alternative etiology, all inflammatory events should be considered immune-related
3	Managed according to severity and system organ
4	Early intervention, early, adequate systemic corticosteroid therapy is critical (e.g., for persistent low-grade events or serious events, consider prednisone or intravenous equivalents)
5	If the symptoms are not relieved within 2-3 days after initiation of corticosteroid treatment, the dose may be increased and be gradually reduced after the symptoms relieved to Grade 0 or 1, and the

	dose reducing process must be long enough (longer than 4 weeks, or even 6 to 8 weeks) to avoid recurrence of immune-related adverse events, especially immune-related pneumonia and hepatitis;<br>If corticosteroid is reduced to ≤ 10 mg/day of prednisone or a lower dose, toripalimab injection therapy can be resumed;<br>If an irAE recurs, toripalimab injection therapy should be discontinued permanently.
6	In case of suboptimal response to systemic corticosteroid therapy, more effective immunosuppressants - TNF antagonists (e.g., infliximab) or cyclophosphamide, intravenous gamma globulin, or mycophenolate mofetil should be considered after discussion with medical monitor

Refer to NCCN Guidelines Version 1.2018 Management of Immunotherapy-Related Toxicities

The drug should be discontinued permanently if a grade 3 immune-related adverse reaction recurs after toripalimab injection therapy, a grade 2 - 3 immune-related adverse reaction does not resolve to grade 0 - 1 (except for endocrine disorder) within 12 weeks after the last dose, and corticosteroid is not reduced to ≤ 10 mg/day of prednisone or equivalent dose within 12 weeks after the last dose.

Immune-related Pneumonia

Immune-related pneumonia was reported in patients (1.8%, 11/598) who received toripalimab, including death. If immune-related pneumonia occurs, patients should be closely monitored for symptoms (e.g., dyspnea, hypoxia), signs and imaging examination (e.g., local ground glass appearance change, patchy infiltration) and other possible causes should be excluded. If grade 2 immune-related pneumonia occurs, this product should be interrupted. In the event of grade 3 - 4 or recurrent ≥ grade 2 immune-related pneumonia, this product should be discontinued permanently.

Immune-related Diarrhea and Colitis

Immune-related diarrhea was reported in patients who used toripalimab (0.2%, 1/598). Whether patients had diarrhea and other intestinal inflammation symptoms should be monitored, such as abdominal pain and mucous stool. Infection and underlying disease should be excluded. If grade 2 - 3 immune-related colitis occurs, this product should be interrupted. In the event of grade 4 immune-related colitis, this product should be discontinued permanently.

Immune-related Hepatitis

Immune-related hepatitis was reported in patients who received toripalimab (3.5%, 21/598), including death. Patients should be monitored regularly (every month) for changes in liver function and corresponding symptoms and signs of hepatitis, and infection and underlying disease should be excluded. In the event of immune-related hepatitis, liver function test frequency should be increased. If grade 2 immune-related hepatitis occurs, this product should be interrupted. If grade 3 - 4 immune-related hepatitis occurs, this product should be discontinued permanently.

Immune-related Nephritis

Immune-related nephritis was reported in patients who was treated with toripalimab (0.8%, 5/598) . Patients should be monitored regularly (every month) for changes in renal function and corresponding symptoms and signs of nephritis. In the event of immune-related nephritis,

renal function test frequency should be increased. Most patients with raised serum creatinine do not have clinical symptoms. Other causes for renal injury should be excluded. In the event of grade 2 - 3 blood creatinine increased, this product should be interrupted. In the event of grade 4 blood creatinine increased, this product should be discontinued permanently.

Immune-related Endocrine Disorders

Hyperthyroidism and hypothyroidism

Dysfunction thyroid was reported in patients who used toripalimab, including hyperthyroidism (4.8%, 29/598), hypothyroidism (12.9%, 77/598) and thyroiditis. Patients should be closely monitored for changes in thyroid function and corresponding clinical symptoms and signs. In the event of grade 2 - 3 symptomatic hypothyroidism, this product should be interrupted and thyroid hormone replacement therapy can be started if necessary. In the event of grade 2 - 3 symptomatic hyperthyroidism, this product should be interrupted and antithyroid drug can be given if necessary. If acute thyroiditis is suspected, this product can be interrupted and hormone therapy can be provided. If symptoms of hypothyroidism or hyperthyroidism are improved and thyroid function recovers, this product can be resumed according to clinical need. In the event of life-threatening hypothyroidism or hyperthyroidism, this product should be discontinued permanently. Thyroid function will continue to be monitored to ensure appropriate hormone replacement treatment is adopted.

Hyperglycaemia or type I diabetes mellitus

Hyperglycaemia or type I diabetes mellitus were reported in patients who received toripalimab therapy (2.8%, 17/598). Patients should be closely monitored for blood glucose level and relevant clinical symptoms and signs. Insulin replacement therapy can be given according to clinical need. For type I diabetes mellitus with poorly controlled blood glucose, this product should be interrupted and insulin replacement therapy should be given until the symptoms are relieved. For life-threatening grade 4 type I diabetes mellitus, this product should be discontinued permanently. Blood glucose will continue to be monitored to ensure appropriate insulin replacement treatment is adopted.

Adrenal insufficiency

Immune-related adrenal insufficiency was reported in patients who received toripalimab therapy (0.3%, 2/598). Patients should be closely monitored for symptoms and signs of adrenal cortex insufficiency. For symptomatic grade 2 adrenal insufficiency, this product should be interrupted and physiologic corticosteroid replacement therapy should be provided according to clinical need until the symptoms are relieved. For grade 3 - 4 adrenal insufficiency, this product should be discontinued permanently. Adrenal function and hormone level will continue to be monitored to ensure appropriate corticosteroid replacement treatment is adopted.

Hypophysitis

Immune-related hypophysitis was reported in patients who used toripalimab (0.2%, 1/598). Patients with hypophysitis should be closely monitored for symptoms and signs (including hypopituitalism and secondary adrenal insufficiency), and other causes should be excluded. For grade 2 - 3 symptomatic hypophysitis, this drug should be interrupted and hormone

replacement therapy should be provided according to clinical need. If acute hypophysitis is suspected, corticosteroid therapy should be provided. For life-threatening grade 4 hypophysitis, this product should be discontinued permanently. Adrenal function and adrenocortical hormone level will continue to be monitored to ensure appropriate corticosteroid replacement treatment is adopted.

Immune-related cutaneous adverse reactions

Immune-related cutaneous adverse reactions were reported in patients who received toripalimab (3.2%, 19/598). For grade 1 - 2 rash, this product can be continued, and symptomatic treatment or local corticosteroid should be provided. For grade 3 rash, this product should be interrupted and symptomatic treatment should be provided. For grade 4 rash, confirmed SJS or TEN, this product should be discontinued permanently.

Other Immune-related Adverse Reactions

Thrombocytopaenia

Immune-related thrombocytopaenia was reported in patients who received toripalimab (1.0%, 6/598), including death. Patients should be closely monitored for platelet level and whether there are symptoms and signs of bleeding tendency, such as gum bleeding, ecchymosis, and haematuria. In addition, other causes and concomitant medications should be excluded. In case of Grade 3 platelets decreased, administration will be discontinued temporarily and symptomatic and supportive treatment will be given until relieved to Grades 0-1. Whether to give corticosteroid treatment and whether the study treatment can be restarted will be determined according to clinical conditions. In case of Grade 4 thrombocytopaenia, administration will be discontinued permanently and active symptomatic treatment will be given. If necessary, corticosteroid treatment will be given.

Pancreatitis

Immune-related pancreatitis/amylase increased/lipase increased was reported in patients who received toripalimab (2.7%, 16/598). In case of grade 3 - 4 amylase increased or lipase increased or grade 2 - 3 pancreatitis, this product should be interrupted. In case of grade 4 or any recurrent pancreatitis, this product should be discontinued permanently.

Other

For suspected immune-related adverse reactions not listed above, full evaluation should be performed to exclude other causes. According to the severity of adverse reactions, for grade 2 - 3 adverse reactions occurring for the first time, this product should be interrupted and corticosteroid should be given. If the conditions are improved, this product can be resumed after reducing the dose of corticosteroid. In case of other grade 4 adverse reactions or recurrent grade 3 adverse reactions, grade 3 - 4 encephalitis, this product should be discontinued permanently.

If uveitis and other immune-related adverse reactions occur at the same time, whether Vogt-Koyanagi-Harada Syndrome occurs should be tested and systemic corticosteroid should be used to avoid permanent blindness.

Immune-related myocarditis was reported in other patients who used PD-1 inhibitors, such as cardiac enzymes increased accompanied by ECG change or chest pain, palpitations and lassitude. In case of grade 2 reactions, it is suggested to interrupt administration. In case of grade 3 – 4 reactions, administration should be discontinued permanently. The safety of resuming this product is still unknown.

Solid organ transplant rejection was reported in other patients who received PD-1 inhibitors. Therefore, benefit and possible organ rejection risk of this product should be weighed in these patients.

9.5 Pregnancy

Pregnancy itself is not considered as an AE. However, pregnancy of a patient or his/her partner from starting the study drugs to 90 days after the last dose should be followed up until the end/termination of pregnancy (abortion spontaneous, abortion induced, normal delivery, or congenital malformation), and records should be made.

All congenital malformations/birth defects are SAEs. Spontaneous abortion, ectopic pregnancy or complication of pregnancy meeting the criteria of SAE should also be regarded as SAEs and reported according to the reporting procedure of SAEs. Abortion induced without any complications should not be handled as an AE. Hospitalization for normal delivery of healthy infant should not be regarded as SAE. Precautions of Recording of Adverse Events

Diagnosis, symptoms and signs

If the diagnosis available, the diagnosis result should be recorded in the eCRF rather than single symptom and sign (e.g.: liver failure, rather than jaundice, elevation of transaminase and asterixis). However, if the symptoms and signs cannot be categorized as a single diagnosis during reporting period, each single event should be recorded as AE or SAE in the eCRF. If the diagnosis is confirmed later, its report should be updated as follow-up information.

Adverse events secondary to other events

Generally, the primary events should be recorded for secondary AEs (such as AE induced by other events or clinical sequelae), unless the secondary events are more severe or become SAEs. However, the secondary events with obviously clinical significance should be recorded as independent AEs in the eCRF if they occur at the time different from that of the primary events. If the correlation between the events remains unclear, they should be recorded in the eCRF separately.

Persistent or recurrent AEs

A persistent AE refers to the AE that always exists for it is not relieved between two evaluation time points of the patient. This type of AE should be recorded in the eCRF for only one time. The initial severity of events should be recorded, and should be updated when the events are worsened to record the highest grade of severity.

A recurrent AE refers to that has been relieved between two evaluation time points but recurs later. Occurrence of such events should be recorded respectively in the eCRF.

Abnormal Laboratory Findings or Vital Signs

Not all abnormal laboratory findings or vital signs need to be reported as AEs. Abnormal laboratory findings or vital signs meeting the following criteria can be reported as AEs:

- With clinical symptoms
- Lead to study medication change (e.g. dose adjustment, suspension or permanent discontinuation, etc.);
- Require medical intervention or change of concomitant therapy
- With obvious clinical significance at the discretion of the Investigator

Investigators are responsible for reviewing all the abnormal laboratory findings and vital signs, and determine whether to record them as AEs after medical judgment.

If the abnormal laboratory findings or vital signs with clinical significance are the representations (such as elevation of alkaline phosphatase and total bilirubin > 5 ULN resulted from cholecystitis) of a certain disease or syndrome, only the diagnosis (cholecystitis) should be recorded in the AE Record of the eCRF. Otherwise, the abnormal laboratory findings or vital signs should be recorded in the AE Record of the eCRF and whether the tested value is higher or lower than normal range should be indicated (e.g. recorded as "blood potassium increased" rather than "blood potassium abnormal"). If standard clinical terminology corresponding to the abnormal laboratory findings or vital signs exists, the terminology (such as "anemia" instead of "decreased hemoglobin") should be recorded in eCRF. The same abnormal laboratory findings or vital signs of clinical significance found in multiple follow-ups should not be recorded as an AE or a SAE repeatedly in the eCRF unless there is change in severity or etiology.

Death

Any death event in the whole trial period (from signing ICF to EOS), regardless of whether to be related to study drugs, should be recorded in the death report form of eCRF.

During record of death event, if AE leading to death exists, single medical concept should be applied to record the AE leading to death in the AE Record of the eCRF and the event should be regarded as a SAE for expedited reporting. If the cause of death remains unknown, "cause of death unknown" should be recorded in the AE Record of the eCRF and regarded as a SAE for expedited reporting. After that, the exact cause of death should be investigated further.

If it is confirmed that death is caused by tumor progression, it will not be recorded and reported as an AE/SAE. However, the investigator should record death information in the death report form of eCRF.

Pre-existing medical conditions

The pre-existing symptoms/signs of patient during the trial screening shall be recorded as AEs only when the severity, frequency and nature has worsened (except for deterioration of the target disease) after enrollment. Change from the previous condition should be reflected in the record, such as "increased frequency of headache".

Hospitalization, prolonged hospitalization or surgery

Any AE that results in hospitalization or prolonged hospitalization shall be recorded and

reported as a SAE except for the following situations:

- Scheduled hospitalization or prolonged hospitalization as requirement of the protocol (e.g. for administration, efficacy assessment, etc.)
- Hospitalization due to medical conditions that have existed prior to the participation in the study and remain unchanged. For example, selective surgery/treatment has been arranged before participating in the study.

However, if an existing disease worsens during the study period (such as surgery/treatment earlier than planned), the elective surgery/treatment for disease aggravation will be regarded as an AE.

The investigator should fill in all necessary information, including AE terms (diagnosis terms. If there is no diagnosis, record symptoms and signs, including abnormal laboratory findings), start date, end date, severity, whether it is an AESI, actions taken with study drugs, treatment for AE, outcome, seriousness and relationship with study drugs. If symptoms and signs cannot be grouped into a single diagnosis when reporting, each AE should be recorded separately.

Progressive disease

Any event that has been confirmed to be caused by PD will not be reported as an AE, including hospitalization or death caused by PD, and expedited reporting is not required.

Lack of therapeutic efficacy

If the disease treated with study drugs aggravates, it may be unable to determine whether it is lack of therapeutic efficacy or an AE. In this context, these changes should be regarded as lack of therapeutic efficacy other than AE unless the investigator considers condition aggravation is related to the used study drugs.

Overdose

If there is any concurrent AE, the AE should be recorded. If there is no concurrent AE, overdose should be recorded in the eCRF.

10 Statistical Analysis

10.1 Determination of Sample Size

Mainly the descriptive statistics due to the small number of subjects, and the results should be combined with professional analysis.

Statistics of the number of subjects enrolled, conditions of dropped out and removed subjects, demographics and other baseline characteristics and safety and efficacy analysis.

Statistical analysis procedures shall be subject to statistical analysis plan.

10.2 Populations for Statistical Analysis

Intention-to-treat Set (ITT): all randomized patients

Safety Set (SS): Among all enrolled patients, all the cases that have used the drug at least once are included in the safety set for safety analysis.

Full Analysis Set (FAS): It is an ideal case set based on the principle of intention-to-treat as much as possible, obtained by excluding the fewest and unreasonable cases from all subjects.

Per-protocol Set (PPS): Patients who complete the drug treatment according to the protocol without major deviation from the protocol, and complete all the evaluations constitute the PPS of this study. PPS includes at least the following criteria:

Meet the inclusion/exclusion criteria specified in the trial protocol;

Complete all scheduled medications and visits;

No drugs or treatments that may affect the efficacy evaluation are used during the trial.

10.3 General Principles for Statistical Analysis

For continuous variables, descriptive statistics will include count, mean, standard deviation, median, maximum and minimum. For categorical variables, descriptive statistics will include frequency and absolute rate or relative rate. Statistical analyses will be performed using SAS® V9.3 or above.

10.4 Statistical Analysis Method

10.4.1 Handling of Drop-out or Missing Data

Analysis of the primary and secondary endpoints will include the data of drop-out patients. Handling method of missing data will be described in Statistical Analysis Plan.

10.4.2 Multi-center Study

Independent efficacy analysis will not be performed by site.

10.4.3 Statistical Analysis

Patient disposition

Patients who complete or drop out the trial and the leading cause for drop-out (loss to follow-up, AEs, and poor compliance) will be analyzed, and the number and percentage of these patients will be calculated.

The number and percentage of patients in each analysis population will be calculated.

The main protocol violations in the trial will be described.

10.4.4 Demographics and Other Baseline Characteristics

Demographics including age, body height, sex and body weight, medical history (including melanoma history and surgery history), disease evaluation (including: TNM staging, position, existence, positioning and type of metastatic carcinoma) and other baseline characteristics will be descriptively and statistically analyzed.

10.4.5 Analysis of Compliance and Drug Exposure

The dose required by patients and the actual dose of drug will be recorded in the eCRF. Compliance of patients will be calculated according to the ratio of actual injection dose (number of doses) to the required injection dose (number of doses). Compliance of patients will be classified by < 80%, 80 - 120% and > 120%, and the number and percentage of patients will be listed by group.

10.4.6 Efficacy Analysis

Efficacy analysis will be based on FAS. Supportive analysis of the primary efficacy measure will be performed based on PP.

Primary efficacy analysis

Exact method will be adopted to estimate pathological response rates and 95% confidence interval.

Exact method will be adopted to estimate the incidence of clinical response rate and 95% confidence interval;

Secondary efficacy analysis

Kaplan Meier method will be adopted to estimate OS rate and 95% confidence interval after 2 years of administration; taking 2 years as time point, the median OS and 95% confidence interval will be estimated.

Kaplan Meier method will be adopted to estimate recurrence-free survival (RFS) rate after 1 year of administration and 95% confidence interval; taking 1 year as time point, the median RFS and 95% confidence interval will be estimated.

Kaplan Meier method will be adopted to estimate RFS rate after 2 year of administration and 95% confidence interval; taking 2 year as time point, the median RFS and 95% confidence interval will be estimated.

10.4.7 Safety Analyses

The safety analyses will be based upon the safety analysis set (SAS).

AEs

All adverse events (AEs) will be classified according to the Medical Dictionary for Regulatory Activities coding system and graded according to CTCAE 5.0. The number of cases and percentage of all TEAEs, grade 3 and above TEAEs, SAEs and study drug-related TEAEs,

study drug-related SAEs, TEAEs leading to discontinuation of study drug treatment, TEAEs leading to termination of trial, and surgery related AEs will be summarized by SOC, PT and group. Moreover, the severity of TEAEs and their correlation with study drugs will also be summarized by SOC, PT and group.

Laboratory tests

All laboratory test results and changes from baseline will be descriptively and statistically analyzed by planned time point and treatment group, and laboratory test abnormalities will be tabulated.

ECG test

Descriptive statistics of ECG test results and changes from baseline will be performed.

Vital signs, physical examination and other safety-related examinations

Descriptive statistics of vital sign test results and changes from baseline will be performed.

List physical examination results.

ECOG PS will be summarized by the planned time point.

Concomitant medications

Concomitant medication refers to a non-investigational product that meets one of the following conditions:

- All drugs start from the first dose of therapeutic drug or after the first dose of therapeutic drug;
- All drugs start before the first dose of therapeutic drug and are continued after the first dose of therapeutic drug;

List concomitant medications.

10.4.8 Interim Analysis

No interim analysis is planned for this study.

10.4.9 Subgroup Analysis

Subgroup analysis will be explored according to data. Subgroup analysis may include:

- Tumor stage: stage IIIb/IIIc, stage IV M1a
- Gender: male vs female
- ECOG PS score: 0 vs 1

11 Data Management

11.1 Case Report Form

In the study, eCRF will be adopted to collect data. Data statistical company will prepare a data management plan. The funder or its representative will provide the structure and format of eCRF which will be made according to the provided instructions.

Passwords will be set for eCRF to protect access right and only study personnel directly taking part in the study have an access to the eCRF. Data will be completely entered to the eCRF by test personnel or study coordinator. eCRF should be completed after patient evaluation or discussion as soon as possible. If the data need to be modified due to entry mistake or other reasons, electronic audit trail will track these modifications. Study CRA and other regulatory auditors should be given an access to eCRF.

eCRF should be reviewed by the investigator, signed in an electronic manner and dated.

The investigator should ensure the data are entered in a timely, accurate, integrated and legible manner.

If data need to be modified after the investigator reviews and confirms the data, the data should be confirmed by the investigator again.

11.2 Data Entry and Modification

Data management and biostatistics department of WuXi Clinical will process data obtained from the clinical study according to relevant SOPs.

The study will adopt electronic data capture (EDC) system, and the study data will be input into the eCRF by the investigator or authorized staff of the study site. Before initiating study site or data input, investigators and authorized staff of study site will receive appropriate training and proper safety measures will be taken.

All input data are in Chinese. The eCRF should be completed during or after visits as soon as possible and updated timely to ensure it can show the latest status of patients participating in the study. To avoid differences in outcome evaluation between different evaluators, please ensure that baseline and all subsequent efficacy and safety evaluation of the same patient are performed by the same evaluator. The investigator should audit the data to ensure the accuracy and correctness of all data input to the eCRF. If some evaluations are not conducted during the study, or some information are not available, applicable or unknown, the investigator should record it in the eCRF. The investigator should electronically sign the audited data.

Clinical research associate (CRA) will review the eCRF and evaluate its integrity and consistency. CRA will compare the eCRF with source documents to ensure the consistency of critical data. The investigator or personnel designated by the investigator will be responsible for inputting, correcting and modifying the data, and CRA does not have the right to input data. Data in the eCRF will be submitted to the data server. Any modifications to the data will be recorded in the audit trail that have recorded the modification reasons, name of operator, modification time and date. The role and right of staff of study site responsible for data input will be predetermined. If there are some queries about the data, CRA or data manager will

propose queries in the EDC and staff of the study site will be responsible for answering. EDC system will record the audit trail of queries, including name of investigator, time and date.

Unless otherwise specified, eCRF will only be used as a form for collecting data and cannot be used as original data. Source documents are all records used by the investigator or hospital, which are related to patients and able to prove the existence of patients, inclusion and exclusion criteria, and their participation in the study, including laboratory records, ECG results, memorandum, pharmacy drug dispensing records and patient file folder.

The investigator will be responsible for maintaining all source documents and providing them to CRA for monitoring at each visit. In addition, regardless of the duration of patients participating in the study, the investigator should submit a complete eCRF for each patient participating in the study. It is necessary to seriously audit study number and patient number on all supporting documents (e.g., laboratory record or hospital record) submitted together with the eCRF, and delete all personal private information (including name of patient), or make it illegible, to protect the privacy of patients.

The investigators can automatically add to eCRF with their user ID. Records of electronic signature will be used to prove that the investigator has audited the record and ensure the accuracy of the data on the record. Electronic signature will be completed with the user ID and password of the investigator, and the system will automatically provide the date and time of the signature simultaneously, and the investigator cannot share the user ID and password with other personnel. If data in the eCRF should be modified, it should be conducted according to the working procedures defined in the EDC system. All modifications and modification reasons should be recorded in the audit trail.

Prior and concomitant medications will be coded with the latest version of WHO-Drug Dictionary Enhanced (WHO-DDE). Medical history and AEs will be coded according to the Medical Dictionary for Regulatory Activities (MedDRA).

11.3 Locking of Database

The database will be locked after all queries have been solved and the integrity and accuracy of the data in the database are confirmed. If problems are found after locking the database, the data can be modified after obtaining written approval of the investigator.

12 Quality Assurance

12.1 Monitoring

The funder has moral, legal and scientific obligations to ensure the study is conducted according to the established study rules, GCP guidelines, applicable management requirements and regulations. As a coordinated measure to fulfill these obligations, the funder's CRA or its representative will visit the study site regularly in addition to frequent communication with the study site via telephone and in written form during the study period. On-site monitoring, inquiry via telephone and regular eCRF verification will be performed to evaluate patient enrollment, protocol compliance, integrity and accuracy of the data entered to eCRFs, validation of data in eCRFs and original documents, occurrence of AEs. Investigator must provide the CRA with access to all original data and study documents.

Study site must complete CRFs timely and allow study CRA to regularly review them.

12.2 Audit and Inspection

The funder or its representative may audit the study site. Audit includes but is not limited to supply of study drugs, whether the required documents are complete, informed consent process, and comparison between eCRF and original documents. The investigator will agree and take part in audit in a reasonable manner and time.

The purpose of audit is to confirm whether the study complies with ethic, regulatory and quality requirements.

13 Study Management

13.1 Imaging Assessment

The study will set an imaging assessment group to evaluate the efficacy of patients on CT or MRI as per RECIST 1.1. Meanwhile, for patients who receive treatment, efficacy should be evaluated and recorded based on iRECIST and iT-RECIST criteria. The criteria for imaging evaluation are shown in relevant sections.

13.2 Management Structure

The study will be uniformly managed by investigators who will be responsible for clinical monitoring, project management, biostatistics, data management, medical writing and drug safety report.

13.3 Preservation of Original Data

The original data refer to the original records of data of relevant patients that are obtained from the clinical study. These original records are called original documents, including but not limited to medical records (medical history, nursing record, and prescription record, etc.), electronic data, screening log, report of laboratory test results, and test results of medical devices (ECG, CT or MRI images, etc.). All original documents of the study will be preserved by study site and investigators. The original informed consent form will be preserved with the method developed by clinical trial medical institution.

The investigator will provide enough and accurate original documents for each randomized patient to record all test results and other relevant data, and will preserve these documents in an appropriate manner.

13.4 Recording of Study Data

All data obtained from the study will be owned by the sponsor.

Records should be preserved according to current GCP guidelines. All necessary study documents, including records, original documents, eCRFs of patients, and stock list of study drugs, should be put on record.

If the investigator leaves, withdraws from the study or retires, the responsibility of maintaining records will be transferred to another person. A notice of transfer should be developed.

13.5 Confidentiality

All information related to health status of patients obtained during study operations will be considered confidential. Disclosure of any of the information should obtain written approval.

The investigator should ensure that the privacy of each patient is protected. In the CRFs and other documents submitted to the funder, patients cannot be identified with name but with their initials and unique patient code.

Under special circumstances, certain personnels may see genetic data and personal identifier of a patient. For example, in the event of medical emergency, thefunder, its representative physician, or investigator will be aware of the patient's identifier and have access to the patient's genetic data. In addition, relevant regulatory authorities can request access to relevant

documents.

13.6 Review of the Ethics Committee (EC)

The sponsor or the agent designated by the sponsor will be responsible for ensuring that the approval of relevant regulatory authorities is obtained according to the requirements of the country.

Patients cannot take part in the study before obtaining approval.

13.7 Protocol Amendment

According to ICH-GCP E6 guideline, the investigator should not deviate from or change the study protocol without the approval of the sponsor and EC's approval for protocol amendment, unless it is necessary to eliminate direct harm to study patients, or the change only involves logistics or administration of the study (e.g., change of displayer, change of telephone number).

Any changes to the protocol shall be treated as protocol amendment. Any possible modification should be approved by the sponsor. Written protocol amendment should be submitted to relevant regulatory authorities and responsible EC. The investigator can implement these changes only after obtaining IEC's approval for protocol amendment, unless it is necessary to eliminate obvious direct harm to patients. In this context, EC should be notified within 5 days upon protocol modification.

All protocol modifications should obtain written approval of relevant regulatory authorities and EC. In terms of administrative modifications, only notification is required, and written approval is not required. Once approved, protocol amendment will be distributed to all personnel who have received the original protocol, and operation instructions will be attached to protocol amendment.

If local EC, investigator and/or the sponsor considers protocol amendment changes study design, process and/or increases potential risk to patients, the currently approved written informed consent form needs to be modified. The revised informed consent form should be reviewed and approved by the sponsor, relevant regulatory authorities and EC. In this context, informed consent of patients should be obtained again before they continue taking part in the study.

13.8 Publication of Study Results

The investigator can publish study results after completing the study.

13.9 Protocol Compliance and Protocol Violation

The study protocol should be thoroughly read and instructions should be followed. There will be exceptions in emergencies, that is, direct intervention is required for the rights, safety and health of patients based on the judgment of the investigator or assistant investigator that is designated by the investigator and trained appropriately with professional qualification.

For important protocol deviation due to emergencies, accident or mistake, the investigator or the person designated by the investigator should contact the medical monitor via telephone as soon as possible. Therefore, a consensus on whether patients can continue the study can be reached as soon as possible. The investigator and medical monitor will record the decision.

13.10 Clinical Study Report

13.11 The final clinical study report will be written according to the NMPA guidelines for structure and contents of clinical study report. The final clinical study report must be prepared no matter whether the study is completed or prematurely terminated. Insurance, Compensation and Indemnification

The funder is responsible for providing an appropriate clinical study insurance contract.

Deviation from study protocol, especially not complying with the dose instruction planned in the study protocol, other medication methods, other indications, and prolongation of treatment duration will not be allowed and not be included in the legal insurance plan of patients.

13.12 Termination of the Study

The funder can terminate the study. For patients' maximum benefit and rational medical or ethical judgment, if the investigator and the funder agree, the study can be terminated prematurely at any time. During terminating the study, the funder and the investigator will ensure sufficient considerations for protection of patients' interests.

13.13 Management of Study Site Documents

The investigator has the responsibility to ensure the maintenance of study site documents. Study site documents will include but are not limited to the following information:

1. Investigator's Brochure;
2. Latest signed study protocol and any previous version of study protocol;
3. Study Protocol Amendment (if applicable);
4. Operation manual (if applicable);
5. Latest informed consent form (blank) and any previous version of informed consent form;
6. Curriculum vitae of the investigator and assistant investigator and photocopy of their license required by the laws; name of assistant investigator should be indicated in the table. The investigator should complete all regulatory documents required by GCP and local or national regulatory requirements;
7. EC's approval document for study protocol, informed consent form, any protocol amendment and all revised versions of informed consent form;
8. Correspondence related to study operations among the investigator, EC and the funder;
9. Certificate of laboratory;
10. Monitoring log;
11. List of study drugs;
12. List of signatures of staff filling in the eCRF; and
13. List of signatures of staff filling in drug counting summary.

14 References

[1] CFDA's Good Clinical Practice, Amendment on December 01, 2016.

[2] Ferlay J, Soerjomataram I, Dikshit R, et al. Cancer incidence and mortality worldwide: Sources, methods and major patterns in GLOBOCAN 2012. Int J Cancer, 2015, 136 (5): E359-86.

[3] Guidelines for the Diagnosis and Treatment of Melanoma in China, 2015 edition

[4] Garbe C, Eigentler T K, Keilholz U, et al. Systematic review of medical treatment in melanoma: current status and future prospects. Oncologist, 2011, 16 (1): 5-24.

[5] Ribas A, Dummer R, Puzanov I,et al. Oncolytic Virotherapy Promotes Intratumoral T Cell Infiltration and Improves Anti-PD-1 Immunotherapy.Cell. 2017 Sep 7;170 (6): 1109- 1119.

[6] Middleton M R, Grob J J, Aaronson N, et al. Middleton, MR, Grob, JJ, Aaronson, N, Fierlbeck, G, Tilgen, W, Seiter, S et al. Randomized phase III study of temozolomide versus dacarbazine in the treatment of patients with advanced metastatic malignant melanoma. J Clin Oncol, 2000, 18 (1): 158-66.

[7] Avril M F, Aamdal S, Grob J J, et al. Avril, MF, Aamdal, S, Grob, JJ, Hauschild, A, Mohr, P, Bonerandi, JJ et al. Fotemustine compared with dacarbazine in patients with disseminated malignant melanoma: a phase III study. J Clin Oncol, 2004, 22 (6): 1118-25.

[8] Bedikian A Y, Millward M, Pehamberger H, et al. Bcl-2 antisense (oblimersen sodium) plus dacarbazine in patients with advanced melanoma: the Oblimersen Melanoma Study Group. J Clin Oncol, 2006, 24 (29): 4738-45.

[9] Legha S S, Ring S, Eton O, et al. Development of a biochemotherapy regimen with concurrent administration of cisplatin, vinblastine, dacarbazine, interferon alfa, and interleukin-2 for patients with metastatic melanoma. J Clin Oncol, 1998, 16 (5): 1752-9.

[10] Chapman P B, Einhorn L H, Meyers M L, et al. Phase III multicenter randomized trial of the Dartmouth regimen versus dacarbazine in patients with metastatic melanoma. J Clin Oncol, 1999, 17 (9): 2745-51.

[11] Andtbacka RH, Kaufman HL, Collichio F, et al. Talimogene Laherparepvec Improves Durable Response Rate in Patients With Advanced Melanoma. J Clin Oncol 2015;33:2780-2788.

[12] Investigator’s Brochure for OrienX010, version 5.3 (October 30, 2015)

[13] Bohnsack O, Hoos A, Ludajic K. 1070P-Adaptation of the immune related response criteria: irRECIST. Annals of Oncology, 2014, 25 (suppl 4) .

[14] Seymour L, Bogaerts J, Perrone A, et al. iRECIST: guidelines for response criteria for use in trials testing immunotherapeutics. Lancet Oncol, 2017, 18 (3): e143-e152.

[15] Alexander M.M, Eggermont and Kirkwood: Re-evaluating the role of dacarbazine inmetastatic melanoma: what we have learned in 30 years. Eur J Cancer, 2004, 40 (12); 1825-36.

[16] Gregory V. Goldmacher,Anuradha D. Khilnani, et al. Response Criteria for Intratumoral Immunotherapy in Solid Tumors: itRECIST. JCO (in review).

Appendixes I ECOG Performance Status Scoring Criteria

Score	Activity Level
0	Fully active, able to carry on all pre-disease performance without restriction
1	Restricted in physically strenuous activity but ambulatory and able to carry out work of a light or sedentary nature (e.g., light housework, office work).
2	Ambulatory and capable of all self-care but unable to carry out any work activities; up and about more than 50% of waking hours.
3	Capable of only limited self-care, confined to bed or chair more than 50% of waking hours
4	Totally confined to bed, cannot carry on any self-care.
5	Death

Appendixes II Response Evaluation Criteria in Solid Tumors (RECIST) 1.1

1. Measurability of tumour at baseline

1.1. Definitions

At baseline, tumour lesions/lymph nodes will be categorised measurable or non-measurable as follows:

1.1.1. Measurable:

Tumour lesions: Must be accurately measured in at least one dimension (longest diameter in the plane of measurement is to be recorded) with a minimum size of:

- 10 mm by CT scan (CT scan slice thickness no greater than 5 mm)
- 10 mm caliper measurement by clinical exam (lesions which cannot be accurately measured with calipers should be recorded as non-measurable)
- 20 mm by chest X-ray

Malignant lymph nodes: To be considered pathologically enlarged and measurable, a lymph node must be ≥15mm in short axis when assessed by CT scan (CT scan slice thickness recommended to be no greater than 5 mm). At baseline and in follow-up, only the short axis will be measured and followed.

1.1.2. Non-measurable

All other lesions, including small lesions (longest diameter <10mm or pathological lymph nodes with ≥10 to <15mm short axis) as well as truly non-measurable lesions. Lesions considered truly non-measurable include: leptomeningeal disease, ascites, pleural or pericardial effusion, inflammatory breast disease, lymphangitic involvement of skin or lung, abdominal masses/abdominal organomegaly identified by physical exam that is not measurable by reproducible imaging techniques.

1.1.3. Special considerations regarding lesion measurability Bone lesions, cystic lesions, and lesions previously treated with local therapy require particular comment

Bone lesions:

- Bone scan, PET scan or plain films are not considered adequate imaging techniques to measure bone lesions. However, these techniques can be used to confirm the presence or disappearance of bone lesions;
- Lytic bone lesions or mixed lytic-blastic lesions, with identifiable soft tissue components, that can be evaluated by crosssectional imaging techniques such as CT or MRI can be considered as measurable lesions if the soft tissue component meets the definition of measurability described above;
- Blastic bone lesions are non-measurable.

Cystic lesions:

- Lesions that meet the criteria for radiographically defined simple cysts should not be considered as malignant lesions (neither measurable nor non-measurable) since they are, by definition, simple cysts;
- "Cystic lesions" thought to represent cystic metastases can be considered as measurable lesions, if they meet the definition of measurability described above. However, if noncystic lesions are present in the same patient, these are preferred for selection as target lesions.

Lesions with prior local treatment:

- Tumor lesions situated in a previously irradiated area, or in an area subjected to other loco-regional therapy, are usually not considered measurable unless there has been demonstrated progression in the lesion. Study protocols should detail the conditions under which such lesions would be considered measurable.

1.2. Specifications by Methods of Measurements

1.2.1. Measurement of Lesions

All measurements should be recorded in metric notation, using calipers if clinically assessed. All baseline evaluations should be performed as close as possible to the treatment start and never more than 4 weeks before the beginning of the treatment.

1.2.2. Assessment Methods

The same method of assessment and the same technique should be used to characterise each identified and reported lesion at baseline and during follow-up. Imaging based evaluation should always be done rather than clinical examination unless the lesion(s) being followed cannot be imaged but are assessable by clinical exam.

Clinical lesions: Clinical lesions will only be considered measurable when they are superficial and ≥ 10 mm diameter as assessed using calipers (e.g. skin nodules). For the case of skin lesions, documentation by colour photography including a ruler to estimate the size of the lesion is suggested. As noted above, when lesions can be evaluated by both clinical exam and imaging, imaging evaluation should be undertaken since it is more objective and may also be reviewed at the end of the study.

Chest X-ray: Chest CT is preferred over chest X-ray, particularly when progression is an important endpoint, since CT is more sensitive than X-ray, particularly in identifying new lesions. However, lesions on chest X-ray may be considered measurable if they are clearly defined and surrounded by aerated lung.

CT, MRI: CT is the best currently available and reproducible method to measure lesions selected for response assessment. This guideline has defined measurability of lesions on CT scan based on the assumption that CT slice thickness is 5 mm or less. When CT scans have slice thickness greater than 5 mm, the minimum size for a measurable lesion should be twice the slice thickness. MRI is also acceptable in certain situations (e.g. for body scans).

Ultrasound: Ultrasound is not useful in assessment of lesion size and should not be used as a method of measurement. Ultrasound examinations cannot be reproduced in their entirety for independent review at a later date and, because they are operator dependent, it cannot be

guaranteed that the same technique and measurements will be taken from one assessment to the next. If new lesions are identified by ultrasound in the course of the study, confirmation by CT or MRI is advised. If there is concern about radiation exposure at CT, MRI may be used instead of CT in selected instances.

Endoscopy, laparoscopy: The utilization of these techniques for objective tumor evaluation is not advised. However, they can be useful to confirm complete pathological response when biopsies are obtained or to determine relapse in trials where recurrence following complete response or surgical resection is an endpoint.

Tumor markers: Tumor markers alone cannot be used to assess objective tumor response. If markers are initially above the upper normal limit, however, they must normalise for a patient to be considered in complete response. Because tumor markers are disease specific, instructions for their measurement should be incorporated into protocols on a disease specific basis. Specific guidelines for both CA-125 response (in recurrent ovarian cancer) and PSA response (in recurrent prostate cancer), have been published. In addition, the Gynecologic Cancer Intergroup has developed CA125 progression criteria which are to be integrated with objective tumor assessment for use in first-line trials in ovarian cancer.

Cytology, histology: These techniques can be used to differentiate between PR and CR in rare cases if required by protocol (for example, residual lesions in tumor types such as germ cell tumors, where known residual benign tumors can remain). When effusions are known to be a potential adverse effect of treatment (e.g. with certain taxane compounds or angiogenesis inhibitors), the cytological confirmation of the neoplastic origin of any effusion that appears or worsens during treatment can be considered if the measurable tumor has met criteria for response or stable disease in order to differentiate between response (or stable disease) and progressive disease.

2. Tumor Response Evaluation

2.1. Assessment of Overall Tumor Burden and Measurable Disease

To assess objective response or future progression, it is necessary to estimate the overall tumor burden at baseline and use this as a comparator for subsequent measurements. Only patients with measurable disease at baseline should be included in protocols where objective tumor response is the primary endpoint. Measurable disease is defined by the presence of at least one measurable lesion. In studies where the primary endpoint is tumor progression (either time to progression or proportion with progression at a fixed date), the protocol must specify if entry is restricted to those with measurable disease or whether patients having non-measurable disease only are also eligible.

2.2. Baseline Documentation of "Target" and "Non-target" Lesions

When more than one measurable lesion is present at baseline all lesions up to a maximum of five lesions total (and a maximum of two lesions per organ) representative of all involved organs should be identified as target lesions and will be recorded and measured at baseline (this means in instances where patients have only one or two organ sites involved a maximum of two and four lesions respectively will be recorded).

Target lesions should be selected on the basis of their size (lesions with the longest diameter), be representative of all involved organs, but in addition should be those that lend themselves to reproducible repeated measurements. It may be the case that, on occasion, the largest lesion does not lend itself to reproducible measurement in which circumstance the next largest lesion which can be measured reproducibly should be selected.

Lymph nodes merit special mention since they are normal anatomical structures which may be visible by imaging even if not involved by tumor. Pathological nodes which are defined as measurable and may be identified as target lesions must meet the criterion of a short axis of ≥15mm by CT scan. Only the short axis of these nodes will contribute to the baseline sum. The short axis of the node is the diameter normally used by radiologists to judge if a node is involved by solid tumor. Nodal size is normally reported as two dimensions in the plane in which the image is obtained (for CT scan this is almost always the axial plane; for MRI the plane of acquisition may be axial, saggital or coronal). The smaller of these measures is the short axis. For example, an abdominal node which is reported as being 20 mm × 30 mm has a short axis of 20 mm and qualifies as a malignant, measurable node. In this example, 20 mm should be recorded as the node measurement. All other pathological nodes (those with short axis ≥10 mm but <15 mm) should be considered non-target lesions. Nodes that have a short axis <10 mm are considered non-pathological and should not be recorded or followed.

A sum of the diameters (longest for non-nodal lesions, short axis for nodal lesions) for all target lesions will be calculated and reported as the baseline sum diameters. If lymph nodes are to be included in the sum, then as noted above, only the short axis is added into the sum. The baseline sum diameters will be used as reference to further characterise any objective tumor regression in the measurable dimension of the disease.

All other lesions (or sites of disease) including pathological lymph nodes should be identified as non-target lesions and should also be recorded at baseline. Measurements are not required and these lesions should be followed as “present”, “absent”, or in rare cases “unequivocal progression”. In addition, it is possible to record multiple non-target lesions involving the same organ as a single item on the case report form (e.g. “multiple enlarged pelvic lymph nodes” or “multiple liver metastases”).

2.3. Response Criteria

2.3.1. Evaluation of Target Lesions

Complete Response (CR): Disappearance of all target lesions. Any pathological lymph nodes (whether target and non-target) must have reduction in short axis to <10 mm.

Partial Response (PR): At least a 30% decrease in the sum of diameters of target lesions, taking as reference the baseline sum diameters.

Progressive Disease (PD): At least a 20% increase in the sum of diameters of target lesions, taking as reference the smallest sum on study (this includes the baseline sum if that is the smallest on study). In addition to the relative increase of 20%, the sum must also demonstrate an absolute increase of at least 5 mm. (Notes: the appearance of one or more new lesions is also considered progression).

Stable Disease (SD): Neither sufficient shrinkage to qualify for PR nor sufficient increase to qualify for PD, taking as reference the smallest sum diameters while on study.

2.3.2. Special Notes on the Assessment of Target Lesions Lymph nodes

Lymph nodes identified as target lesions should always have the actual short axis measurement recorded (measured in the same anatomical plane as the baseline examination), even if the nodes regress to below 10 mm on study. This means that when lymph nodes are included as target lesions, the "sum" of lesions may not be zero even if complete response criteria are met, since a normal lymph node is defined as having a short axis of < 10 mm. Case report forms or other data collection methods may therefore be designed to have target nodal lesions recorded in a separate section where, in order to qualify for CR, each node must achieve a short axis < 10 mm. For PR, SD, and PD evaluation criteria, the actual short axis measurement of the nodes is to be included in the sum of target lesions.

Target lesions that become "too small to measure". While on study, all lesions (nodal and non-nodal) recorded at baseline should have their actual measurements recorded at each subsequent evaluation, even when very small (e.g. 2 mm). However, sometimes lesions or lymph nodes which are recorded as target lesions at baseline become so faint on CT scan that the radiologist may not feel comfortable assigning an exact measure and may report them as being "too small to measure". When this occurs, it is important that a value be recorded on the case report form. If it is the opinion of the radiologist that the lesion has likely disappeared, the measurement should be recorded as 0 mm. If the lesion is believed to be present and is faintly seen but too small to measure, a default value of 5 mm should be assigned (Note: It is less likely that this rule will be used for lymph nodes since they usually have a definable size when normal and are frequently surrounded by fat such as in the retroperitoneum; however, if a lymph node is believed to be present and is faintly seen but too small to measure, a default value of 5 mm should be assigned in this circumstance as well). This default value is derived from the 5 mm CT slice thickness (but should not be changed with varying CT slice thickness). The measurement of these lesions is potentially non-reproducible, therefore providing this default value will prevent false responses or progressions based upon measurement error. To reiterate, however, if the radiologist is able to provide an actual measure, that should be recorded, even if it is below 5 mm.

Lesions that split or coalesce on treatment. When non-nodal lesions "fragment", the longest diameters of the fragmented portions should be added together to calculate the target lesion sum. Similarly, as lesions coalesce, a plane between them may be maintained that would aid in obtaining maximal diameter measurements of each individual lesion. If the lesions have truly coalesced such that they are no longer separable, the vector of the longest diameter in this instance should be the maximal longest diameter for the "coalesced lesion".

2.3.3. Evaluation of Non-target Lesions

This section provides definitions of the criteria used to determine the tumor response for the group of non-target lesions. While some non-target lesions may actually be measurable, they need not be measured and instead should be assessed only qualitatively at the time points specified in the protocol.

Complete Response (CR): Disappearance of all non-target lesions and normalization of tumor marker level. All lymph nodes must be non-pathological in size (<10 mm short axis).

Non-CR/Non-PD: Persistence of one or more non-target lesion(s) and/or maintenance of tumor marker level above the normal limits.

Progressive disease (PD): Unequivocal progression of existing non-target lesions. (Notes: the appearance of one or more new lesions is also considered progression).

2.3.4. Special Notes on Assessment of Progression of Non-target Disease

The concept of progression of non-target disease requires additional explanation as follows: *When the patient also has measurable disease.* In this setting, to achieve "unequivocal progression" on the basis of the non-target disease, there must be an overall level of substantial worsening in non-target disease such that, even in presence of SD or PR in target disease, the overall tumor burden has increased sufficiently to merit discontinuation of therapy. A modest "increase" in the size of one or more non-target lesions is usually not sufficient to qualify for unequivocal progression status. The designation of overall progression solely on the basis of change in non-target disease in the face of SD or PR of target disease will therefore be extremely rare.

When the patient has only non-measurable disease. This circumstance arises in some phase 3 trials when it is not a criterion of study entry to have measurable disease. The same general concepts apply here as noted above, however, in this instance there is no measurable disease assessment to factor into the interpretation of an increase in non-measurable disease burden. Because worsening in non-target disease cannot be easily quantified (by definition: if all lesions are truly non-measurable) a useful test that can be applied when assessing patients for unequivocal progression is to consider if the increase in overall disease burden based on the change in non-measurable disease is comparable in magnitude to the increase that would be required to declare PD for measurable disease: i.e. an increase in tumor burden representing an additional 73% increase in 'volume' (which is equivalent to a 20% increase diameter in a measurable lesion). Examples include an increase in a pleural effusion from "trace" to "large", an increase in lymphangitic disease from localised to widespread, or may be described in protocols as "sufficient to require a change in therapy". If "unequivocal progression" is seen, the patient should be considered to have had overall PD at that point. While it would be ideal to have objective criteria to apply to non-measurable disease, the very nature of that disease makes it impossible to do so, therefore the increase must be substantial.

2.3.5. New Lesions

The appearance of new malignant lesions denotes disease progression; therefore, some comments on detection of new lesions are important. There are no specific criteria for the identification of new radiographic lesions; however, the finding of a new lesion should be unequivocal: i.e. not attributable to differences in scanning technique, change in imaging modality or findings thought to represent something other than tumour (for example, some "new" bone lesions may be simply healing or flare of pre-existing lesions). This is particularly important when the patient's baseline lesions show partial or complete response. For example, necrosis of a liver lesion may be reported on a CT scan report as a "new" cystic lesion, which

it is not.

A lesion identified on a follow-up study in an anatomical location that was not scanned at baseline is considered a new lesion and will indicate disease progression. An example of this is the patient who has visceral disease at baseline and while on study has a CT or MRI brain ordered which reveals metastases. The patient's brain metastases are considered to be evidence of PD even if he/she did not have brain imaging at baseline.

If a new lesion is equivocal, for example because of its small size, continued therapy and follow-up evaluation will clarify if it represents truly new disease. If repeat scans confirm there is definitely a new lesion, then progression should be declared using the date of the initial scan.

While FDG-PET response assessments need additional study, it is sometimes reasonable to incorporate the use of FDG-PET scanning to complement CT scanning in assessment of progression (particularly possible "new" disease). New lesions on the basis of FDG-PET imaging can be identified according to the following algorithm:

a. Negative FDG-PET at baseline, with a positive FDG-PET at follow-up is a sign of PD based on a new lesion.

b. No FDG-PET at baseline and a positive FDG-PET at follow-up:

   If the positive FDG-PET at follow-up corresponds to a new site of disease confirmed by CT, this is PD. If the positive FDG-PET at follow-up is not confirmed as a new site of disease on CT, additional follow-up CT scans are needed to determine if there is truly progression occurring at that site (if so, the date of PD will be the date of the initial abnormal FDG-PET scan). If the positive FDG-PET at follow-up corresponds to a pre-existing site of disease on CT that is not progressing on the basis of the anatomic images, this is not PD.

2.4. Evaluation of Best Overall Response

The best overall response is the best response recorded from the start of the study treatment until the end of treatment taking into account any requirement for confirmation. On occasion a response may not be documented until after the end of therapy so protocols should be clear if post-treatment assessments are to be considered in determination of best overall response. Protocols must specify how any new therapy introduced before progression will affect best response designation. The patient's best overall response assignment will depend on the findings of both target and non-target disease and will also take into consideration the appearance of new lesions. Furthermore, depending on the nature of the study and the protocol requirements, it may also require confirmatory measurement. Specifically, in non-randomised trials where response is the primary endpoint, confirmation of PR or CR is needed to deem either one the "best overall response".

2.4.1. Time Point Response

It is assumed that at each protocol specified time point, a response assessment occurs. Table 1 on the next page provides a summary of the overall response status calculation at each time point for patients who have measurable disease at baseline.

When patients have non-measurable (therefore non-target) disease only, Table 2 is to be used.

2.4.2. Missing Assessments and Inevaluable Designation

When no imaging/measurement is done at all at a particular time point, the patient is not evaluable (NE) at that time point. If only a subset of lesion measurements are made at an assessment, usually the case is also considered NE at that time point, unless a convincing argument can be made that the contribution of the individual missing lesion(s) would not change the assigned time point response. This would be most likely to happen in the case of PD. For example, if a patient had a baseline sum of 50 mm with three measured lesions and at follow-up only two lesions were assessed, but those gave a sum of 80 mm, the patient will have achieved PD status, regardless of the contribution of the missing lesion.

2.4.3. Best Overall Response: All Time Points

The best overall response is determined once all the data for the patient is known.

Best response determination in trials where confirmation of complete or partial response IS NOT required: Best response in these trials is defined as the best response across all time points (for example, a patient who has SD at first assessment, PR at second assessment, and PD on last assessment has a best overall response of PR). When SD is believed to be best response, it must also meet the protocol specified minimum time from baseline. If the minimum time is not met when SD is otherwise the best time point response, the patient's best response depends on the subsequent assessments. For example, a patient who has SD at first assessment, PD at second and does not meet minimum duration for SD, will have a best response of PD. The same patient lost to follow-up after the first SD assessment would be considered inevaluable.

Best response determination in trials where confirmation of complete or partial response IS required: Complete or partial responses may be claimed only if the criteria for each are met at a subsequent time point as specified in the protocol (generally 4 weeks later). In this circumstance, the best overall response can be interpreted as in Table 3.

2.4.4. Special notes on response assessment

When nodal disease is included in the sum of target lesions and the nodes

decrease to "normal" size (< 10 mm), they may still have a measurement reported on scans. This measurement should be recorded even though the nodes are normal in order not to overstate progression should it be based on increase in size of the nodes. As noted earlier, this means that patients with CR may not have a total sum of "zero" on the case report form (CRF).

In trials where confirmation of response is required, repeated 'NE' time point assessments may complicate best response determination. The analysis plan for the trial must address how missing data/assessments will be addressed in determination of response and progression For example, in most trials it is reasonable to consider a patient with time point responses of PR-NE-PR as a confirmed response.

Patients with a global deterioration of health status requiring discontinuation of treatment without objective evidence of disease progression at that time should be reported as "symptomatic deterioration". Every effort should be made to document objective progression

even after discontinuation of treatment. Symptomatic deterioration is not a descriptor of an objective response: it is a reason for stopping study therapy. The objective response status of such patients is to be determined by evaluation of target and non-target disease as shown in Tables 1–3.

Conditions that define "early progression, early death and inevaluability" are study specific and should be clearly described in each protocol (depending on treatment duration, treatment periodicity).

In some circumstances it may be difficult to distinguish residual disease from normal tissue. When the evaluation of complete response depends upon this determination, it is recommended that the residual lesion be investigated (fine needle aspirate/biopsy) before assigning a status of complete response.

FDG-PET may be used to upgrade a response to a CR in a manner similar to a biopsy in cases where a residual radiographic abnormality is thought to represent fibrosis or scarring. The use of FDG-PET in this circumstance should be prospectively described in the protocol and supported by disease specific medical literature for the indication. However, it must be acknowledged that both approaches may lead to false positive CR due to limitations of FDG-PET and biopsy resolution/sensitivity.

Table 1 – Time point response: patients with target (+/– non-target) disease

Target Lesions	Non-target Lesions	New Lesions	Overall Response
CR	CR	No	CR
CR	Non-CR/Non-PD	No	PR
CR	Not evaluated	No	PR
PR	Non-PD or	No	PR
SD	Not all evaluated		
	Non-PD or	No	SD
Not all evaluated	Not all evaluated		NE
PD	Non-PD	No	PD
Any	Any	Yes or No	PD
Any	PD	Yes or No	PD
	Any	No Yes	

CR = complete response, PR = partial response, SD = stable disease, PD = progressive disease, NE = not evaluable.

Table 2 – Time Point Response: Patients with Non-target Disease Only

Non-target Lesions	New Lesions	Overall Response
CR	No	CR
Non-CR/Non-PD	No	Non-CR/Non-PD[a]
Not all evaluated	No	NE
Unequivocal PD	Yes or No	PD
Any	Yes	PD

CR = complete response, PD = progressive disease, and NE = not evaluable.

a "Non-CR/non-PD" is preferred over "stable disease" for non-target disease since SD is increasingly used as endpoint for assessment of efficacy in some trials so to assign this category when no lesions can

be measured is not advised.

For equivocal findings of progression (e.g. very small and uncertain new lesions; cystic changes or necrosis in existing lesions), treatment may continue until the next scheduled assessment. If at the next scheduled assessment, progression is confirmed, the date of progression should be the earlier date when progression was suspected.

Table 3 Best Overall Response When Confirmation of CR and PR Required

Overall Response First Time Point	Overall Response Subsequent Time Point	Best Overall Response
CR	CR	CR
CR	PR	SD, PD or PR[a]
CR	SD	SD provided minimum criteria for SD duration met, otherwise, PD.
CR	PD	SD provided minimum criteria for SD duration met, otherwise, PD.
CR	NE	SD provided minimum criteria for SD duration met, otherwise, NE
PR	CR	PR
PR	PR	PR
PR	SD	SD
PR	PD	SD provided minimum criteria for SD duration met, otherwise, PD.
PR	NE	SD provided minimum criteria for SD duration met, otherwise, NE
NE	NE	NE

CR = complete response, PR = partial response, SD = stable disease, PD = progressive disease, NE = not evaluable.

a If a CR is truly met at first time point, then any disease seen at a subsequent time point, even disease meeting PR criteria relative to baseline, makes the disease PD at that point (since disease must have reappeared after CR). Best response would depend on whether minimum duration for SD was met. However, sometimes "CR" may be claimed when subsequent scans suggest small lesions were likely still present and in fact the patient had PR, not CR at the first time point. Under these circumstances, the original CR should be changed to PR and the best response is PR.

2.5. Frequency of tumor re-evaluation

Frequency of tumor re-evaluation while on treatment should be protocol specific and adapted to the type and schedule of treatment. However, in the context of phase 2 studies where the beneficial effect of therapy is not known, follow-up every 6 - 8 weeks (timed to coincide with the end of a cycle) is reasonable. Smaller or greater time intervals than these could be justified in specific regimens or circumstances. The protocol should specify which organ sites are to be evaluated at baseline (usually those most likely to be involved with metastatic disease for the tumor type under study) and how often evaluations are repeated Normally, all target and non-target sites are evaluated at each assessment. In selected circumstances certain non-target organs may be evaluated less frequently. For example, bone scans may need to be repeated only when complete response is identified in target disease or when progression in bone is

suspected.

After the end of the treatment, the need for repetitive tumor evaluations depends on whether the trial has as a goal the response rate or the time to an event (progression/death). If "time to an event" (e.g. time to progression, disease-free survival, progression-free survival) is the main endpoint of the study, then routine scheduled re-evaluation of protocol specified sites of disease is warranted. In randomised comparative trials in particular, the scheduled assessments should be performed as identified on a calendar schedule (for example: every 6 - 8 weeks on treatment or every 3 - 4 months after treatment) and should not be affected by delays in therapy, drug holidays or any other events that might lead to imbalance in a treatment arm in the timing of disease assessment.

2.6. Confirmatory Measurement/ Duration of Response

2.6.1. Confirmation

In non-randomized trials where response is the primary endpoint, confirmation of PR and CR is required to ensure responses identified are not the result of measurement error. This will also permit appropriate interpretation of results in the context of historical data where response has traditionally required confirmation in such trials. However, in all other circumstances, i.e. in randomized trials (phase 2 or 3) or studies where stable disease or progression are the primary endpoints, confirmation of response is not required since it will not add value to the interpretation of trial results. However, elimination of the requirement for response confirmation may increase the importance of central review to protect against bias, in particular in studies which are not blinded.

In the case of SD, measurements must have met the SD criteria at least once after study entry at a minimum interval (in general not less than 6 - 8 weeks) that is defined in the study protocol.

2.6.2. Duration of Overall Response

The duration of overall response is measured from the time measurement criteria are first met for CR/PR (whichever is first recorded) until the first date that recurrent or progressive disease is objectively documented (taking as reference for progressive disease the smallest measurements recorded on study).

The duration of overall complete response is measured from the time measurement criteria are first met for CR until the first date that recurrent disease is objectively documented.

2.6.3. Duration of Stable Disease

Stable disease is measured from the start of the treatment (in randomised trials, from date of randomisation) until the criteria for progression are met, taking as reference the smallest sum on study (if the baseline sum is the smallest, this is the reference for calculation of PD).

The clinical relevance of the duration of stable disease varies in different studies and diseases. If the proportion of patients achieving stable disease for a minimum period of time is an endpoint of importance in a particular trial, the protocol should specify the minimal time interval required between two measurements for determination of stable disease.

Note: The duration of response and stable disease as well as the progression-free survival are

influenced by the frequency of follow-up after baseline evaluation. It is not in the scope of this guideline to define a standard follow-up frequency. The frequency should take into account many parameters including disease types and stages, treatment periodicity and standard practice. However, these limitations of the precision of the measured endpoint should be taken into account if comparisons between trials are to be made.
